# Transsynaptic Coordination of Synaptic Growth, Function, and Stability by the L1-Type CAM Neuroglian

**DOI:** 10.1371/journal.pbio.1001537

**Published:** 2013-04-16

**Authors:** Eva-Maria Enneking, Sirisha R. Kudumala, Eliza Moreno, Raiko Stephan, Jana Boerner, Tanja A. Godenschwege, Jan Pielage

**Affiliations:** 1 Friedrich Miescher Institute for Biomedical Research, Basel, Switzerland; 2 Florida Atlantic University, Boca Raton, Florida, United States of America; Baylor College of Medicine, United States of America

## Abstract

The precise control of synaptic connectivity is essential for the development and function of neuronal circuits. While there have been significant advances in our understanding how cell adhesion molecules mediate axon guidance and synapse formation, the mechanisms controlling synapse maintenance or plasticity in vivo remain largely uncharacterized. In an unbiased RNAi screen we identified the *Drosophila* L1-type CAM Neuroglian (Nrg) as a central coordinator of synapse growth, function, and stability. We demonstrate that the extracellular Ig-domains and the intracellular Ankyrin-interaction motif are essential for synapse development and stability. Nrg binds to Ankyrin2 in vivo and mutations reducing the binding affinities to Ankyrin2 cause an increase in Nrg mobility in motoneurons. We then demonstrate that the Nrg–Ank2 interaction controls the balance of synapse growth and stability at the neuromuscular junction. In contrast, at a central synapse, transsynaptic interactions of pre- and postsynaptic Nrg require a dynamic, temporal and spatial, regulation of the intracellular Ankyrin-binding motif to coordinate pre- and postsynaptic development. Our study at two complementary model synapses identifies the regulation of the interaction between the L1-type CAM and Ankyrin as an important novel module enabling local control of synaptic connectivity and function while maintaining general neuronal circuit architecture.

## Abstract 2

Experiments in peripheral and central synapses reveal the regulatory mechanisms that enable trans-synaptic control of synapse development and maintenance by the L1-type CAM Neuroglian.

## Author Summary

The function of neuronal circuits relies on precise connectivity, and processes like learning and memory involve refining this connectivity through the selective formation and elimination of synapses. Cell adhesion molecules (CAMs) that directly mediate cell–cell interactions at synaptic contacts are thought to mediate this structural synaptic plasticity. In this study, we used an unbiased genetic screen to identify the *Drosophila* L1-type CAM Neuroglian as a central regulator of synapse formation and maintenance. We show that the intracellular Ankyrin interaction motif, which links Neuroglian to the cytoskeleton, is an essential regulatory site for Neuroglian mobility, adhesion, and synaptic function. In motoneurons, the strength of Ankyrin binding directly controls the balance between synapse formation and maintenance. At a central synapse, however, a dynamic regulation of the Neuroglian–Ankyrin interaction is required to coordinate transsynaptic development. Our study identifies the interaction of the L1-type CAM with Ankyrin as a novel regulatory module enabling local and precise control of synaptic connectivity without altering general neuronal circuit architecture. This interaction is relevant for normal nervous system development and disease as mutations in L1-type CAMs cause mental retardation and psychiatric diseases in humans.

## Introduction

Transsynaptic interactions mediated by cell adhesion molecules (CAMs) control the formation, function, and stability of synaptic connections within neuronal circuits. While a large number of synaptogenic CAMs controlling the initial steps of synapse formation have been identified [1],[2], we have only limited knowledge regarding the identity or regulation of CAMs selectively controlling synapse maintenance or plasticity. Information processing within neuronal circuits is adjusted by the selective addition or elimination of individual synapses both during development and in response to activity [3],[4]. These changes in connectivity can occur in very close proximity to stable synapses [5],[6] indicating the existence of mechanisms capable of local alterations of transsynaptic adhesion. Potential mechanisms to alter binding affinities of CAMs include direct alterations of extracellular domains through binding of ligands like metal ions (e.g., Ca^2+^) or indirect mechanisms through the selective association of CAMs with the intracellular cytoskeleton via adaptor proteins [7]. Modulation of intracellular interactions via posttranslational modifications can alter mobility, clustering, and adhesive force of CAMs [8]. For example, it has been demonstrated for the Cadherin–Catenin complex that changes in biophysical properties can induce changes in synapse morphology, strength, or stability and modulate transsynaptic signaling [9],[10].

To identify cell adhesion molecules potentially controlling synapse maintenance and plasticity we performed an unbiased in vivo RNA interference (RNAi) screen at the larval neuromuscular junction (NMJ) of 287 transmembrane proteins that are predicted to function as synaptic CAMs based on their domain structure [11]. These included Ig-domain containing proteins, Leucine-rich repeat proteins, Cadherins, Integrins, Semaphorins, and others (Table S1). In this high-resolution screen we identified the *Drosophila* L1-type CAM Neuroglian (Nrg) as a key regulator for synapse stability. Nrg encodes the *Drosophila* ortholog of the L1-type protein family [12] that is composed of four closely related members in vertebrates: L1, CHL1 (close homolog of L1), NrCAM (neuronal CAM), and Neurofascin [13],[14]. L1-type IgCAMs usually consist of 6 Ig-domains, 3–5 fibronectin type III domains, a single transmembrane domain, and an intracellular tail. The extracellular domain of L1 family proteins can mediate cell–cell adhesion via homophilic interactions and can also engage in a variety of heterophilic interactions with other Ig-domain proteins (e.g., NCAM, TAG-1, Contactin, and others), extracellular matrix proteins, or integrins [13]–[15]. The intracellular tail contains distinct protein–protein interaction domains potentially controlling the localization and function of L1 proteins [16]–[18]. Most prominent is a central Ankyrin interacting motif that is highly conserved among all vertebrate L1 family proteins and *Drosophila* Neuroglian [16],[19]. Phosphorylation of the tyrosine within this FIGQY motif abolishes binding to Ankyrins [20]–[23]. The Ankyrin-binding domain is essential for mediating neuronal function in vivo in *C. elegans*, however it is dispensable for L1-mediated homophilic adhesion in transfected cells in culture [24],[25].

Importantly, we previously identified *Drosophila ankyrin2* (*ank2*) as an essential gene for synapse stability at the larval neuromuscular junction [26],[27]. Ank2 together with the presynaptic spectrin cytoskeleton and the actin capping protein Hts/Adducin controls NMJ formation and maintenance and provides a scaffold to link the actin and microtubule cytoskeleton to synaptic cell adhesion molecules [28],[29]. Based on this potential biochemical interaction Nrg might encode the CAM upstream of this Ank2/Spectrin scaffold to control synapse development.

Human mutations in L1CAM cause a broad spectrum of neurological disorders (L1 or CRASH syndrome) including MASA syndrome (mental retardation, aphasia, shuffling gait, adducted thumbs), agenesis of the corpus callosum, and spastic paraplegia. In addition, hypomorphic mutations in L1CAM and NrCAM have been linked to psychiatric diseases [14],[15],[30]. In correspondence with the human disease, animal models implicated L1-type proteins in nervous system development [13],[14]. At the cellular level L1-type proteins are involved in the control of neurite outgrowth, axon pathfinding, and fasciculation and synapse development [14],[16],[31]. The subcellular localization of L1-type proteins contributes to the establishment and maintenance of specialized neuronal membrane compartments including the axon initial segment (AIS) and nodes of Ranvier [32]–[35]. While these studies highlight essential functions of L1-type proteins, potential redundant or antagonistic functions between different L1-type proteins may mask the full extent of their importance for nervous system development. Indeed, evidence for redundant functions between L1-type proteins was provided by a double mutant analysis of L1CAM and NrCAM [36]. Together with the requirement of L1-type proteins for early nervous system development, this confounds our current understanding of the contribution of L1-type CAM to synapse development and plasticity. In addition, mechanistic insights into the in vivo control of L1-type CAM function at synapses are lacking to date.

Nrg encodes the sole homolog of L1-type CAMs in *Drosophila* with equal homology to all four vertebrate proteins. This provides a unique opportunity to unravel the contributions and mechanisms of regulation of L1-type CAMs in synapse development and maintenance. Here, we generate a series of Pacman-based mutations that allowed us to identify the specific contributions of extra- and intracellular domains of Nrg for synapse stability. We then provide evidence that binding of Nrg to Ank2 is critical for the control of mobility of Nrg in vivo. We demonstrate that modulation of the Nrg–Ank2 interaction allows precise control over the balance between synapse formation and stability. Finally, we demonstrate that dynamic regulation of the Ankyrin-binding domain of Nrg is essential for the coordination of pre- and postsynaptic development via transsynaptic signaling mechanisms at central synapses.

## Results

### RNAi Screen Identifies the *Drosophila* L1-Type CAM Neuroglian as Essential for Synapse Stability

**Figure 1:**
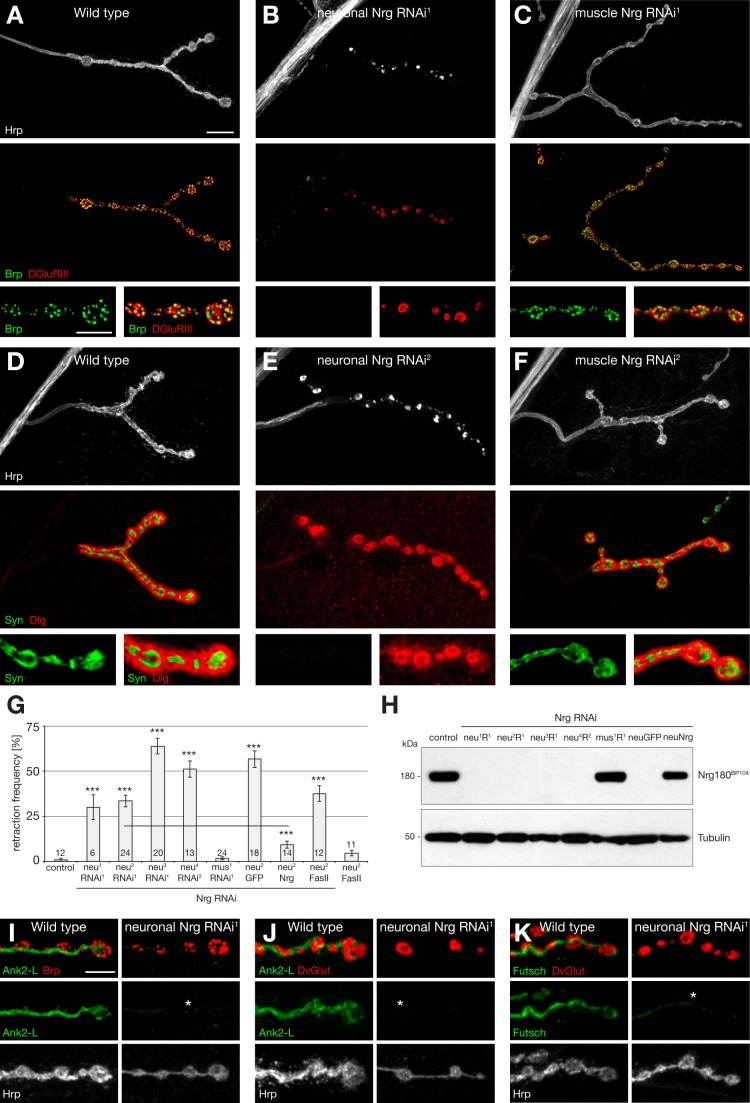
Presynaptic Nrg is essential for synapse stability. (A–C) NMJs on muscle 4 stained for the presynaptic motoneuron membrane (Hrp, white), the presynaptic active zone marker Brp (green), and postsynaptic glutamate receptors (DGluRIII, red). (A) A stable wild-type NMJ indicated by perfect apposition of pre- and postsynaptic markers. (B) Knockdown of presynaptic Nrg resulted in severe synaptic retraction indicated by the loss of presynaptic Brp despite the presence of postsynaptic glutamate receptors and by a fragmentation of the presynaptic membrane. Synaptic retractions caused a characteristic fusion of postsynaptic glutamate receptor clusters. (C) Knockdown of postsynaptic Nrg did not impair synapse stability. (D–F) NMJs on muscle 4 stained for the presynaptic motoneuron membrane (Hrp, white), presynaptic vesicles (Syn, green), and postsynaptic Dlg (red). Identical phenotypes were observed when using an independent Nrg RNAi line and independent pre- and postsynaptic markers. Only presynaptic knockdown of Nrg resulted in synaptic retractions indicated by a selective loss of synaptic vesicles and a fragmentation of the presynaptic membrane. (G) Quantification of synaptic retractions. Neuronal but not muscle-specific knockdown of Nrg using different Gal4 driver combinations or independent RNAi constructs resulted in a significant increase in synaptic retractions on muscle 4. The synaptic retraction frequency was significantly rescued (*p*≤0.001) by co-expression of *UAS–nrg180* but not when we co-expressed either UAS–*mCD8*–*GFP* or UAS–*fasII*. Neuronal expression of UAS–*fasII* in a wild-type background (neu^2^ FasII) did not result in a significant increase in synaptic retractions (genotypes: neu^1^ = *elav^C155^*–Gal4; neu^2^ = *elav^C155^*–Gal4; *ok371*–Gal4; neu^3^ = *elav^C155^*–Gal4; UAS-*dcr2*; neu^4^ = *elav^C155^*–Gal4; *sca*–Gal4 UAS–*dcr2*; mus^1^ = UAS–*dcr2*; *mef2*–Gal4; RNAi^1^ = VDRC6688; RNAi^2^ = VDRC107991; GFP, Nrg, and FasII indicate co-expression of the corresponding UAS construct; the number of analyzed animals is indicated). (H) Western blot analysis of the genotypes in (G) probed with an antibody against Nrg180 (Nrg180^BP104^). Neuronal but not muscle-specific Nrg RNAi resulted in efficient knockdown of Nrg180 in larval brains. Nrg180 levels could be rescued by co-expression of Nrg180 but not by co-expression of mCD8-GFP. (I–K) Characterization of multiple presynaptic markers after knockdown of presynaptic Nrg. (I) In wild-type animals presynaptic Ank2-L (green) and Brp (red) were present in all synaptic boutons. In the absence of Nrg, Ank2-L was lost prior to Brp at distal parts of an NMJ that was still stable as indicated by the continuous membrane staining. (J) Similarly, Ank2-L was lost prior to the presynaptic vesicle marker DvGlut (red) at a semistable NMJ. (K) In wild-type animals the microtubule-associated protein Futsch (green) and DvGlut (red) were present in all boutons. Knockdown of Nrg resulted in a loss of Futsch prior to the disassembly of DvGlut at early stages of synapse retraction. Scale bar in (A) corresponds to (A–F), 10 µm, inset 5 µm. Scale bar in (I) corresponds to (I–K), 5 µm. Error bars represent SEM.

To identify cell adhesion molecules necessary for the maintenance of synaptic connections we performed a transgenic RNAi-based screen [37] of 287 transmembrane proteins encoding potential cell adhesion molecules based on their domain structure and previously described functions in axon guidance or synapse development (Table S1). We knocked down candidate genes simultaneously in presynaptic neurons and postsynaptic muscles and analyzed third instar NMJs for defects in synapse stability using selective pre- and postsynaptic markers (Figure 1A–F). In wild-type animals the presynaptic active zone marker Bruchpilot (Brp) is found in close opposition to postsynaptic glutamate receptor cluster at all individual synapses within the presynaptic nerve terminal demarcated by the membrane marker Hrp. In contrast, NMJs displaying postsynaptic glutamate receptor clusters without opposing presynaptic active zone markers and a fragmentation of the presynaptic membrane indicate synapse retractions [27],[28]. We identified *Drosophila* Nrg as the major hit in our screen resulting in synaptic retractions at more than 50% of all NMJs on muscle 4. We first tested whether specific knockdown of Nrg either in the motoneuron or the muscle also impairs synapse stability. Presynaptic knockdown of Nrg was sufficient to cause synaptic retractions equivalent to the simultaneous pre- and postsynaptic knockdown (Figure 1B,G). In contrast, muscle-specific *nrg* RNAi did not lead to a significant increase in synaptic retractions (Figure 1C,G). We obtained similar results when we expressed an independent *nrg* RNAi line (Figure 1E–G) and were able to enhance the phenotype by combining different motoneuron Gal4 drivers or by co-expressing UAS-*dcr2* to enhance RNAi efficacy (Figure 1G). In addition, we observed similar rates and severities of synapse retractions when using independent pre- and postsynaptic markers and when analyzing different subsets of muscles (Figure 1D–F; Figure S1; Table S2).

**Figure 2:**
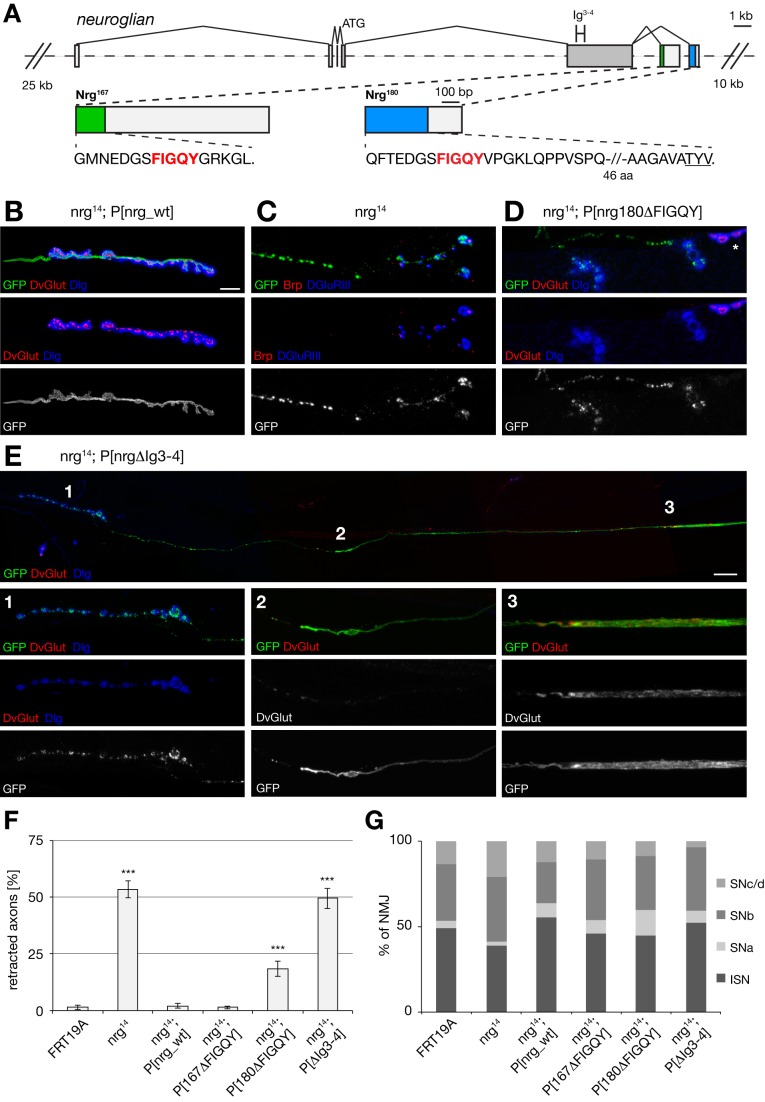
MARCM analysis demonstrates requirement of extra- and intracellular domains of Nrg180 for synapse stability. (A) Overview of the genomic locus of *nrg*. The Pacman construct spans 92 kb including the endogenous enhancer elements up- and downstream of *nrg*. The Nrg167- and Nrg180-specific exons and the relevant amino acid sequences are depicted. The position of the common Ig-domains 3 and 4 is indicated. The isoform-specific FIGQY sequences are highlighted in red and the PDZ protein-binding motif of Nrg180 isoform is underlined. (B) A *nrg^14^* MARCM clone rescued by a wild-type *nrg* Pacman construct. The motoneuron clone was marked by the expression of mCD8–GFP (green). Synaptic vesicles (DvGlut, red) were found opposite postsynaptic Dlg (blue), indicating a stable NMJ. (C) A *nrg^14^* MARCM clone showing a synaptic retraction. Only fragmented remnants of the membrane GFP marker were still present at a nerve terminal that almost completely lacked the presynaptic active zone marker Brp (red). Postsynaptic glutamate receptor clusters were still present. In addition, the axonal membrane prior to the NMJ was also fragmented. (D) A *nrg^14^* MARCM clone expressing only Nrg180 lacking the FIGQY motif. The mutant (GFP-positive) NMJ was retracted while a neighboring control NMJ (asterisk) remained stable. (E) Composite image overview of an *nrg^14^* MARCM clone expressing a mutated form of Nrg180 lacking the extracellular Ig3–4 domains. Three areas are shown at larger magnification in 1–3. (E^1^) At the NMJ no presynaptic vesicles were present opposite postsynaptic Dlg. The Dlg staining was no longer interconnected and only remnants of the presynaptic membrane marker mCD8–GFP were visible, indicating a complete elimination. (E^2^) Approximately 150 µm proximal from the NMJ, the axon ended in a “bulb-like” structure. Between the “bulb-like” axon ending and the NMJ, only punctate staining of the membrane marker was visible. (E^3^) At a significant distance from the “bulb,” a large axonal swelling was visible that contained aggregates of the synaptic vesicle marker DvGlut. (F) Quantification of mCD8-marked axons that were not connected to target muscles in the indicated genetic background. The *nrg^14^* mutant phenotype was significantly rescued by the presence of a wild-type *nrg* Pacman construct, a *nrg* construct lacking the FIGQY domain of Nrg167, and only partially rescued by P[*nrg180^ΔFIGQY^*] and not rescued by P[*nrg180^ΔIg3–4^*]. (G) Analysis of muscle innervation pattern of motoneuron MARCM clones that were connected to postsynaptic muscles. In all genotypes we observed normal innervation patterns for all four major classes of motoneurons. Scale bar in (B) corresponds to (B-D), 10 µm. Scale bar in (E), 20 µm. Error bars represent SEM.

To monitor the efficiency of our RNAi mediated knockdown, we directly analyzed Nrg protein levels. *Drosophila nrg* encodes two specific isoforms, the ubiquitous isoform Nrg167 and the neuronal specific isoform Nrg180 (Figure 2A) [38]. Nrg180 was present throughout motoneuron axons and within the presynaptic nerve terminal (Figure S2A), and in addition, Nrg167 was present in muscles and glial cells (Figure S2E). Western blots of larval brain extracts and analysis at the larval NMJ demonstrated that all combinations of presynaptic *nrg* RNAi efficiently knocked down Nrg180 (Figure 1H; Figure S2B). Similarly, muscle-specific knockdown of Nrg caused a loss of Nrg^3c1^ staining in the muscle that can be attributed to a loss of Nrg167 (Figure S2F). The loss of postsynaptic Nrg resulted in a significant change in presynaptic Nrg levels and distribution at the NMJ (Figure S2C, F; reduction to 62.9±3.7% of wild-type protein within the presynaptic terminal, *p*<0.001), indicating a requirement of postsynaptic Nrg for presynaptic Nrg localization. However, this reduction in presynaptic Nrg180 levels was not sufficient to impair synapse stability (Figure 1C,F,G), implicating the potential existence of alternative postsynaptic Nrg interaction partners essential for NMJ maintenance [39]. Likewise, presynaptic knockdown of Nrg reduced Nrg staining in the axon and at the synaptic terminal but did not significantly alter Nrg distribution within the postsynaptic subsynaptic retriculum (SSR) (Figure S2G,H). To validate the specificity of our RNAi-mediated knockdown we aimed to rescue the synaptic phenotypes by co-expressing wild-type Nrg180. Simultaneous expression of UAS–*nrg180* significantly rescued synapse stability by restoring Nrg180 protein levels both in larval brains and at the NMJ (Figure 1G,H and Figure S2D). In contrast, co-expressing UAS–*mCD8*–*GFP* or UAS–*fasciclinII* (NCAM homolog) failed to rescue the synaptic retraction phenotype (Figure 1G). Thus, the specific loss of pre- but not postsynaptic Nrg caused an impairment of synapse stability.

To gain insights into the molecular processes inducing synaptic retractions in animals lacking presynaptic Nrg, we analyzed the distribution of two presynaptic components, Ank2 and Futsch, at early stages of synaptic retractions in comparison to active zone and vesicle markers. The presynaptic adaptor protein Ank2 is an essential molecule for synapse stability and Ankyrins can directly bind to Nrg [26],[27],[40]. The microtubule-associated protein Futsch serves as a marker for presynaptic microtubules as loss of microtubules represents an early step in synaptic retractions at the *Drosophila* NMJ [27],[29],[41]. At wild-type NMJs, Ank2 and Futsch were present in all terminal boutons together with Brp and DvGlut. In contrast, after presynaptic knockdown of Nrg, we observed NMJs lacking Ank2 or Futsch in terminal boutons that still contained presynaptic Brp or DvGlut (Figure 1I–K). Thus loss of Ank2 and of the associated microtubule cytoskeleton may represent early steps during synapse retractions caused by the loss of Nrg.

### Distinct Contributions of the Extra- and Intracellular Domain of Nrg for Synapse Stability

Two domains of Nrg that may be essential for synapse maintenance are the extracellular Ig domains mediating association with postsynaptic CAMs and the intracellular Ankyrin binding domain that provides a link to the presynaptic cytoskeleton. To directly test for a potential role of these domains we generated genomic rescue constructs that allow expression of wild-type and mutated *nrg* at endogenous levels using a site-directed Pacman-based approach [42],[43]. We first generated a transgenic construct encompassing the entire *nrg* locus including 25 kb upstream and 10 kb downstream regulatory sequences (P[nrg_wt]; Figure 2A). This construct fully rescued the embryonic lethal *nrg* null mutations *nrg^14^* and *nrg^17^*. We then used *galK*-mediated recombineering [44] to generate a deletion of the extracellular Ig domains 3 and 4 (P[*nrg^ΔIg3–4^*]), thereby completely disrupting hetero- and homophilic binding capacities of Nrg [45]. In addition, we generated specific deletions of the Ankyrin-binding domains of Nrg167 and Nrg180 that are encoded by unique exons (P[*nrg167^ΔFIGQY^*] and P[*nrg180^ΔFIGQY^*]; Figure 2A). All constructs were inserted into the genomic insertion site attP40 to ensure identical expression levels. While P[*nrg167^ΔFIGQY^*] and P[*nrg180^ΔFIGQY^*] rescued the embryonic lethality of *nrg^14^* mutants similar to the wild-type construct, P[*nrg^ΔIg3–4^*] failed to rescue lethality. In order to analyze the larval NMJ of *nrg^14^*; P[*nrg^ΔIg3–4^*] mutant animals, we combined the Pacman rescue approach with the MARCM technique [46]. This allows the generation of mCD8–GFP-marked motoneurons expressing only the mutated form of Nrg. First we analyzed motoneurons completely lacking *nrg* using the *nrg^14^* null mutation and observed two striking phenotypes. We found synapse retractions indicated by NMJs displaying remnants of the presynaptic MARCM membrane marker opposite postsynaptic glutamate receptors but lacking the presynaptic marker Brp (Figure 2C). While synapse retractions were only observed at low frequency, about 50% of all MARCM motoneuron axons ended in “bulb-like” structures within nerve bundles and were not connected to a postsynaptic muscle (Figure 2F and Figure S3E). The wild-type *nrg* Pacman construct fully rescued the axonal and NMJ phenotypes (Figure 2B,F and Figure S3A,D), however, P[*nrg^ΔIg3–4^*] failed to rescue these defects and the presence of P[*nrg180^ΔFIGQY^*] resulted only in a partial rescue. In both genotypes we observed synaptic retractions as well as axons ending in bulbs distant from a potential target muscle (Figure 2D–F; Figure S3C,G; Table S1). In contrast, no defects were observed in the presence of P[*nrg167^ΔFIGQY^*] indicating that only the Ankyrin binding motif of Nrg180 is essential within motoneurons (Figure 2F; Figure S3B,F; Table S2).

Prior studies showed a delay of axonal outgrowth in *nrg* mutant embryos [47]–[49]. Our axonal phenotypes would be equally consistent with a stalling of axons or with a retraction of axons after initial innervation of target muscles. Importantly, we observed mutant MARCM motoneurons where we could link synapse eliminations to axons ending in bulb-like structures. Figure 2E shows an example of a complete elimination of an NMJ indicated by the loss of presynaptic vesicles, while fragments of the mCD8–GFP-marked motoneuron membrane were still present opposite postsynaptic Dlg. We traced fragmented membrane remnants over a distance of more than 150 µm to the retraction bulb-like structure (Figure 2E; see Figure S3C for another example). Additionally, we observed large axonal swellings in the same axon further proximal toward the cell body of the motoneuron (Figure 2E). Rates of retraction bulbs and axonal swellings were identical in MARCM clones of *nrg^14^* and *nrg^14^*; P[*nrg180^ΔIg3–4^*] animals (Figure 2F). Finally, analysis of the innervation pattern of motoneuron axons forming stable NMJs at this larval stage demonstrated that loss of Nrg did not result in obvious axon guidance defects. We observed similar rates of innervations for all four major classes of motoneurons in all genotypes (Figure 2G and Figure S2H). In summary, while we cannot exclude a role for Nrg in axonal outgrowth, we provide clear evidence that Nrg is required for the maintenance of the NMJ and that this function requires both the extracellular domain and the intracellular Ankyrin-binding domain of Nrg180 but not of Nrg167.

### Mutations in the Nrg FIGQY Motif Differentially Affect Ankyrin2-Binding

Based on these results we aimed to unravel the molecular mechanisms controlling the synaptic function of Nrg through the interaction with the Ankyrin-associated cytoskeleton. Prior studies in vertebrates demonstrated that phosphorylation of the conserved tyrosine residue within the FIGQY motif of L1-type proteins has the potential to abolish the interaction with Ankyrins [20]–[23]. Similarly, Yeast-2-Hybrid assays showed that Nrg can bind to *Drosophila* Ankyrin1 and Ankyrin2 and that replacing the tyrosine with a phenylalanine (Y-F) reduces binding capacities [50].

**Figure 3:**
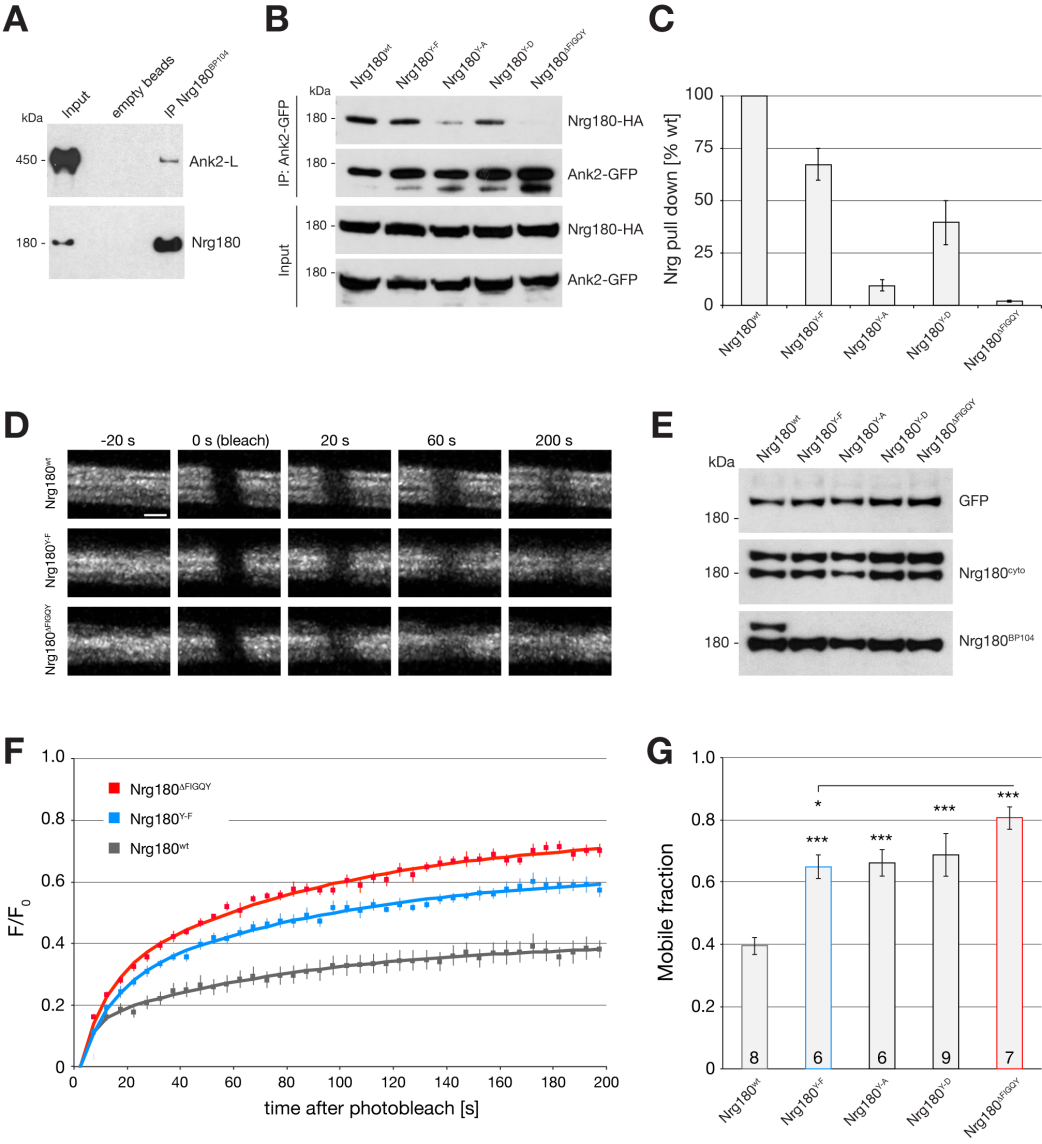
Mutations in the FIGQY–Ankyrin binding motif alter the Nrg–Ank2 interaction and increase Nrg mobility in vivo. (A) Co-immunoprecipition (Co-IP) of Ank2-L from larval brain by Nrg180. IP of Nrg180 using the neuronal-specific antibody Nrg^BP104^ co-precipitates the large Ank2-L isoform (450 kDa) from larval brain extracts. No Ank2-L signal is observed when using empty beads. (B) IP of Nrg180-HA proteins using Ank2-S–GFP from co-transfected S2-cells. Ank2-S pulled down wild-type Nrg180-HA efficiently. Mutations in the FIGQY domain differentially affected binding efficiency. Western blots show IPs and input controls. (C) Quantification of four independent IP experiments demonstrated reduced Ank2 binding due to the specific mutations within the FIGQY motif. (D–G) Fluorescence recovery after photobleaching (FRAP) experiments using GFP-tagged versions of wild-type and mutant forms of Nrg180 at the NMJ. (D) Representative recoveries of FRAP in motoneurons for Nrg180^wt^, Nrg180^Y-F^, and Nrg180^ΔFIGQY^. (E) Equal levels of all GFP-tagged constructs were expressed in motoneurons (*ok371*–Gal4) in wild-type animals as demonstrated by Western blot analysis. The Nrg180^cyto^ antibody recognizes both wild-type Nrg180 and Nrg180–GFP. In contrast, Nrg180^BP104^ recognizes endogenous Nrg180 but only the wild-type version of Nrg180–GFP. This indicates that Nrg^BP104^ binds specifically to the Nrg180 FIGQY motif and any alteration of the tyrosine will abolish protein recognition. (F and G) Recovery curves of multiple independent FRAP experiments were fitted to a double exponential curve and used to calculate the mobile fraction of Nrg180. Wild-type Nrg180 recovered to about 40% within the 200 s time frame. The mobility of Nrg180 was significantly increased (more than 1.5×) when the FIGQY motif was mutated (Nrg180^Y-F^, Nrg180^Y-A^, Nrg180^Y-D^). An almost 2-fold increase in mobility was observed after deletion of the Ankyrin-binding motif (Nrg180^ΔFIGQY^). Numbers in F represent number of independent experiments analyzed. Scale bar in (D) represents 5 µm. Error bars represent SEM.

Therefore, we first tested whether the neuronal isoform Nrg180 can directly bind to the large isoform of Ank2 (Ank2-L) that is present within the presynaptic nerve terminal (Figure 1I,J). Using the Nrg180-specific antibody Nrg180^BP104^ we were able to co-immunoprecipitate Ank2-L from larval brain extracts demonstrating that Nrg180 and Ank2-L interact in vivo (Figure 3A). Next we used IP-assays to further characterize the interaction between Nrg180 and Ank2. We generated tagged Nrg180 and Ank2 UAS constructs (Ank2-S, short isoform of Ank2 containing all potential Nrg interacting domains) and co-expressed the constructs in *Drosophila* S2 cells. We were able to efficiently pull-down Nrg180 using Ank2-S and vice versa (Figure 3B and unpublished data). To alter the binding properties and to potentially mimic a gradual increase in phosphorylation levels of Nrg180 in vivo, we generated a series of mutations replacing the tyrosine with a phenylalanine (Y-F), aspartate (Y-D), or alanine (Y-A) or by deleting the entire FIGQY motif (ΔFIGQY). Compared to wild-type we observed a 30% decrease in binding capacities for the Y-F, a 70% decrease for the Y-D, and a 90% reduction for the Y-A mutation. The deletion of the FIGQY motif essentially abolished the Nrg–Ank2 interaction (Figure 3B,C). Thus, we have identified a series of mutations that allows us to characterize the function and regulation of Nrg in vivo. These mutations potentially allow the differentiation between processes depending on Nrg bound to Ankyrins and processes depending on a differential regulation of the FIGQY motif.

### Loss of Ankyrin2 Binding Results in Increased Lateral Mobility of Nrg

Studies in *Drosophila* and vertebrates demonstrated that impairing the interaction of CAM with the cytoskeleton results in an increase in lateral mobility and a simultaneous reduction of adhesive properties [20],[51],[52]. To test whether Nrg is regulated in a similar manner we analyzed the impact of the FIGQY-mutations on the biophysical behavior of Nrg in vivo. Therefore, we generated GFP-tagged UAS-constructs of all *nrg180*-*FIGQY* mutations and used site-specific integration to generate transgenic flies that will express equal protein levels after Gal4 activation. Analysis of larval brain extracts after expression of the constructs in motoneurons (*ok371*–Gal4) in a wild-type background demonstrated equal protein levels comparable to endogenous nontagged Nrg180 (Figure 3E upper and middle panels, analysis with an anti-GFP antibody and with a newly generated anti-Nrg180^cyto^ antibody that recognizes the cytoplasmic tail of Nrg180 C-terminal to the FIGQY motif). The Nrg180-specific antibody Nrg180^BP104^ specifically recognizes the Nrg180 FIGQY motif as the antibody detects wild-type GFP-tagged Nrg180 but not any mutant proteins (Figure 3E, lower panel). We used fluorescence recovery after photobleaching (FRAP) to test whether the FIGQY mutations affect the mobility of Nrg180-GFP within motoneurons in vivo. For wild-type Nrg180 we observed a mobile fraction of about 40% of total protein (Figure 3D,F,G). The deletion of the Ank2 binding domain increased the mobile fraction of Nrg180 by a factor of two to about 80%. For the tyrosine-specific point mutations, we observed a significant increase in the mobile fraction compared to wild type but to a lesser extent than for Nrg180^ΔFIGQY^ (Figure 3D,F,G). Thus, selectively impairing the interaction between Nrg180 and Ank2 significantly changes the mobility of Nrg180 in motoneurons in vivo.

### Association of Nrg180 with Ankyrin2 Balances NMJ Growth and Stability

To address the relevance of this Nrg180–Ank2 interaction in vivo, we introduced all FIGQY-specific mutations into the wild-type *nrg* Pacman construct. In addition, we generated a deletion of the Nrg180 specific C-terminus including the FIGQY motif, a complete deletion of the FIGQY motif of Nrg167 as well as a specific deletion of the last three amino acids of Nrg180 as this potential PDZ-protein interacting domain has been implicated in axon outgrowth of mushroom body neurons (Figure 2A and Table S2) [53]. All constructs were inserted into the attP40 genomic landing site and crossed into the background of the *nrg^14^* null mutation to create flies that express only mutant Neuroglian protein under endogenous control, thereby mimicking the effect of knock-in mutations. All modifications of the intracellular cytoplasmic domains of Nrg167 and Nrg180 rescued the embryonic lethality associated with *nrg* null mutations (*nrg^14^* and *nrg^17^*), allowing an analysis at the third instar larval stage. To confirm the specificity of our mutations and the expression levels and localization of the mutant proteins, we analyzed the animals with specific antibodies recognizing either both isoforms of Nrg (Nrg^3c1^) or only Nrg180 (Nrg180^BP104^) [38]. As observed for the UAS-constructs, Nrg180^BP104^ recognizes only wild-type Nrg180 but none of our FIGQY mutations (Figure S4A,B; Figure 3E; unpublished data), thereby demonstrating that our Pacman-rescued flies indeed only express the mutant version of the protein and no wild-type Nrg180 protein. We verified this using the Nrg180^cyto^ antibody as well as a second new antibody, Nrg^FIGQY^, that recognizes the wild-type FIGQY motif of both Nrg167 and Nrg180 but not any mutant versions (Figure S4A,B). We were able to unambiguously identify all mutated proteins to demonstrate that all constructs are expressed at wild-type levels within larval brains (Figure S4B,D). In addition, all constructs enabled Nrg180 localization to the presynaptic nerve terminal and Nrg167 expression within glial cells and muscles (Figure S4A,C). Our data demonstrate that the FIGQY domain is not essential for presynaptic localization of Nrg180.

**Figure 4:**
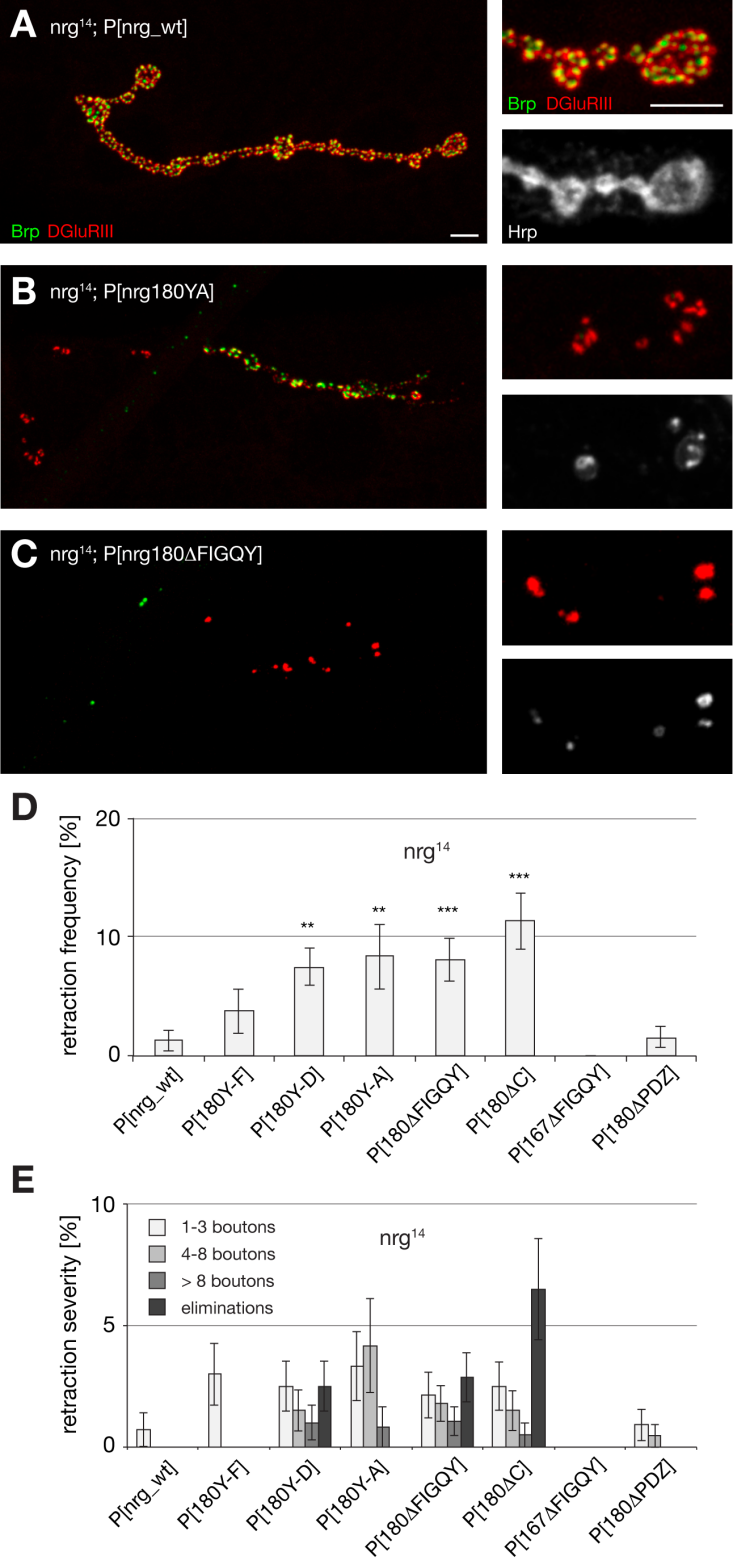
Impairment of Ank2 binding results in synaptic retractions. (A–C) Analysis of synapse stability in *nrg^14^* null mutant animals expressing different *nrg* Pacman constructs using the presynaptic marker Brp (green), postsynaptic DGluRII (red), and the presynaptic membrane Hrp (white). (A) Animals carrying the wild-type nrg Pacman in the *nrg^14^* mutant background had stable NMJs. (B) Presence of the Nrg180^Y-A^ mutation resulted in increased synaptic retraction rates. (C) Animals lacking the Ank2 binding motif FIGQY showed synaptic retractions including complete eliminations. (D and E) Quantification of retraction frequency and severity demonstrated increasing levels of synaptic retractions correlating with the gradual loss of Ank2 binding capacities of Nrg180. Deletion of the Nrg167–FIGQY motif or the PDZ protein binding domain of Nrg180 did not result in a significant increase in retraction frequency or severity (*n* = 12–22 animals). Asterisks indicate *p*≤0.01 for ** and *p*≤0.001 for ***. Scale bar in (A) corresponds to (A–C), 10 µm, inset 5 µm. Error bars represent SEM.

Next, we systematically determined the requirement of the different domains for synapse development in third instar larvae. Our analysis of synapse stability revealed a significant increase in the frequency and severity of synaptic retractions in mutants with severely disrupted Nrg180–Ank2 interactions (*nrg180^Y-D^*, *nrg180^Y-A^*, *nrg180^ΔFIGQY^*) but not in animals expressing Nrg180 with only slightly impaired Ank2 binding capacities (*nrg180^Y-F^*) (Figure 4A–E and Table S2). The FIGQY motif of Nrg167 and the PDZ protein-binding domain of Nrg180 are not essential for normal NMJ development (Figure 4D,E; Figure S5B; Table S1). These phenotypes are consistent with our results for the *nrg^14^*; P[*nrg180^ΔFIGQY^*] and *nrg^14^*; P[*nrg167^ΔFIGQY^*] MARCM clones (Figure 2F and Table S2). The observation that impairing the Nrg–Ank2 interaction only in motoneurons weakened synapse stability provided an alternative way to test for a potential contribution of postsynaptic (muscle) Nrg for synapse maintenance. Therefore, we selectively knocked down Nrg in muscles of *nrg^14^*; P[*nrg_wt*] or *nrg^14^*; P[*nrg180^ΔFIGQY^*] animals. Indeed, we observed a significant increase in the frequency and severity of synaptic retraction when knocking down postsynaptic Nrg in the sensitized animals lacking the Ank2 interaction domain but not in animals expressing the wild-type Pacman construct (Figure S6A–D).

We next asked whether the Nrg180 FIGQY motif is required for the synaptic localization of Ank2-L to mediate NMJ stability. Interestingly, we did not observe obvious alterations of presynaptic Ank2-L localization or protein levels in P[*nrg*_*wt*, *180Y-F*, or *180ΔFIGQY*] mutant animals at stable synapses when compared to control animals (Figure S7A; *p*>0.05 for comparison of protein levels; unpublished data). Thus, Nrg and Ank2 do not depend on each other for initial synaptic localization but display a high sensitivity toward normal levels of their interaction partner as they are among the first proteins to be lost at *ank2* or *nrg* mutant semi-stable NMJs (Figure 1I,J and Figure S7B–D).

**Figure 5:**
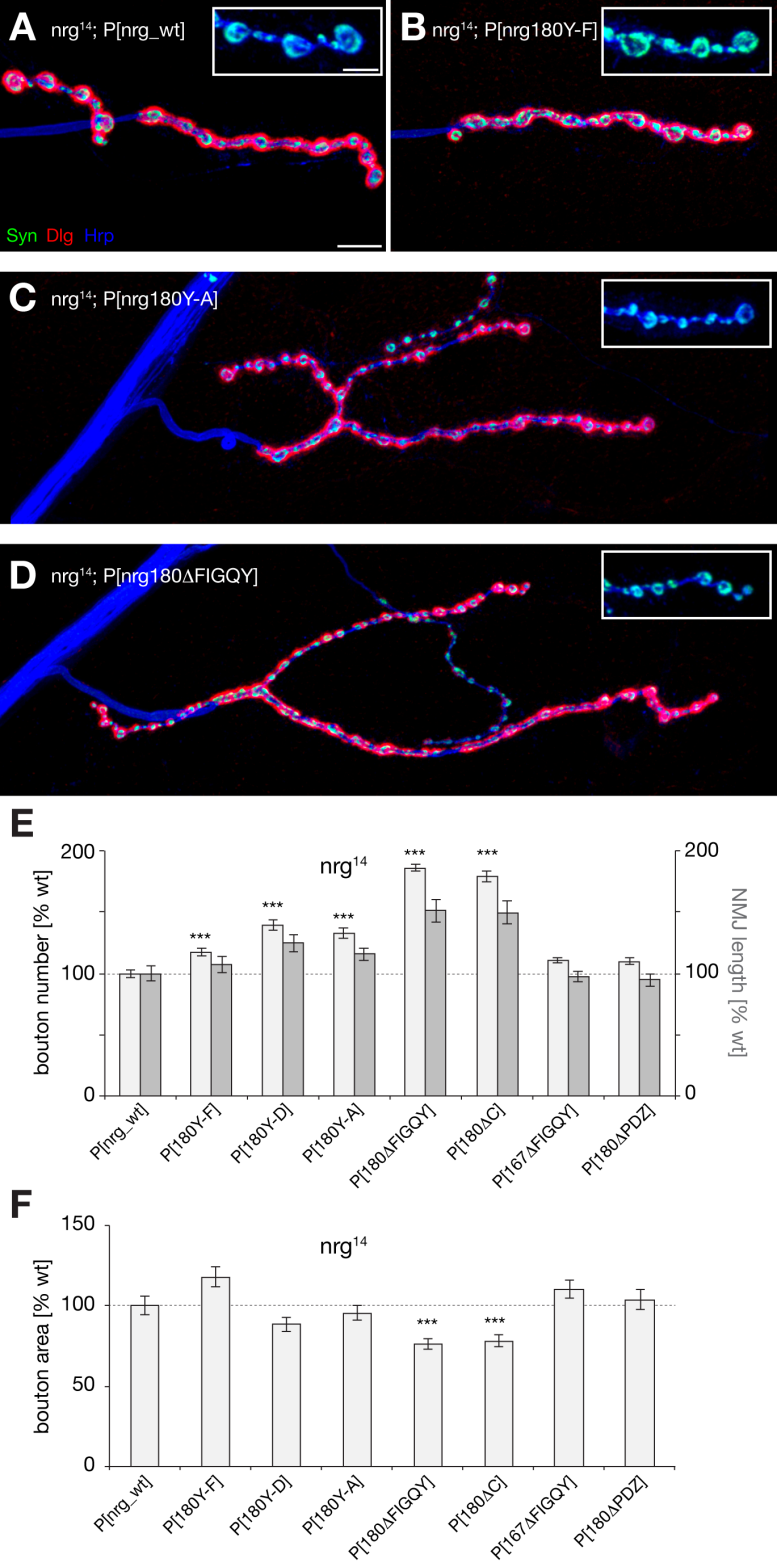
Impairment of Ank2 binding of Nrg180 increases NMJ growth. Analysis of NMJ growth in *nrg^14^* mutant animals expressing different mutated *nrg* Pacman constructs using the presynaptic vesicle marker Synapsin (Syn, green), the postsynaptic marker Dlg (red), and a marker for the presynaptic membrane (Hrp, blue). (A) Presence of the *nrg* Pacman wild-type construct resulted in wild-type muscle 4 NMJs. The inset shows individual presynaptic boutons at higher magnification. (B) The Nrg180^Y-F^ mutation resulted only in small alterations of NMJ growth. (C) The Nrg180^Y-A^ mutation led to a significant increase in NMJ length. (D) Deletion of the Nrg180–FIGQY motif resulted in a significant, almost 2-fold overgrowth and a corresponding reduction in the area of individual boutons. (E and F) Quantification of bouton number, NMJ length, and bouton area. NMJ growth defects correlated with an increasing loss of Ank2 binding capacities. No alterations were observed for mutations affecting the PDZ protein binding site of Nrg180 or the Nrg167–FIGQY motif. Values were normalized to wild-type rescue. Asterisks indicate highly significant changes (*p*≤0.001) for bouton number in (E) and for bouton area in (F) (*n* = 69–176 NMJs for bouton number, *n* = 20 NMJs for NMJ length, and *n* = 10 NMJs for bouton area quantifications). Scale bar in (A) corresponds to (A–D), 10 µm, inset 5 µm. Error bars represent SEM.

In addition to the synapse stability defects, we observed a second striking defect in these animals. With an increasing reduction in Ank2 binding capacities of Nrg180 we observed an increase in growth of the NMJ as reflected by an increase in the span of the presynaptic nerve terminal and an increase in the number of synaptic boutons (Figure 5A–E). At the same time we observed a corresponding decrease in synaptic bouton area (Figure 5F). Interestingly, only subtle alterations were observed for the Nrg180 Y-F mutation that still binds Ank2 efficiently (Figure 5B,E,F). The identical phenotypes of the FIGQY deletion and of the C-terminal deletion indicate that control of NMJ growth critically depends on the Nrg180-FIGQY motif (Figure 5E,F). Similar to the analysis of synapse stability we did not observe any phenotypes in *nrg^14^*; P[*nrg167^ΔFIGQY^*] or *nrg^14^*; P[*nrg180^ΔPDZ^*] mutant animals (Figure 5E,F and Figure S5D). Finally, we tested whether we could mimic these growth defects by ectopically expressing mutant Nrg180 (UAS-*nrg180^ΔFIGQY^-GFP*) in wild-type animals. High levels of expression of Nrg180^ΔFIGQY^-GFP but not of Nrg180-GFP resulted in an almost 2-fold increase in bouton number and in the span of the presynaptic nerve terminal (Figure S8A–D). Together, our data demonstrate that the loss of Ank2 binding capacities of Nrg180 correlates with both a loss of NMJ growth control and an impairment of synapse stability, suggesting that these two parameters are tightly coupled.

### The Nrg180 FIGQY Motif Is Essential for Giant Fiber Synapse Development

To address synaptic functions of the Nrg180–Ank2 interaction in the central nervous system (CNS), we extended our analysis to the adult Giant Fiber (GF) circuitry. We used the GF to TTMn (Tergo-trochanteral motoneuron) connection as a model neuro-neuronal synapse as it provides precise genetic control of pre- and postsynaptic neurons [54]. Previous analysis of *nrg* mutations affecting either homophilic cell adhesion properties (*nrg^849^*) or protein levels (*nrg^305^*) identified both axon guidance and synaptic defects at the GF terminal [55],[56]. Our Pacman-based mutants enabled us to directly determine potential requirements of the intracellular regulation of the Nrg–Ankyrin interaction for GF circuit formation and function.

**Figure 6:**
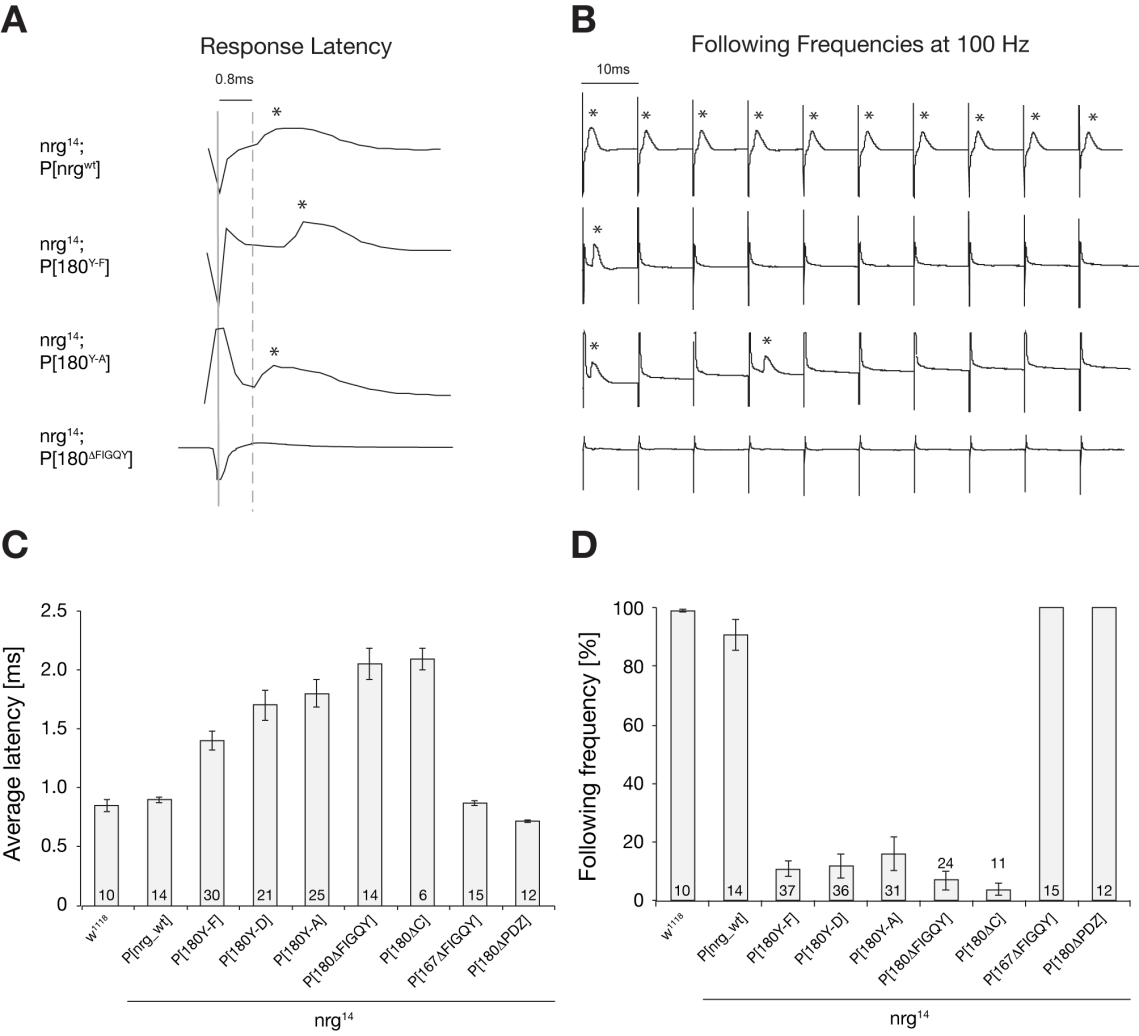
Electrophysiological phenotypes of *nrg* mutants in the giant fiber circuit. (A and B) Sample traces of different *nrg* mutants. (A) TTM responses in *nrg* mutants (asterisks) upon GF stimulation in the brain (solid grey line). The average response latency in wild-type flies is 0.8 ms (dashed grey line). Sample traces of *nrg^14^*; P[*nrg^wt^*], *nrg^14^*; P[*nrg180^Y-F^*], *nrg^14^*; P[*nrg180^Y-A^*], and *nrg^14^*; P[*nrg180^ΔFIGQY^*] are shown. Mutations in the Nrg180-FIGQY motif led to a delay or absence of responses at the TTM. (B) As a measure for synaptic reliability, the ability to follow stimuli at 100 Hz was determined. In contrast to *nrg^14^*; P[*nrg^wt^*], the GF–TTM pathway in *nrg^14^*; P[*nrg180^Y-F^*], *nrg^14^*; P[*nrg180^Y-A^*], and *nrg^14^*; P[*nrg180^ΔFIGQY^*] mutants was not able to follow stimuli at 100 Hz upon GF stimulation in the brain; only rare responses were observed (asterisks). (C and D) Quantifications of electrophysiological phenotypes of *nrg* mutants. (C) Average latency of wild-type and *nrg* mutants. There was no significant difference (*p* = 0.681, Mann–Whitney Rank Sum Test) in the average response latency between control (*w^1118^*) and *nrg^14^*; P[*nrg^wt^*], *nrg^14^*; P[*nrg167^ΔFIGQY^*], or *nrg^14^*; P[*nrg180^ΔPDZ^*] flies. In contrast, the response latency was significantly increased in all *nrg180* mutants with a mutated FIGQY motif (Mann–Whitney Rank sum test, *p*≤0.001). (D) Average following frequencies at 100 Hz in wild-type and *nrg* mutants. There was no significant difference (*p* = 0.841, Mann–Whitney Rank Sum Test) in the average of following frequencies at 100 Hz between control flies (*w^1118^*) and *nrg* ^14^; P[*nrg^wt^*], *nrg* ^14^; P[*nrg167^ΔFIGQY^*], and *nrg* ^14^; P[*nrg180^ΔFIGQY^*]. In contrast, following frequencies were significantly reduced in all *nrg180* mutants with a missense mutation in or deletion of the FIGQY motif (Mann–Whitney Rank sum test, *p*≤0.001). Error bars represent SEM.

We analyzed the function of the GF to TTM (Tergo-Trochanteral Muscle) pathway in all viable *nrg^14^*; P[*nrg*-mutant] animals by intracellular recordings from the TTM using either brain or thoracic stimulation to differentiate between potential GF-TTMn synapse or TTMn NMJ defects. Importantly, presence of the wild-type *nrg* construct in *nrg* null mutants (*nrg^14^*; P[*nrg*_wt]) established normal function of the GF-TTMn circuit. We observed no significant differences in average response latencies or following frequencies after a train of stimulations at 100 Hz when compared to wild-type control animals (Figure 6). In contrast, all mutations affecting the Nrg180 FIGQY motif caused equally severe impairments of GF circuit function. The average response latency, a measure for synaptic strength, was significantly increased (Figure 6), and mutant animals were not able to follow trains of high-frequency stimulations; in some animals we observed a complete absence of responses (Figure 6B,D). In contrast, when we bypassed the GF and stimulated the motoneurons directly using thoracic stimulation, both response latency and ability to follow high-frequency stimulation were normal in all tested animals (unpublished data). This indicates that the observed defects were specific to the GF-TTMn synaptic connection. Similarly, we observed a disruption of synapse function when expressing UAS-Nrg180^ΔFIGQY^-GFP simultaneously pre- and postsynaptically at the GF synapse in wild-type animals (average latency increased to 1.01±0.057 ms; following frequency reduced to 52.34±7.17%), further demonstrating the importance of the Nrg180 FIGQY motif for normal GF synapse development. In contrast, neither the Nrg167 FIGQY nor the C-terminal Nrg180 PDZ protein-binding domains were essential for GF circuit function (Figure 6C,D).

**Figure 7:**
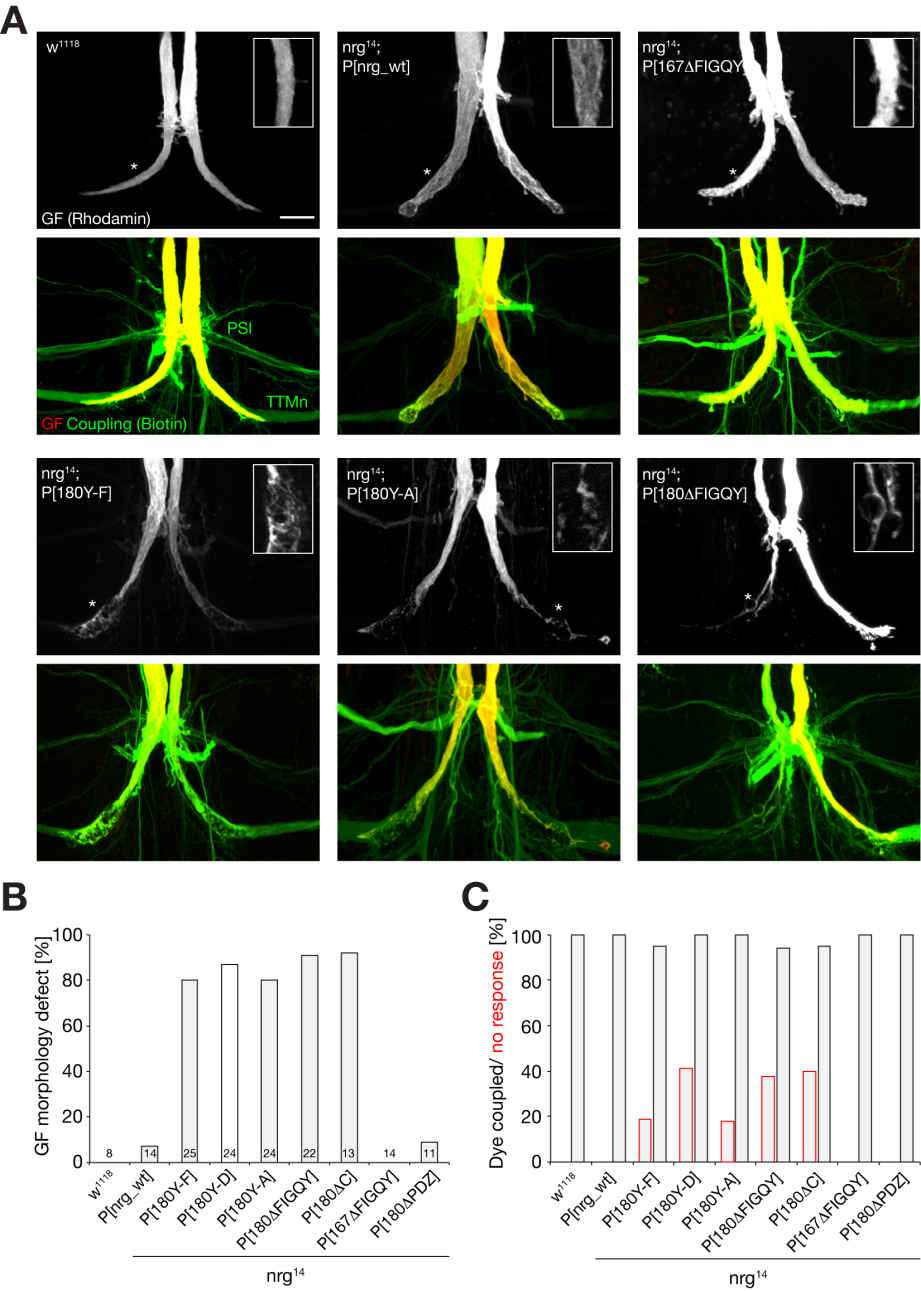
Anatomical phenotypes of the giant fiber synaptic terminals in *nrg* mutants. (A) GF synaptic terminals were visualized by injection of Rhodamine-dextran (red) into the GF. Dye-coupling of the GF to its target neurons, the Tergo Trochanteral motoneuron (TTMn), and the peripheral synapsing interneuron (PSI) via co-injection of Biotin (green) allows the detection of gap junctions between these neurons. In *w^1118^*, *nrg* ^14^; P[*nrg^wt^*] and *nrg* ^14^; P[*nrg167^ΔFIGQY^*], a normal, large GF terminal was present and we observed dye-coupling with the TTMn and the PSI. In *nrg* ^14^; P[*nrg180^Y-F^*], *nrg* ^14^; P[*nrg180^Y-A^*], and *nrg* ^14^; P[*nrg180^ΔFIGQY^*] mutants, the presynaptic terminal of the GF exhibited variable abnormal morphologies. They were thinner or swollen and contained large vacuole-like structures. However, in most cases the GF still dye-coupled with the postsynaptic target, the TTMn, and the PSI. Scale bar, 15 µm. (B) Quantification of morphological defects in *w^1118^* flies and *nrg* mutants. Only mutations affecting the Nrg180–FIGQY motif resulted in severe GF terminal aberrations. (C) Quantification of GF-to-TTMn dye-coupling (black bars) and comparison to animals with no electrophysiological responses (red bars) of the TTM with GF stimulation in the brain. A large percentage of animals expressing mutant versions of Nrg180–FIGQY proteins completely lacked electrophysiological responses despite the presence of dye-coupling, demonstrating a severe functional defect in these animals.

In order to identify potential morphological phenotypes and to distinguish between axon guidance and synaptic defects, we co-injected large (Rhodamin-dextran) and small (Biotin) fluorescent dyes into the GF. In wild-type animals the large dye is confined to the GF and reveals the morphology of the synaptic terminal. In wild-type animals the GF-TTMn synapse grows to a large presynaptic terminal with mixed electrical and chemical synapses [54]. Biotin can pass through gap-junctions and thereby dye-couple pre- and postsynaptic neurons in animals with a synaptic connection. While we observed no obvious morphological alterations of GF terminals in *nrg^14^*; P[*nrg*_wt], *nrg^14^*; P[*nrg167^ΔFIGQY^*], or *nrg^14^*; P[*nrg180^ΔPDZ^*] mutant flies, all mutations affecting the Nrg180 FIGQY motif resulted in severely disrupted GF terminals (Figure 7A,B). The GFs were present within the synaptic target area, however large areas of the synaptic terminals were either thinner or swollen and often contained large vacuole-like structures (Figure 7A, insets). Similar to the electrophysiological phenotypes, we observed no obvious qualitative or quantitative differences between different Nrg180 FIGQY mutations. Next, we directly tested for the presence of a synaptic connection between the GF and the postsynaptic TTMn using the dye-coupling assay. We found a residual synaptic connection in more than 90% of animals carrying mutations in the Nrg180 FIGQY motif (Figure 7A,C). However, dye-coupling was often weaker or required longer injection times in mutant animals when compared to animals rescued with the wild-type construct. These results indicate that at most GF terminals of Nrg180^FIGQY^ mutants, synaptic connections with at least a small number of gap junctions were established. When we correlated the ability to dye-couple with the electrophysiological properties of these synapses, we observed that approximately 40% of Nrg180^FIGQY^ mutant animals that were positive in the dye-coupling assay did not show any functional response (Figure 7C). This suggests that the synaptic strength was below the threshold to trigger an action potential in the postsynaptic TTMn. In contrast, neither the deletion of the FIGQY motif of Nrg167 nor of the PDZ protein-binding domain of Nrg180 affected GF morphology or function (Figure 7). Thus, we conclude that a wild-type Ankyrin binding motif of Nrg180 but not of Nrg167 is essential for normal GF-TTMn synapse maturation and function, but it is not required for GF axon guidance or synapse targeting.

### Transsynaptic Coordination of Pre- and Postsynaptic Development by Nrg180

**Figure 8:**
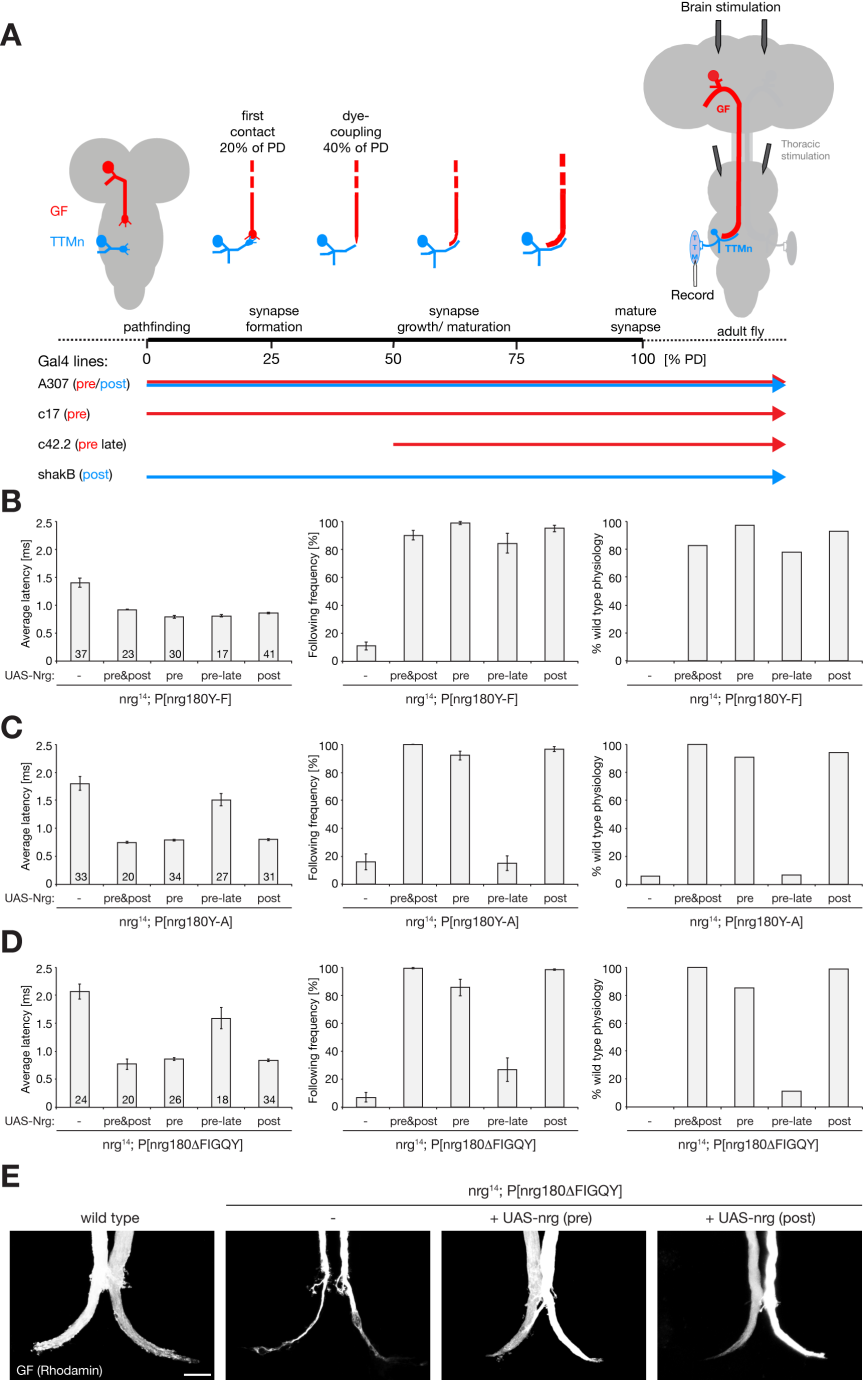
Temporal and spatial requirements of transsynaptic Nrg signaling. (A) Schematic of GF to TTMn synapse development. First dye-coupling between the GF (red) and the TTMn (blue) can be demonstrated at 40% of pupal development [54]. Positions of stimulating and recording electrodes are indicated. Brain stimulation was used to test the GF–TTMn synapse, while thoracic stimulation bypasses the GF and allowed testing of the TTMn NMJ directly. Expression profiles of the different Gal4 lines are indicated. (B) Rescue of *nrg^14^*; P[*nrg180^Y-F^*] phenotypes using Gal4/UAS-mediated expression of wild-type Nrg180. Both average response latency and the ability to follow high-frequency stimulation could be rescued significantly by simultaneous expression of Nrg180 pre- and postsynaptically or on either side of the synapse alone (Mann–Whitney Rank sum test, *p*≤0.001). Less than 20% of animals showed an electrophysiological impairment even when a late presynaptic Gal4 driver line was used for rescue (right). (C and D) Rescue of *nrg^14^*; P[*nrg180^Y-A^*] and *nrg^14^*; P[*nrg180^ΔFIGQY^*] animals using cell autonomous expression of Nrg180. Simultaneous expression of Nrg180 pre- and postsynaptically or only on one side of the synapse throughout development significantly rescued the response latency (left) and following frequencies (middle) of these mutations. More than 80% of all GF–TTMn synapses showed wild-type properties (right, Mann–Whitney Rank sum test, *p*≤0.001). In contrast to *nrg^14^*;P [*nrg180^Y-F^*], late expression of UAS–*nrg180* in the GF alone in *nrg^14^*; P[*nrg180^Y-A^*] and *nrg^14^*; P[*nrg180^ΔFIGQY^*] animals did not significantly improve the average response latency (left, Mann–Whitney Rank sum test, *p* = 0.061 and *p* = 0.057, respectively) or the following frequencies (middle, Mann–Whitney Rank sum test, *p* = 0.9 and *p* = 0.081, respectively). (E) Presynaptic GF terminal morphology was rescued by either pre- or postsynaptic expression of Nrg180 in *nrg* ^14^; P[*nrg180^ΔFIGQY^*] mutant animals. Scale bar, 15 µm. Error bars represent SEM.

The similar phenotypes of *nrg180* mutations affecting Ank2 binding either weakly (Y-F) or strongly (ΔFIGQY) indicate that normal GF synapse development requires a dynamic regulation of this interaction by phosphorylation, a feature disrupted by all mutations. Our Pacman-based mutants enabled us to determine temporal and spatial requirements of a wild-type, modifiable, Nrg180-FIGQY motif as we can express wild-type Nrg180 in the background of our mutants using the Gal4/UAS system. We used previously characterized Gal4-driver lines that allow expression of Nrg180 either simultaneously in pre- and postsynaptic neurons of the GF-TTMn synapse, only in one of the two partner neurons, or only during late stages of synaptic development in the GF (Figure 8A) [57]. Simultaneous expression of wild-type Nrg180 in pre- and postsynaptic neurons throughout GF circuit development was able to rescue all electrophysiological and morphological defects associated with the Nrg180-FIGQY mutations (using Y-F, Y-A, and Nrg180ΔFIGQY as representative examples) (Figure 8B–D and unpublished data). Thus, this assay is suitable to determine specific pre- or postsynaptic requirements of the Nrg–Ank2 interaction. To our surprise, we were able to rescue the anatomical and physiological phenotypes to a similar extent by expressing wild-type *nrg180* either in the pre- or the postsynaptic neuron in the background of the Pacman-based mutants (Figure 8). We did not observe any nonresponding animals, the average response latency was significantly restored, and only subtle and rare defects in the ability to follow multiple stimuli at 100 Hz were evident (Figure 8B–D). Furthermore, pre- or postsynaptic expression was also sufficient to rescue the morphological phenotypes of the GF synapse terminal of *nrg^14^*; P[*nrg180^ΔFIGQY^*] mutant animals (Figure 8E).

Finally, we tested whether there might be different temporal requirements of Nrg180 wild-type expression during GF synapse development. For this, we used a Gal4 line that drives presynaptic expression only after the initial connection between the GF and the TTMn has been established (Figure 8A) [54],[57]. Strikingly, this late expression of wild-type Nrg180 was not sufficient to rescue *nrg^14^*; P[*nrg180^ΔFIGQY^*] or *nrg^14^*; P[*nrg180^Y-A^*] mutant animals but efficiently restored electrophysiological properties in *nrg^14^*; P[*nrg180^Y-F^*] mutants (Figure 8B–D).

## Discussion

Precise control of synaptic connectivity is essential for the formation, function, and maintenance of neuronal circuits. Here we identified the L1-type CAM Neuroglian as a key regulator of synapse stability in vivo. By combining biochemical, biophysical, and genetic assays at two complementary model synapses, we demonstrate that regulation of the Nrg180–Ankyrin2 interaction plays a critical role in controlling synapse growth, maturation, and maintenance. Several important findings arise from our work: (1) Control of synapse stability requires Nrg-mediated cell adhesion, which can be controlled by direct coupling to the presynaptic Ankyrin-associated cytoskeleton. (2) Synapse elimination and axonal retraction display striking phenotypic similarities to developmentally controlled synapse elimination at the vertebrate NMJ, suggesting common cellular mechanisms between developmental and disease processes. (3) Local regulation of the Nrg180–Ankyrin2 interaction provides a mechanism to gradually control the delicate balance between synapse growth and stability. (4) Transsynaptic Nrg signaling mediates the coordination of pre- and postsynaptic development in the CNS and requires a dynamic regulation of the Ankyrin-motif both temporally and spatially. (5) The FIGQY motif enables synapse-specific regulation of L1-type proteins to differentially control connectivity and function in distinct neuronal circuits.

### The L1-Type CAM Neuroglian Controls Synapse Stability

A large number of cell adhesion molecules have been implicated as important mediators of synapse development, but the regulatory mechanisms controlling structural synapse plasticity and maintenance remain largely unknown. In an unbiased RNAi screen, we identified the *Drosophila* L1-type CAM Neuroglian as essential for synapse stability at the neuromuscular junction. We demonstrate that knockdown of presynaptic Nrg induces synapse disassembly that shares all cellular hallmarks of synapse retractions observed in *ank2*, *spec*, or *hts* mutant animals [26]–[29]. By analyzing individual motoneurons lacking any Nrg expression, we verified this presynaptic requirement of Nrg for synapse maintenance. In addition, this allowed us to unravel the cellular events occurring in response to loss of cell adhesion at the presynaptic nerve terminal. In *nrg* mutant motoneurons, we observed both synaptic retractions and motoneuron axons ending in “retraction bulb”–like structures. Excitingly, we directly observed eliminated NMJs that were still connected via traces of clonally marked presynaptic membrane remnants to retraction bulb–like structures. This demonstrates that loss of synapse stability can induce a cellular program resulting in the retraction of the motoneuron axon accompanied by shedding of presynaptic membrane. This phenotype shares striking similarities with developmental synapse elimination at the vertebrate NMJ [58] and points to similar cellular programs underlying synapse loss in development and disease. It will be of particular interest to analyze the contribution of glial cells in this process as they are part of a pro-degenerative signaling system at the NMJ and actively clear membrane remnants of degenerating or pruning axons in both *Drosophila* and vertebrates [58]–[61]. It is important to note that some of the axonal phenotypes would also be consistent with a stalling of the axonal growth cone before reaching the appropriate target. Indeed, prior studies in both *Drosophila* and vertebrates demonstrated a function of Nrg and L1CAM during neurite outgrowth [14],[16],[47]–[49], indicating that both defects in axon growth and loss of synapse stability may have contributed to the observed phenotypes.

Although Nrg is critical for NMJ maintenance, our observation that 50% of larval NMJs were still stable in *nrg* null mutants indicates that redundant mechanisms control synapse stability at the level of synaptic cell adhesion molecules. A candidate to provide such redundancy would be the *Drosophila* NCAM homolog FasII, which has been previously implicated in NMJ maintenance [62] and can substitute for Nrg during axonal outgrowth of ocellar neurons [48]. However, we demonstrate that FasII cannot compensate for the loss of presynaptic Nrg at the larval NMJ (Figure 1G and Figure S1D). The identification of the entire combinatorial code of CAMs contributing to synapse stability will be of high interest in the future.

### The Nrg–Ank2 Interaction Functions as a Molecular Switch to Balance Synapse Growth and Stability

The dynamic nature of many neuronal circuits requires controlled changes in synapse assembly and disassembly without a disruption of neuronal circuit function. While interactions of synaptic cell adhesion molecules are essential to maintain synaptic connectivity, mechanistic insights regarding the regulation of these interactions to alter transsynaptic adhesion are limited to date. The process is probably best understood for Cadherins where adhesive properties are modulated either via binding of extracellular calcium or by altering their association with intracellular Catenins via posttranslational phosphorylation [7],[8]. These changes alter localization, clustering, and transsynaptic signaling of Cadherins leading to modulations of synaptic connectivity and function [9],[10]. Here we identify the interaction between the L1-type CAM Nrg and the adaptor protein Ank2 as a similar control module. First, we demonstrate that Nrg180 directly interacts with Ank2 in vivo. Second, a series of specific mutations in the Ankyrin binding motif allowed us to differentially modulate the Ankyrin-binding capacity of Nrg180. We demonstrate that decreasing Ank2-binding capacities correlate with an up to 2-fold increase in lateral mobility of Nrg180 in motoneurons. This is consistent with studies in vertebrates demonstrating that phosphorylation of the conserved tyrosine of the FIGQY motif reduces or abolishes binding to Ankyrins and increases mobility of L1-type CAMs [20]–[23],[52]. Finally, Pacman-based *nrg* mutants with altered Ankyrin-binding capacity caused two striking phenotypes. There was a significant increase in synapse retractions in mutants with severely impaired Ank2 binding but not in mutants with partial binding (Nrg180^Y-F^). In addition, we observed increased NMJ growth that correlated in a similar manner with the decrease in Ank2 binding capacities. The reduction in Ank2 binding potentially decreases the static population and adhesive force mediated by Nrg and thereby impairs synapse stability. At the same time this reduction in transsynaptic adhesion might allow for increased NMJ growth. We previously identified similar switch-like alterations of synapse growth and stability in animals lacking the spectrin-binding and actin-capping protein Hts/Adducin [28]. Importantly, studies of *adducin2* mutant mice demonstrated that Adducin2 provides a similar function in vertebrates and is essential to mediate changes in synaptic connectivity relevant for learning and memory [63],[64].

Interestingly, we did not observe significant alterations in presynaptic Nrg180 or Ank2 levels in these animals similar to previous observations for the axonal localization of these proteins [50],[65]. However, we found a clear dependence on the respective partner protein at semi-stable *nrg* and *ank2* mutant synapses, indicating that the Nrg–Ank2 interaction is required to maintain their synaptic localization. A similar late loss of AnkyrinG has been observed in *neurofascin* mutant Purkinje cells, demonstrating a function of the L1-CAM paralog for maintenance but not for initial localization of AnkG to the AIS [33],[35] and likewise AnkG is required for the maintenance of Neurofascin [32]. Together these data indicate that modulation of the Nrg–Ank2 interaction balances synapse growth and stability. Changing the interaction via posttranslational phosphorylation could thus locally decrease synapse stability, thereby allowing the formation of new synapses without impairing general neuronal circuit architecture.

### Transsynaptic Nrg Function Requires Dynamic Regulation of the Ankyrin Binding Motif

Despite our detailed knowledge regarding the expression of synaptic cell adhesion molecules, mechanistic insights into the transsynaptic control of synapse maturation or function are only recently emerging [1],[2],[9],[10]. Here we provide evidence that transsynaptic coordination of synapse development can be controlled via a dynamic regulation of the L1-type CAM Nrg.

In contrast to the larval NMJ, the lack of significant differences in phenotypic strength between the mutations in Nrg180-FIGQY motif demonstrates that normal GF synapse development requires a dynamic regulation of the Nrg–Ank2 interaction via phosphorylation. To address the importance of this regulation for transsynaptic development, we selectively reintroduced wild-type Nrg180 either pre- or postsynaptically in the background of the different FIGQY motif mutations. Surprisingly, pre- or postsynaptic expression of wild-type Nrg in the presence of mutant Nrg on both sides of the synapse was sufficient to restore synaptic function in all mutants, but late presynaptic expression could only rescue the Nrg180^Y-F^ mutation. This highlights two important novel aspects of Nrg function at central synapses. First, while Nrg180 is required on both sides of the synapse, regulation of the FIGQY motif is sufficient on either side of the synapse, demonstrating that Nrg can control synapse development in a transsynaptic manner. Second, constitutive binding to Ank2 is sufficient for early stages of synapse development, but GF synapse maturation requires dynamic regulation of the Nrg–Ank2 interaction. A potential function of the phosphorylation of Nrg could be an increase of lateral mobility of Nrg to allow precise spatial alignment with postsynaptic CAMs. Alternatively, phosphorylation may enable an interaction with proteins that bind only phosphorylated Nrg. One candidate would be the microtubule binding protein Doublecortin that binds only phosphorylated Neurofascin [66], but physiological relevance for this interaction in nervous system development is lacking to date.

While we observe distinct functions and modes of regulation of Nrg at peripheral versus central synapses in both cases, the Nrg180–Ank2 interaction did not influence axon outgrowth or guidance. In addition, we did not observe any requirements for the Nrg167 FIGQY motif or for the PDZ-binding motif of Nrg180, which has recently been implicated in controlling axonal outgrowth in *Drosophila* mushroom bodies [53].

A surprising observation from studies of vertebrate L1 family proteins was that mutations within the intracellular domain that are linked to human L1/CRASH syndrome and neuropathological diseases [30] resulted in significantly weaker phenotypes in mice compared to the complete L1 knockout [14],[31],[67],[68]. While extracellular interactions are essential for early nervous system development including neurite outgrowth and axon targeting [16], here we provide evidence that reversible phosphorylation of the intracellular Ankyrin binding motif might provide a regulatory module to fine tune synaptic connectivity without impairing overall circuit stability. The expansion of the L1-type CAM family to four independent proteins in vertebrates may provide the means to cope with the diversity and complexity of synaptic connectivity in the vertebrate CNS. Indeed, while mutations in the L1CAM Ankyrin motif did not affect the general organization of the nervous system, they resulted in specific impairments of particular neuronal circuits and at subsets of synapses [31],[67],[68]. The functions of the different L1-type proteins may be distinct, partly opposing or redundant as evident by an analysis of cerebellar granule cell development in L1CAM and NrCAM double mutants [36]. Our data suggest that the coordinated phosphorylation of a subpopulation of synaptic L1 family proteins may allow differential modulation of biophysical properties of L1 complexes to precisely control distinct aspects of synapse development. Elucidating the synaptic L1-family protein code at specific synapses and identifying their phosphorylation status during synapse development and in response to activity might uncover new mechanisms controlling synaptic plasticity in development and during learning and memory.

## Materials and Methods

### Fly Stocks

Flies were maintained at 25°C on standard food. Crosses and most experiments were performed at 25°C, while RNAi assays were performed at 27°C. The following fly strains have been used in this study: *w^1118^* (wild-type), *nrg^14^* (*nrg^1^*), *nrg^17^* (*nrg^2^*), UAS–*mCD8*–*GFP*, UAS–*fasII*, *elav^C155^*–Gal4, *ok371*–Gal4, *sca*–Gal4, *mef2*–Gal4, BG57–Gal4, UAS–*dcr2*, *ok307*–Gal4 (A307–Gal4), P(hsFLP)86E, P(hsFLP)1, P(neoFRT)19A (all Bloomington stock center), c17-Gal4, c42.2–Gal4, and *shakB*–Gal4 [57]. RNAi lines were obtained from the Vienna *Drosophila* RNAi Center [37]: Nrg RNAi line^1^ (stock ID6688) and Nrg RNAi line^2^ (stock ID107991).

### Generation of Neuroglian UAS and P[acman] Constructs

The full-length Nrg180 ORF was amplified from the plasmid pMT–Neuroglian and the Nrg167 ORF from cDNA GH03573 (both obtained from the *Drosophila* Genomic Research Center, Indiana, USA). Full-length ORFs were cloned into pENTR vector via TOPO cloning (Invitrogen). To obtain pUASTattB-10xUAS destination vectors suited for gateway cloning, a gateway cassette with a C-terminal 3xHA or EGFP tag was introduced into the pWALIUM10-moe plasmid (TRiP collection, Harvard Medical School). Final expression constructs were generated via gateway cloning using standard procedures (Invitrogen). Deletions and point mutations were introduced into pENTR clones using the QuickChange II site-directed mutagenesis kit following the manufacturer's instructions (Agilent Technologies). All constructs were verified by sequencing (FMI sequencing facility). The P[acman] clone CH321-4H20 was obtained from BACPAC Resources Center (BPRC, Oakland, California) and modified using *galK*-mediated recombineering [44] according to [42] (NCI Frederick National Laboratory). Site-specific integration via the phi-C31 system [43] was used to generate insertions at the attP40-landing site for both pUAST and Pacman constructs. Primers used in this study are listed in Table S3.

### Immunohistochemistry and Antibody Production

Wandering third instar larvae were dissected in standard dissecting saline and fixed with Bouin's fixative for 2–3 min (Sigma-Aldrich). Primary antibodies were incubated at 4°C overnight. Primary antibodies were used at the following dilutions: anti-Nrg180 (BP104) 1∶250 [38], anti-Bruchpilot (nc82) 1∶250, anti-Futsch (22c10) 1∶500, anti-Synapsin (3c11) 1∶100 (all obtained from Developmental Studies Hybridoma Bank, IA), rabbit anti-Dlg 1∶30,000, rabbit anti-DGluRIII [28] 1∶2,500, rabbit anti-DvGlut, rat anti-CD8 (Caltag Laboratories) 1∶1,000, anti-Nrg (3c1, gift from M. Hortsch, Ann Arbor, MI, USA [38]) 1∶500, rabbit anti-Nrg^FIGQY^ (raised against the peptide: TEDGSFIGQYVPGKLQP) 1∶100, and rabbit anti-Nrg180^cyto^ (raised against the peptide: NNSAAAHQAAPTAGGGSGAA) 1∶500. Monoclonal rat anti-Ank2-L 1∶40 was generated against a protein fragment containing aa 3134–3728 (according to the 4,083 aa isoform of Ank2-L). Rabbit anti-Ank1-4 antibody was generated against the Ankyrin domains 1–4. This antibody recognizes both Ankyrin1 and Ankyrin2. Antibodies were generated at David's Biotechnology (Regensburg, Germany).

Alexa conjugated secondary antibodies (Invitrogen) were used at 1∶1,000 for 2 h at RT. Directly conjugated anti-Hrp (Alexa or Cy-dyes) were used at 1∶100–1,000 (Jackson Immunoresearch Laboratories). Larval preparations were mounted in Prolong Gold (Invitrogen). Images were captured at room temperature using a Leica SPE confocal microscope. To process, analyze images, and quantify phenotypes Adobe Photoshop, Imaris (Bitplane), Image Access (Imagic), and the open source tool FIJI/ImageJ were used.

### Quantification of Phenotypes

Synaptic retractions were quantified using presynaptic Brp and postsynaptic DGluRIII staining and counting the number of unopposed postsynaptic footprints. Complete loss of the presynaptic marker Brp was considered an elimination of the presynaptic nerve terminal. Synapse retraction frequencies are presented as values per animal. NMJs on the indicated muscles in segments A2–A5 (10 NMJs/animal) were scored. *n* indicates the number of independent animals per quantification.

Bouton area, number, and NMJ length were quantified using Synapsin, Dlg, and Hrp staining. Bouton area and NMJ length were quantified using the Image access software (Imagic). Hrp staining was used to visualize the bouton area and 10 A3 muscle 4 NMJs were quantified per genotype. To measure NMJ length 20 muscle 4 NMJs (segments A3 and A4, 10 each) were analyzed. Bouton number was quantified on muscle 4 in segments A2–A6 using Synapsin/Dlg staining.

### Western Blot and Immunoprecipitation

Larval brains were dissected and transferred into 2× sample buffer (Invitrogen). Five brains per lane were analyzed on NuPage gels (Invitrogen) according to standard procedures. Primary antibodies were incubated overnight at 4°C. Secondary Hrp-conjugated goat anti-mouse and goat anti-rabbit antibodies were used at 1∶10,000 (Jackson Immunoresearch) for 2 h at RT. PVDF-membranes were incubated with ECL substrate (SuperSignal West Pico Kit, Thermo Scientific) and developed on film (Fujifilm).

For immunoprecipitations (IPs) 100 larval brains were collected, grinded in NP40-based lysis buffer, and incubated on ice for 30 min. The supernatant was split equally between control IPs using empty protein-G beads (Dynabeads, Life Science) and protein-G beads pre-incubated with Nrg180^BP104^ antibody. IPs were analyzed with anti-Nrg180^BP104^ (1∶200) and rat anti-Ank2-L (1∶20). For IPs of mutated Nrg180 proteins, S2 cells were co-transfected with act5C–Gal4, UAS–Ank2-S–EGFP [27], and UAS–Nrg–3xHA plasmids using Fugene (Roche) following the manufacturer's instructions. IPs were analyzed using mouse anti-HA (12CA5) 1∶200 and rabbit anti-GFP (Molecular Probes) 1∶500 antibodies. Rabbit anti-Ank2 (anti–Ank1–4) 1∶1,000 was used for visualization of the input. Quantification of Ank2 binding between Nrg180 mutants was performed using four independent IP experiments and Odyssey2.1 software (LI-COR).

### Fluorescence Recovery after Photobleaching (FRAP)

Wandering third instar larvae expressing *nrg*–*GFP* using *ok371*–Gal4 were dissected in HL3 saline and prepared for live imaging using a magnetic pinholder device. 1-Naphthylacetyl-spermin-trihydrochloride (NSH) (100 mM; Sigma, St Louis, MO) was added to the HL3 saline to block postsynaptic glutamate receptor activation and muscle contractions. Six to nine motoneuron axons from three to four independent animals were analyzed. Motoneuron axons were photo-bleached using a Zeiss LSM700 by scanning the targeted region for 30 iterations at 100% laser-power using the 488 nm line. Ten images were acquired before the bleach and 40 after the bleach with a time interval of 5 s at low laser power.

Images of the FRAP series were corrected for animal movement using the FIJI registration plugin (StackReg option). Images were corrected by substracting background fluorescence from regions outside the axons and corrected for bleaching using a control area within the same axon.

The recovery curves were fit to a double exponential curve as follows:
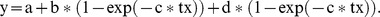



The maximum was calculated from the fitting curve (max^fitting^). To calculate the real max value, the following formula has been used:
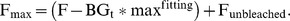



The mobile fraction was calculated using the following formula:
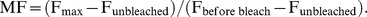



### MARCM Analysis

The *nrg* null mutation *nrg^14^* was recombined with the P(neoFRT)19A chromosome. The indicated Pacman constructs were crossed into this background to create stable stocks. These lines were crossed to P(hsFLP)1, P(neoFRT)19A, tubGal80; *ok371*-Gal4, UAS–CD8–GFP; MKRS, P(hsFLP)86E flies. Embryos were collected for 2 h, aged for 3 h, and heat shocked for 1 h at 37°C.

### Statistical Analysis

All statistical analyses were performed using Microsoft Office Excel and an online source for unpaired Student's *t* test (http://www.physics.csbsju.edu/stats/t-test.html). *p*≤0.05 was accepted as statistically significant (*p≤0.05, **p≤0.01, ***p≤0.001).

### Giant Fiber Preparation

Adult *Drosophila* nervous system was dissected, dye filled, and fixed as previously described [55]. Young 2- to 5-d-old flies were used for all the experiments. To visualize the morphology of GF–TTMn connection either a 10 mM Alexa Fluor 568 Hydrazide (Molecular Probes) in 200 mM KCl or a dye solution of 10% w/v Neurobiotin (Vector labs) and tetramethyl rhodamine-labeled dextran (Invitrogen) in 2 M potassium acetate was injected into the GF axons by passing hyperpolarizing or depolarizing current, respectively. Preparation of GF samples for confocal microscopy has been described previously [55]. Samples were analyzed using a Nikon C1si Fast Spectral Confocal system. Images were processed using Nikon Elements Advance Research 4.0 software.

### Electrophysiology

Electrophysiological recordings from the giant fiber circuit were obtained as described in detail in [69]. The flies were given 10 single pulses at 30–60 mV for 0.03 ms with a 5-s interval between the stimuli and the shortest response latency of each fly was averaged. To determine the reliability of the circuit, the ability to follow frequencies at 100 Hz was determined. For this 10 trains of 10 stimuli were given at 100 Hz with an interval of 2 s between the trains and percent of the total responses was calculated. All traces were recorded, stored, and analyzed using pClamp 10 (Molecular Devices) software. Mann–Whitney Rank sum test was used to determine significant differences between different genotypes in average response latencies and following frequencies (Sigma Plot 11 software).

## Supporting Information

Figure S1 Presynaptic Nrg is essential for synapse stability. (A–C) NMJs on muscle 6/7 stained for the presynaptic motoneuron membrane (Hrp, white), the presynaptic active zone marker Brp (green), and postsynaptic glutamate receptors (DGluRIII, red). (A) A stable wild-type NMJ indicated by perfect apposition of pre- and postsynaptic markers. (B) Knockdown of presynaptic Nrg resulted in severe synaptic retractions indicated by a fragmented presynaptic membrane and the loss of presynaptic Brp despite the presence of postsynaptic glutamate receptors. The example shows a complete elimination of an entire NMJ at muscle 6/7. Please note the characteristic increase in postsynaptic glutamate receptor clusters at sites of retractions (inset). (C) Loss of muscle Nrg did not impair synapse stability. Scale bar in (A) corresponds to (A–C), 10 µm, inset 5 µm. (D) Quantification of different *nrg* RNAi conditions. Neuronal- but not muscle-specific knockdown of Nrg using different Gal4 driver combinations or independent RNAi constructs resulted in a significant increase in synaptic retractions on muscle 6/7. The retraction frequency was significantly rescued (*p*≤0.001) by co-expression of UAS–*nrg180* but not by co-expression of either UAS–*mCD8*–*GFP* or UAS–*fasII*. Expression of UAS–*fasII* alone did not result in a significant increase in retractions (genotypes: neu^1^ = *elav^C155^*–Gal4; neu^2^ = *elav^C155^*–Gal4; *ok371*–Gal4; neu^3^ = *elav^C155^*–Gal4; UAS-*dcr2*; neu^4^ = *elav^C155^*–Gal4; *sca*–Gal4 UAS–*dcr2*; mus^1^ = UAS–*dcr2*; *mef2*–Gal4; RNAi^1^ = V6668; RNAi^2^ = V107991; rescue indicates co-expression of the listed UAS construct; the number of analyzed animals is indicated). (E) Quantification of retraction severity on muscle 6/7. Only neuronal knockdown of Nrg resulted in a significant increase in the severity of synapse retractions. (F) Quantification of retraction severity on muscle 4. Only neuronal knockdown of Nrg resulted in a significant increase in the severity of synapse retractions. A large fraction of observed synaptic retractions represent complete presynaptic eliminations. Error bars represent SEM.
(TIF)

Figure S2 Analysis of pre- and postsynaptic Nrg localization after RNAi-mediated knockdown. (A–D) Muscle 4 NMJs stained with an antibody specific to the cytoplasmic tail of Nrg180 (Nrg180^BP104^, white and green) and the presynaptic membrane (Hrp, red). (A) In wild-type animals Nrg180 was present in the motoneuron axon and within the presynaptic nerve terminal marked by the membrane marker. In contrast to the uniform distribution in the axon, Nrg was present in a punctate pattern at the terminal and co-localized with Hrp at the ends of small filopodia-like membrane extensions. (B) Neuronally expressed *nrg* RNAi resulted in an almost complete knockdown of Nrg180 in the presynaptic motoneuron. (C) Muscle-specific knockdown of Nrg altered the normal distribution of Nrg180 in the presynaptic nerve terminal. (D) Co-expression of Nrg180 with *nrg* RNAi resulted in a complete rescue of Nrg180 levels and distribution at the NMJ. (E–G) Muscle 4 NMJs stained with an antibody recognizing both Nrg isoforms (Nrg167/180^3c1^, white and green) and the presynaptic membrane marker Hrp (red). (E) In addition to neuronally expressed Nrg180, we observed Nrg167 throughout the postsynaptic muscle and in glial cells surrounding the motoneuron axon. (F) Muscle-specific knockdown efficiently eliminated Nrg167 expression in the muscle. Presynaptic Nrg can still be detected (asterisk). A tracheal branch expressing Nrg167 is indicated (t). (G) Neuronal-specific knockdown significantly reduced Nrg expression in the motoneuron and the presynaptic nerve terminal (arrows). Nrg167 can still be observed in glial cells surrounding the motoneurons and in the postsynaptic SSR. (H) Analysis of Nrg expression in motoneurons that are enwrapped by glial cells. In wild-type Nrg180 expression (Nrg180^cyto^) is confined to neurons marked by Hrp (blue). Surrounding glial cells express high levels of Nrg167. Knockdown of neuronal Nrg abolishes all Nrg staining in the nerve (asterisk) but does not affect glial expression. Scale bar in (A) corresponds to (A–G), 10 µm, insets 5 µm.
(TIF)

Figure S3 Analysis of *nrg* MARCM clones. (A) A *nrg^14^* MARCM clone rescued by a wild-type *nrg* Pacman construct. The motoneuron clone was marked by the expression of mCD8–GFP (green). Synaptic vesicles (DvGlut, red) were found opposite postsynaptic Dlg (blue), indicating a stable NMJ (insets). Neighboring NMJs are visible that were not mutant, as evident by the absence of the clonal marker. (B) A *nrg^14^* MARCM clone rescued by a Pacman construct lacking the FIGQY motif of Nrg167. No alterations in NMJ stability or organization were observed. (C) A *nrg^14^* MARCM clone expressing a mutated form of Nrg180 lacking the FIGQY motif. A “bulb-like” structure (arrow) was present in close proximity to an NMJ that contained postsynaptic profiles marked by Dlg but no presynaptic vesicles (asterisk). In contrast to the neighboring wild-type NMJ, postsynaptic Dlg staining was reduced and no longer formed a continuous structure (inset). While no membrane marker remnants were visible at the eliminated NMJ, we observed small GFP-puncta in between the NMJ and the retracted axon (arrowhead). (D) Axonal area of a *nrg^14^* MARCM clone rescued by a wild-type *nrg* Pacman construct. Within the axon, only very low levels of the synaptic vesicle marker DvGlut were evident. (E) A “bulb-like” structure in a *nrg^14^* MARCM clone. The axon ended in a large swelling that contained increased levels of the active zone marker Brp. (F) Axonal area of a *nrg^14^* MARCM clone rescued by a Pacman construct lacking the FIGQY motif of Nrg167. No alterations of axonal membrane or the synaptic vesicle marker DvGlut were evident. (G) A “bulb-like” structure in a *nrg^14^* MARCM clone expressing P[*nrg180^ΔFIGQY^*]. The axon ended in a large swelling that showed an aberrant accumulation of the synaptic vesicle marker DvGlut. (H) Analysis of the innervation pattern of stable NMJs of MARCM clones of indicated genotypes. In all cases we observed similar muscle innervation rates. Scale bar in (A) corresponds to (A-G), 10 µm, insets 5 µm.
(TIF)

Figure S4 Analysis of the expression of genomic Nrg Pacman rescue constructs. (A) Nrg180 expression of different Pacman introduced *nrg* mutations in the background of the *nrg* null mutation *nrg^14^*. All *nrg* Pacman constructs were expressed at wild-type levels at the NMJ. The cytoplasmic domain-specific antibody Nrg180^cyto^ (green) detected Nrg180 at the NMJ in all mutant animals. In contrast, the Nrg180–FIGQY-specific antibody Nrg180^BP104^ (white) did not recognize Nrg180 carrying mutations in the FIGQY motif. (B) Western blot analysis of larval brain extracts of all Nrg Pacman constructs in the background of the *nrg* null mutation *nrg^14^*. Nrg^3c1^ recognizes a common motif of both Nrg isoforms. Both isoforms are present in all Pacman rescued flies; Nrg^FIGQY^ specifically recognizes the FIGQY motif of both Nrg isoforms; thus, the mutated forms were not detected in the Western blot. Nrg180^BP104^ recognizes the FIGQY motif of Nrg180. No signal could be detected in animals expressing mutated versions of the FIGQY motif of Nrg180. (C) Muscle 4 NMJs stained for both Nrg isoforms (Nrg^3c1^, green, white). P[*nrg^wt^*] rescued Nrg expression and distribution of both Nrg isoforms in motoneurons, glial cells, and muscles of *nrg^14^* mutant animals. (D) Western blot analysis to assay the expression of Nrg lacking Ig3–4 domains. As P[*nrg^ΔIg3–4^*] did not rescue the embryonic lethality associated with the *nrg^14^* mutation, we tested if normal levels of mutated Nrg isoforms were expressed in a wild-type background. Using isoform-specific antibodies, we could visualize equal expression levels of truncated proteins of both isoforms. (E) Muscle 4 NMJs stained for the Nrg180^cyto^ antibody that recognizes Nrg180 C-terminal to the FIGQY motif. In wild-type the antibody was present in a pattern similar to Nrg180^BP104^, showing a punctate pattern within the presynaptic nerve terminal (see also A). No specific signal could be detected in *nrg^14^* mutant animals rescued by P[*nrg180^ΔC^*], demonstrating the specificity of the antibody and the specificity of the introduced mutation. Scale bar in (A), 5 µm; (C) and (E), 10 µm.
(TIF)

Figure S5 The FIGQY motif of Nrg167 is not required for NMJ development and stability. (A and B) Analysis of synapse stability in *nrg^14^* mutant animals rescued either by a wild-type *nrg* Pacman construct or by a construct lacking the FIGQY motif of Nrg167. No differences in NMJ stability were observed. (C and D) Analysis of NMJ growth and morphology in *nrg^14^* mutant animals rescued either by a wild-type *nrg* Pacman construct or by a construct lacking the FIGQY motif of Nrg167. No differences in NMJ development were observed. Scale bar in (A) corresponds to (A–D), 5 µm, inset 5 µm.
(TIF)

Figure S6 Postsynaptic Nrg contributes to NMJ stability. (A) Postsynaptic knockdown of Nrg in nrg*^14^* mutant animals rescued by a wild-type *nrg* Pacman construct did not cause synaptic retractions. (B) Postsynaptic knockdown of Nrg in *nrg^14^* mutant animals rescued by a Pacman construct carrying a deletion of the FIGQY motif of Nrg180 showed prominent synaptic retractions. (C and D) Quantification of synaptic retraction frequency and severity demonstrates a significant increase in synaptic retractions when postsynaptic Nrg is knocked down in the *nrg^14^* mutant animals rescued by Nrg180^ΔFIGQY^ but not in animals rescued by the wild-type Pacman construct. Scale bar in (A) corresponds to (A-B), 5 µm. Error bars represent SEM.
(TIF)

Figure S7 Ank2 mutations affect presynaptic localization of Nrg180. (A) Analysis of Ank2-L levels and distribution in *nrg^14^* mutant animals rescued by different *nrg* Pacman constructs. We did not observe obvious changes in Ank2-L localization or levels at stable synapses in different Pacman rescued *nrg* mutants. (B–D) NMJs on muscle 4 stained for Nrg180 (Nrg180^BP104^, green, white) and the presynaptic membrane (Hrp, red). (B) In wild-type animals, Nrg180 was present throughout the presynaptic nerve terminal co-localizing with the membrane marker Hrp. (C and D) Examples of *ank2* mutant NMJs. At semistable synapses that still have intact presynaptic membranes (as judged by continuous Hrp staining), we observed a partial or complete loss of Nrg180. In addition, Nrg180 levels in the axon were severely reduced. Scale bar in (A), 5 µm; (B), 10 µm.
(TIF)

Figure S8 Dominant-negative functions of Nrg180 lacking the FIGQY motif. (A) Expression of wild-type Nrg180–GFP in motoneurons did not alter NMJ development. (B) Expression of Nrg180–ΔFIGQY–GFP in motoneurons resulted in significant overgrowth of the NMJ. Scale bar in (A) corresponds to (A) and (B), 10 µm. (C) Quantification of bouton number. (D) Quantification of NMJ length. Error bars represent SEM.
(TIF)

Table S1 List of 287 RNAi lines targeting potential cell adhesion molecules.
(DOCX)

Table S2 Original data displayed in Figures 1, 2, 4, and 5 and Figures S6 and S8.
(DOCX)

Table S3 List of primers.
(DOCX)

## Abbreviations

Ank2: Ankyrin2
Brp: Bruchpilot
CAM: cell adhesion molecule
GFP: green fluorescent protein
Ig: Immunoglobin
MARCM: mosaic analysis with a repressible cell marker
NCAM: neuronal cell adhesion molecule
NMJ: neuromuscular junction
Nrg: Neuroglian
RNAi: RNA interference
