# Supplementary figures and images for: Transsynaptic Coordination of Synaptic Growth, Function, and Stability by the L1-Type CAM Neuroglian
Source: PLoS Biol. 2013 Apr 16;11(4):e1001537. doi: 10.1371/journal.pbio.1001537 (PMC3627646; doi:10.1371/journal.pbio.1001537)

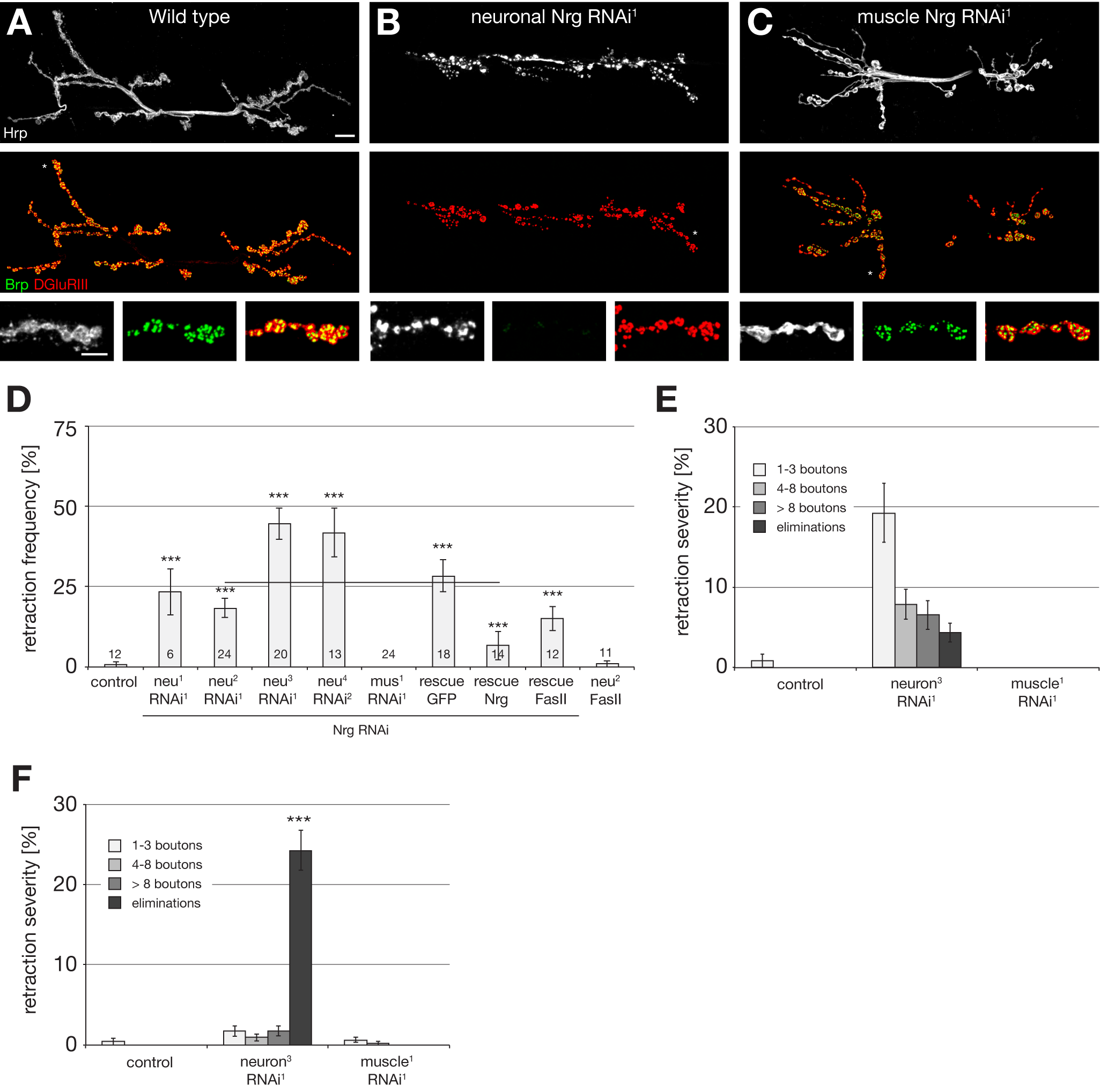

Supplement: Figure S1 — Presynaptic Nrg is essential for synapse stability. (A–C) NMJs on muscle 6/7 stained for the presynaptic motoneuron membrane (Hrp, white), the presynaptic active zone marker Brp (green), and postsynaptic glutamate receptors (DGluRIII, red). (A) A stable wild-type NMJ indicated by perfect apposition of pre- and postsynaptic markers. (B) Knockdown of presynaptic Nrg resulted in severe synaptic retractions indicated by a fragmented presynaptic membrane and the loss of presynaptic Brp despite the presence of postsynaptic glutamate receptors. The example shows a complete elimination of an entire NMJ at muscle 6/7. Please note the characteristic increase in postsynaptic glutamate receptor clusters at sites of retractions (inset). (C) Loss of muscle Nrg did not impair synapse stability. Scale bar in (A) corresponds to (A–C), 10 µm, inset 5 µm. (D) Quantification of different nrg RNAi conditions. Neuronal- but not muscle-specific knockdown of Nrg using different Gal4 driver combinations or independent RNAi constructs resulted in a significant increase in synaptic retractions on muscle 6/7. The retraction frequency was significantly rescued (p≤0.001) by co-expression of UAS–nrg180 but not by co-expression of either UAS–mCD8–GFP or UAS–fasII. Expression of UAS–fasII alone did not result in a significant increase in retractions (genotypes: neu1 = elavC155–Gal4; neu2 = elavC155–Gal4; ok371–Gal4; neu3 = elavC155–Gal4; UAS-dcr2; neu4 = elavC155–Gal4; sca–Gal4 UAS–dcr2; mus1 = UAS–dcr2; mef2–Gal4; RNAi1 = V6668; RNAi2 = V107991; rescue indicates co-expression of the listed UAS construct; the number of analyzed animals is indicated). (E) Quantification of retraction severity on muscle 6/7. Only neuronal knockdown of Nrg resulted in a significant increase in the severity of synapse retractions. (F) Quantification of retraction severity on muscle 4. Only neuronal knockdown of Nrg resulted in a significant increase in the severity of synapse retractions. A large fraction of observed s [file pbio.1001537.s001.tif]

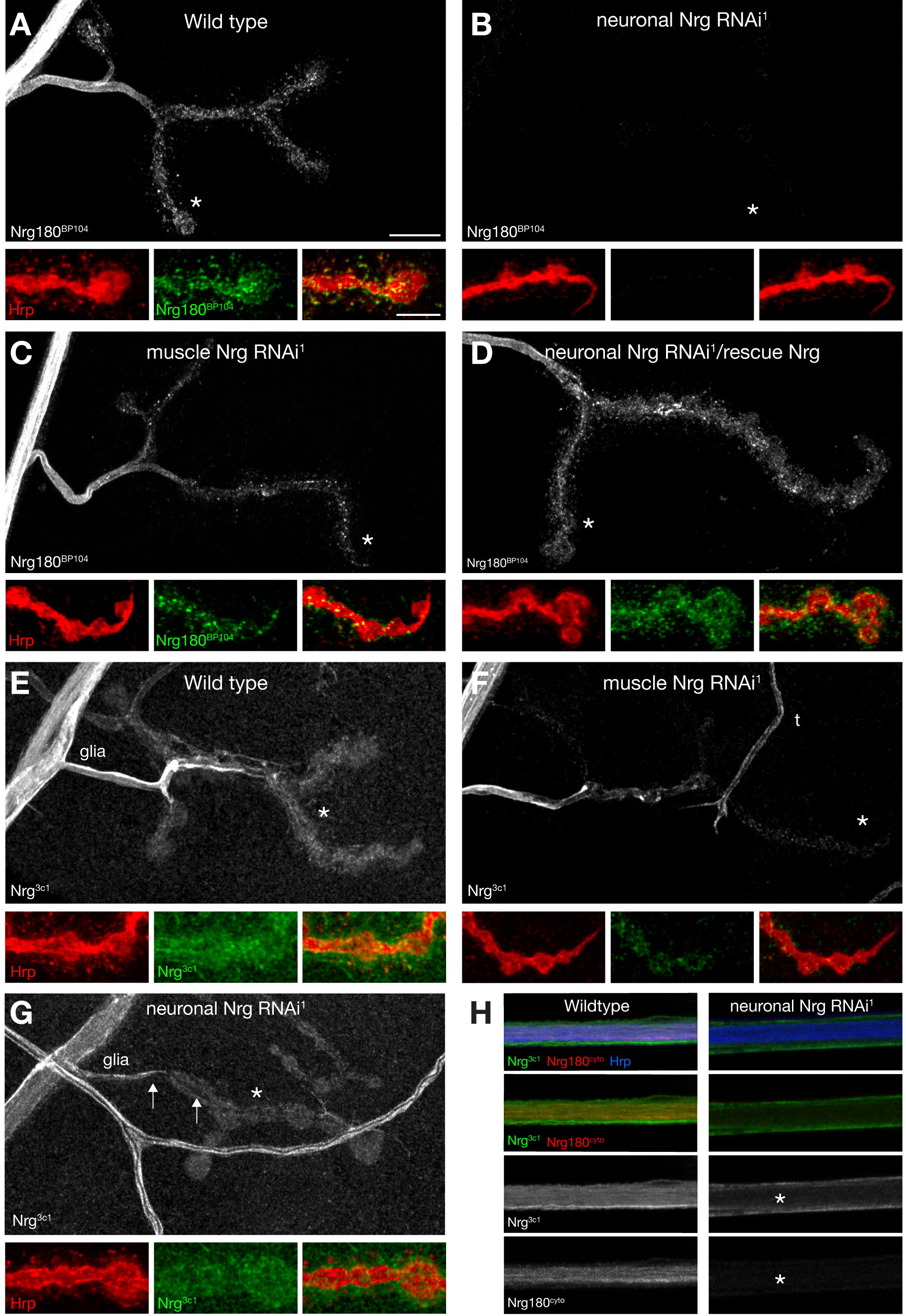

Supplement: Figure S2 — Analysis of pre- and postsynaptic Nrg localization after RNAi-mediated knockdown. (A–D) Muscle 4 NMJs stained with an antibody specific to the cytoplasmic tail of Nrg180 (Nrg180BP104, white and green) and the presynaptic membrane (Hrp, red). (A) In wild-type animals Nrg180 was present in the motoneuron axon and within the presynaptic nerve terminal marked by the membrane marker. In contrast to the uniform distribution in the axon, Nrg was present in a punctate pattern at the terminal and co-localized with Hrp at the ends of small filopodia-like membrane extensions. (B) Neuronally expressed nrg RNAi resulted in an almost complete knockdown of Nrg180 in the presynaptic motoneuron. (C) Muscle-specific knockdown of Nrg altered the normal distribution of Nrg180 in the presynaptic nerve terminal. (D) Co-expression of Nrg180 with nrg RNAi resulted in a complete rescue of Nrg180 levels and distribution at the NMJ. (E–G) Muscle 4 NMJs stained with an antibody recognizing both Nrg isoforms (Nrg167/1803c1, white and green) and the presynaptic membrane marker Hrp (red). (E) In addition to neuronally expressed Nrg180, we observed Nrg167 throughout the postsynaptic muscle and in glial cells surrounding the motoneuron axon. (F) Muscle-specific knockdown efficiently eliminated Nrg167 expression in the muscle. Presynaptic Nrg can still be detected (asterisk). A tracheal branch expressing Nrg167 is indicated (t). (G) Neuronal-specific knockdown significantly reduced Nrg expression in the motoneuron and the presynaptic nerve terminal (arrows). Nrg167 can still be observed in glial cells surrounding the motoneurons and in the postsynaptic SSR. (H) Analysis of Nrg expression in motoneurons that are enwrapped by glial cells. In wild-type Nrg180 expression (Nrg180cyto) is confined to neurons marked by Hrp (blue). Surrounding glial cells express high levels of Nrg167. Knockdown of neuronal Nrg abolishes all Nrg staining in the nerve (asterisk) but does not affect glial expression. Scale ba [file pbio.1001537.s002.tif]

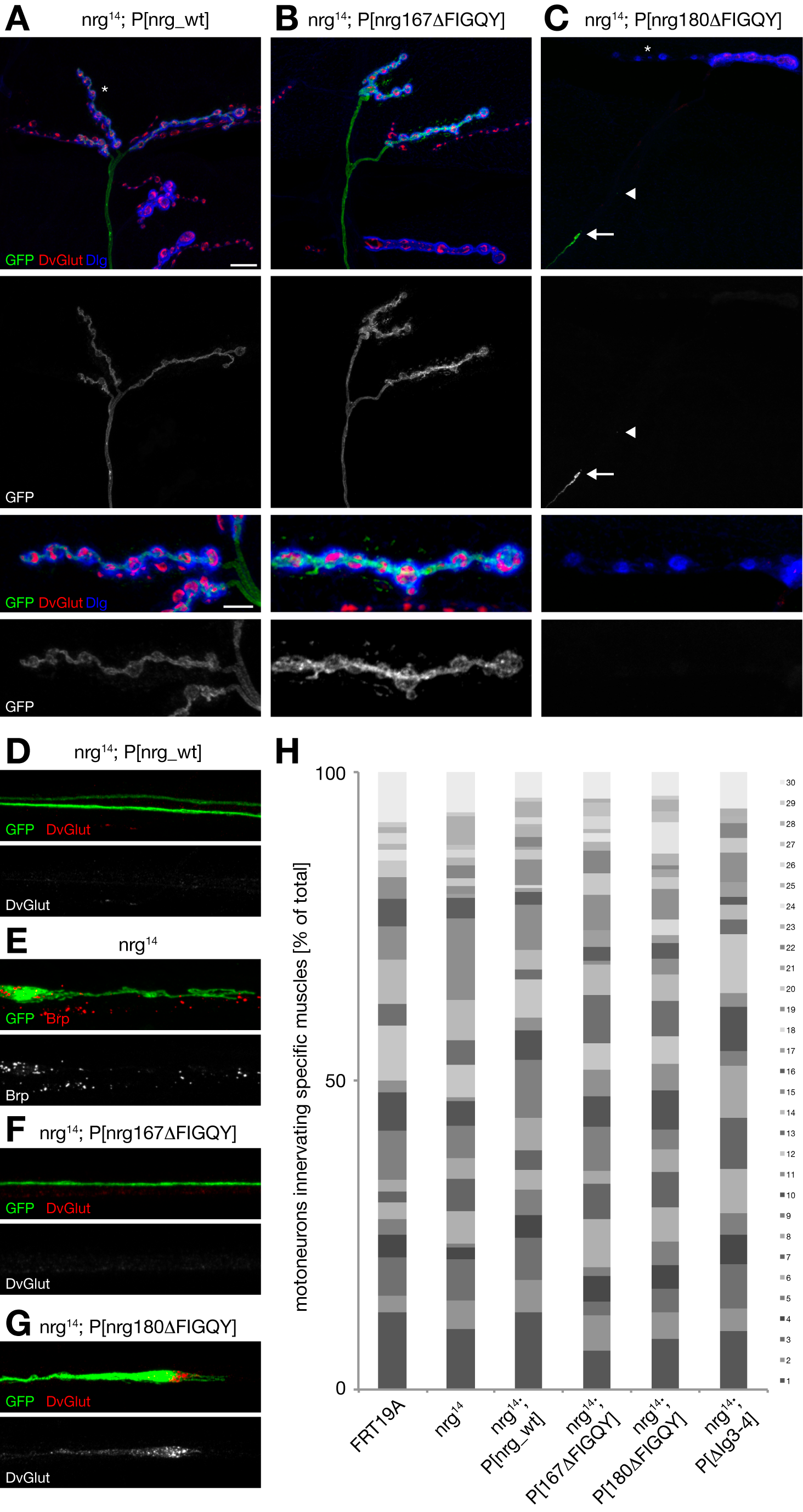

Supplement: Figure S3 — Analysis of nrg MARCM clones. (A) A nrg14 MARCM clone rescued by a wild-type nrg Pacman construct. The motoneuron clone was marked by the expression of mCD8–GFP (green). Synaptic vesicles (DvGlut, red) were found opposite postsynaptic Dlg (blue), indicating a stable NMJ (insets). Neighboring NMJs are visible that were not mutant, as evident by the absence of the clonal marker. (B) A nrg14 MARCM clone rescued by a Pacman construct lacking the FIGQY motif of Nrg167. No alterations in NMJ stability or organization were observed. (C) A nrg14 MARCM clone expressing a mutated form of Nrg180 lacking the FIGQY motif. A “bulb-like” structure (arrow) was present in close proximity to an NMJ that contained postsynaptic profiles marked by Dlg but no presynaptic vesicles (asterisk). In contrast to the neighboring wild-type NMJ, postsynaptic Dlg staining was reduced and no longer formed a continuous structure (inset). While no membrane marker remnants were visible at the eliminated NMJ, we observed small GFP-puncta in between the NMJ and the retracted axon (arrowhead). (D) Axonal area of a nrg14 MARCM clone rescued by a wild-type nrg Pacman construct. Within the axon, only very low levels of the synaptic vesicle marker DvGlut were evident. (E) A “bulb-like” structure in a nrg14 MARCM clone. The axon ended in a large swelling that contained increased levels of the active zone marker Brp. (F) Axonal area of a nrg14 MARCM clone rescued by a Pacman construct lacking the FIGQY motif of Nrg167. No alterations of axonal membrane or the synaptic vesicle marker DvGlut were evident. (G) A “bulb-like” structure in a nrg14 MARCM clone expressing P[nrg180ΔFIGQY]. The axon ended in a large swelling that showed an aberrant accumulation of the synaptic vesicle marker DvGlut. (H) Analysis of the innervation pattern of stable NMJs of MARCM clones of indicated genotypes. In all cases we observed similar muscle innervation rates. Scale bar in (A) corresponds to (A-G), 10 µm, insets 5 µm. (TIF) [file pbio.1001537.s003.tif]

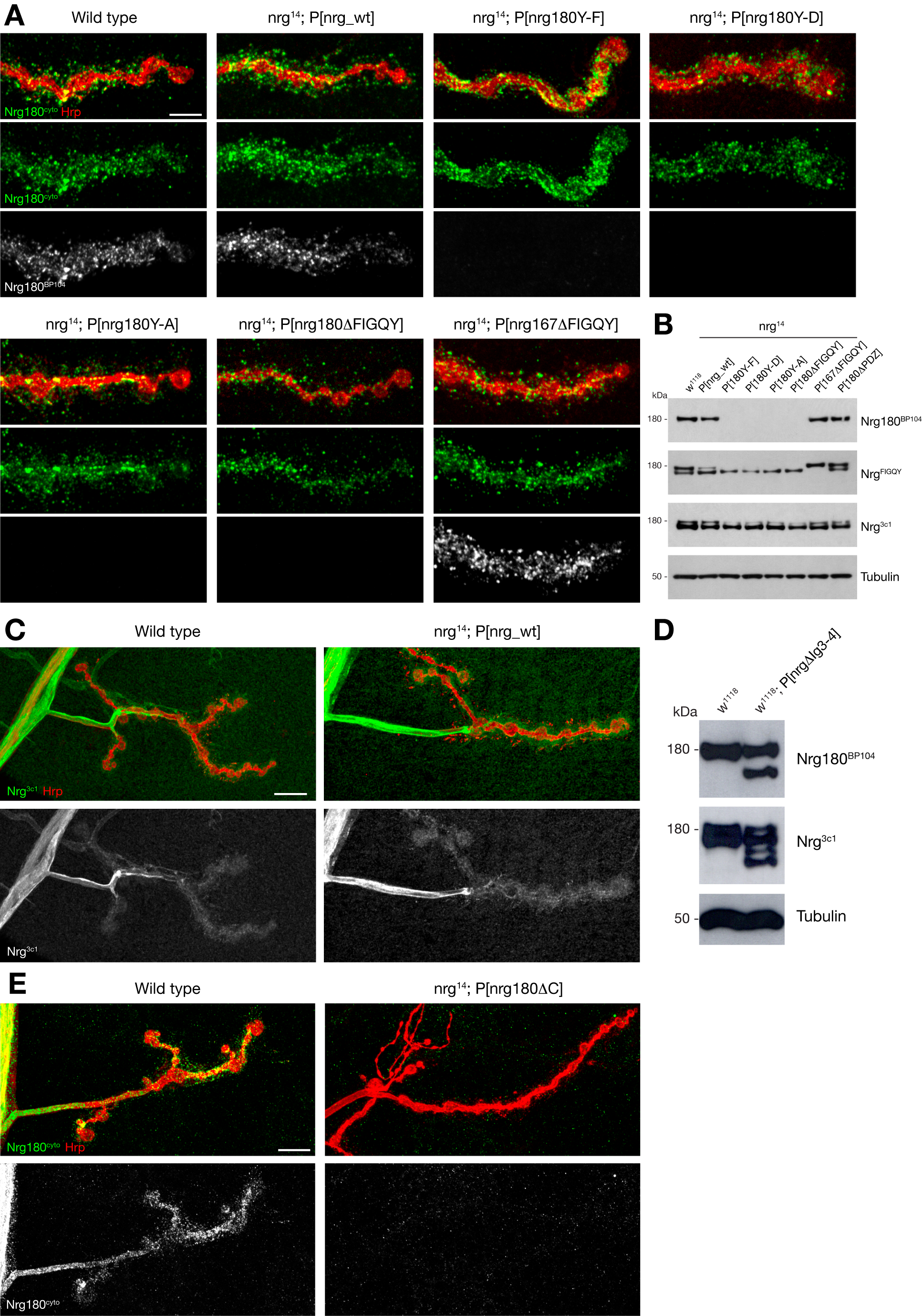

Supplement: Figure S4 — Analysis of the expression of genomic Nrg Pacman rescue constructs. (A) Nrg180 expression of different Pacman introduced nrg mutations in the background of the nrg null mutation nrg14. All nrg Pacman constructs were expressed at wild-type levels at the NMJ. The cytoplasmic domain-specific antibody Nrg180cyto (green) detected Nrg180 at the NMJ in all mutant animals. In contrast, the Nrg180–FIGQY-specific antibody Nrg180BP104 (white) did not recognize Nrg180 carrying mutations in the FIGQY motif. (B) Western blot analysis of larval brain extracts of all Nrg Pacman constructs in the background of the nrg null mutation nrg14. Nrg3c1 recognizes a common motif of both Nrg isoforms. Both isoforms are present in all Pacman rescued flies; NrgFIGQY specifically recognizes the FIGQY motif of both Nrg isoforms; thus, the mutated forms were not detected in the Western blot. Nrg180BP104 recognizes the FIGQY motif of Nrg180. No signal could be detected in animals expressing mutated versions of the FIGQY motif of Nrg180. (C) Muscle 4 NMJs stained for both Nrg isoforms (Nrg3c1, green, white). P[nrgwt] rescued Nrg expression and distribution of both Nrg isoforms in motoneurons, glial cells, and muscles of nrg14 mutant animals. (D) Western blot analysis to assay the expression of Nrg lacking Ig3–4 domains. As P[nrgΔIg3–4] did not rescue the embryonic lethality associated with the nrg14 mutation, we tested if normal levels of mutated Nrg isoforms were expressed in a wild-type background. Using isoform-specific antibodies, we could visualize equal expression levels of truncated proteins of both isoforms. (E) Muscle 4 NMJs stained for the Nrg180cyto antibody that recognizes Nrg180 C-terminal to the FIGQY motif. In wild-type the antibody was present in a pattern similar to Nrg180BP104, showing a punctate pattern within the presynaptic nerve terminal (see also A). No specific signal could be detected in nrg14 mutant animals rescued by P[nrg180ΔC], demonstrating the specificity of the antib [file pbio.1001537.s004.tif]

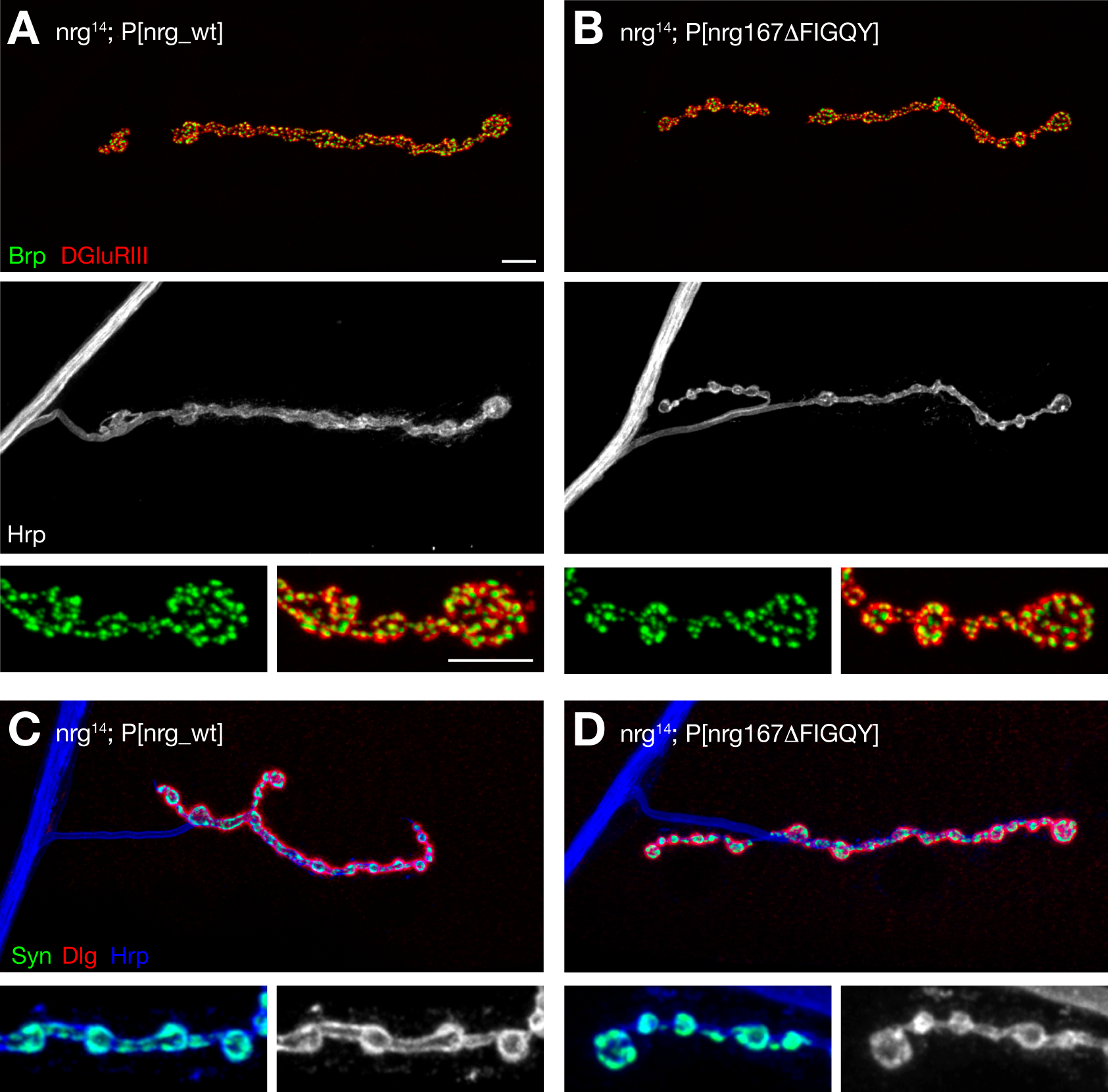

Supplement: Figure S5 — The FIGQY motif of Nrg167 is not required for NMJ development and stability. (A and B) Analysis of synapse stability in nrg14 mutant animals rescued either by a wild-type nrg Pacman construct or by a construct lacking the FIGQY motif of Nrg167. No differences in NMJ stability were observed. (C and D) Analysis of NMJ growth and morphology in nrg14 mutant animals rescued either by a wild-type nrg Pacman construct or by a construct lacking the FIGQY motif of Nrg167. No differences in NMJ development were observed. Scale bar in (A) corresponds to (A–D), 5 µm, inset 5 µm. (TIF) [file pbio.1001537.s005.tif]

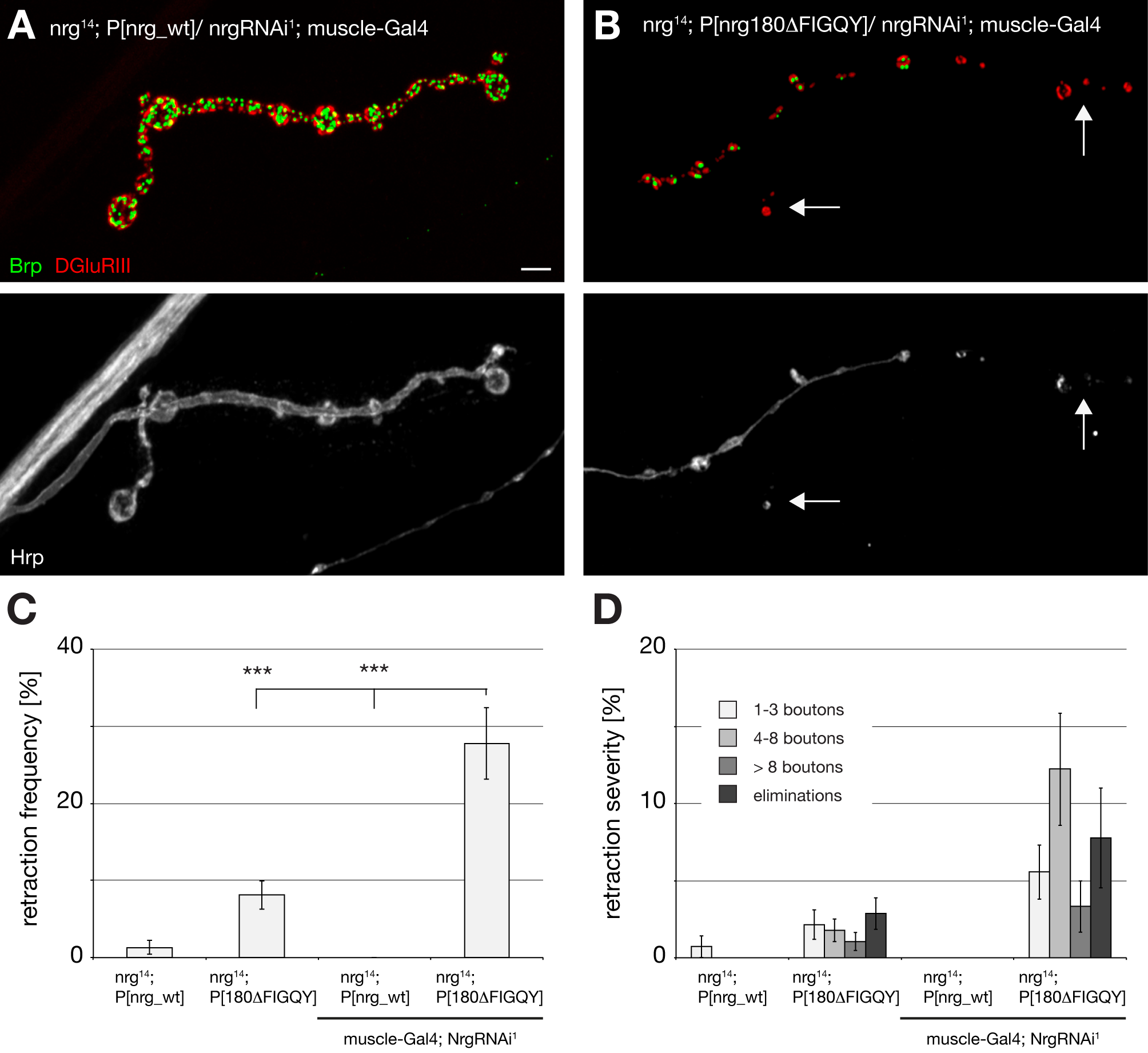

Supplement: Figure S6 — Postsynaptic Nrg contributes to NMJ stability. (A) Postsynaptic knockdown of Nrg in nrg14 mutant animals rescued by a wild-type nrg Pacman construct did not cause synaptic retractions. (B) Postsynaptic knockdown of Nrg in nrg14 mutant animals rescued by a Pacman construct carrying a deletion of the FIGQY motif of Nrg180 showed prominent synaptic retractions. (C and D) Quantification of synaptic retraction frequency and severity demonstrates a significant increase in synaptic retractions when postsynaptic Nrg is knocked down in the nrg14 mutant animals rescued by Nrg180ΔFIGQY but not in animals rescued by the wild-type Pacman construct. Scale bar in (A) corresponds to (A-B), 5 µm. Error bars represent SEM. (TIF) [file pbio.1001537.s006.tif]

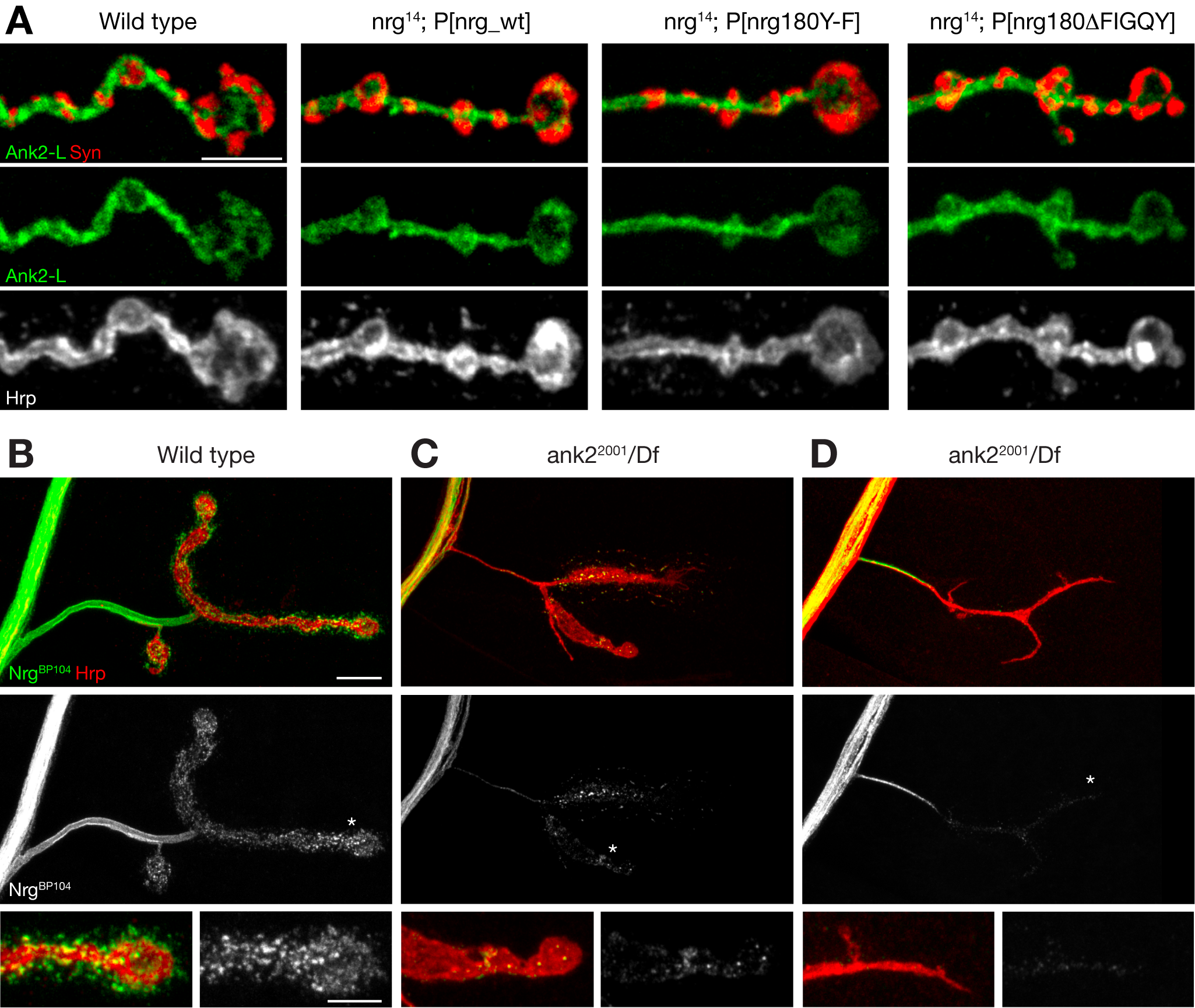

Supplement: Figure S7 — Ank2 mutations affect presynaptic localization of Nrg180. (A) Analysis of Ank2-L levels and distribution in nrg14 mutant animals rescued by different nrg Pacman constructs. We did not observe obvious changes in Ank2-L localization or levels at stable synapses in different Pacman rescued nrg mutants. (B–D) NMJs on muscle 4 stained for Nrg180 (Nrg180BP104, green, white) and the presynaptic membrane (Hrp, red). (B) In wild-type animals, Nrg180 was present throughout the presynaptic nerve terminal co-localizing with the membrane marker Hrp. (C and D) Examples of ank2 mutant NMJs. At semistable synapses that still have intact presynaptic membranes (as judged by continuous Hrp staining), we observed a partial or complete loss of Nrg180. In addition, Nrg180 levels in the axon were severely reduced. Scale bar in (A), 5 µm; (B), 10 µm. (TIF) [file pbio.1001537.s007.tif]

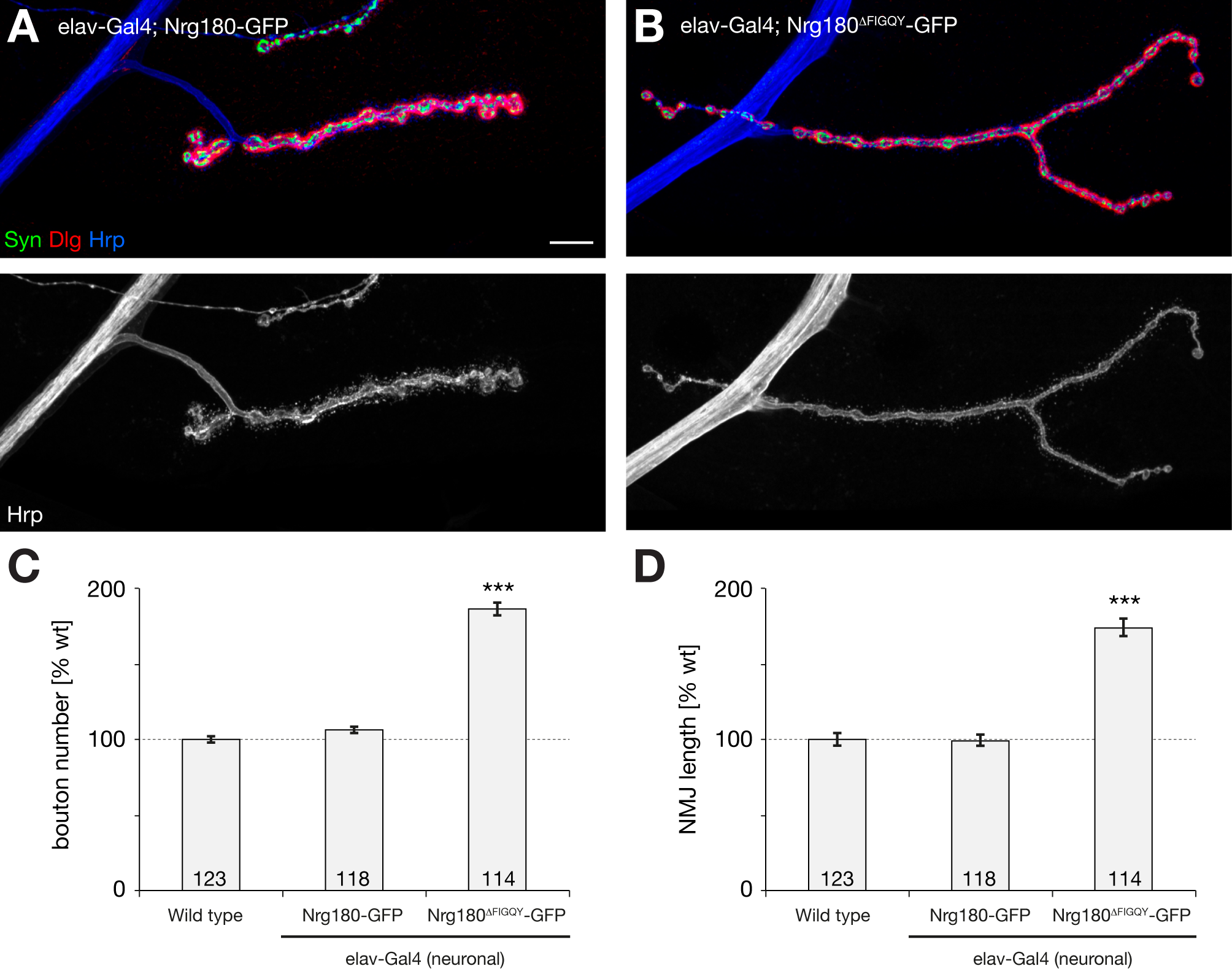

Supplement: Figure S8 — Dominant-negative functions of Nrg180 lacking the FIGQY motif. (A) Expression of wild-type Nrg180–GFP in motoneurons did not alter NMJ development. (B) Expression of Nrg180–ΔFIGQY–GFP in motoneurons resulted in significant overgrowth of the NMJ. Scale bar in (A) corresponds to (A) and (B), 10 µm. (C) Quantification of bouton number. (D) Quantification of NMJ length. Error bars represent SEM. (TIF) [file pbio.1001537.s008.tif]
