# Supplementary material for: Transsynaptic Coordination of Synaptic Growth, Function, and Stability by the L1-Type CAM Neuroglian
Source: PLoS Biol. 2013 Apr 16;11(4):e1001537. doi: 10.1371/journal.pbio.1001537 (PMC3627646; doi:10.1371/journal.pbio.1001537)
Supplement: Table S1 — List of 287 RNAi lines targeting potential cell adhesion molecules. (DOCX) [file pbio.1001537.s009.docx]

### Table S1. RNAi-lines (Vienna Drosophila RNAi Center lines)

**Ig-domain containing proteins (127)**

| ***Symbol*** | ***Name*** | ***CG number*** | ***VDRC Transformant ID*** | ***VDRC Construct ID*** |
| --- | --- | --- | --- | --- |
| ama | amalgam | CG2198 | 22944 | 12733 |
| [beat-Ia](http://flybase.bio.indiana.edu/cgi-bin/fbidq.html?FBgn0013433) | beaten path Ia | [CG4846](http://flybase.bio.indiana.edu/cgi-bin/gbrowse/dmel/?name=FBgn0013433) | 4544 | 1386 |
| [beat-Ib](http://flybase.bio.indiana.edu/cgi-bin/fbidq.html?FBgn0028645) | beaten path Ib | [CG7644](http://flybase.bio.indiana.edu/cgi-bin/gbrowse/dmel/?name=FBgn0028645) | 101662 | 105368 |
| [beat-Ic](http://flybase.bio.indiana.edu/cgi-bin/fbidq.html?FBgn0028644) | beaten path Ic | [CG4838](http://flybase.bio.indiana.edu/cgi-bin/gbrowse/dmel/?name=FBgn0028644) | 45873 | 64 |
| [beat-IIa](http://flybase.bio.indiana.edu/cgi-bin/fbidq.html?FBgn0038498) | beaten path IIa | [CG14334](http://flybase.bio.indiana.edu/cgi-bin/gbrowse/dmel/?name=FBgn0038498) | 18990 | 2587 |
| [beat-IIb](http://flybase.bio.indiana.edu/cgi-bin/fbidq.html?FBgn0038494) | beat-IIb | [CG4135](http://flybase.bio.indiana.edu/cgi-bin/gbrowse/dmel/?name=FBgn0038494) | 17815 | 6761 |
| [beat-IIIa](http://flybase.bio.indiana.edu/cgi-bin/fbidq.html?FBgn0032627) | beat-IIIa | [CG12621](http://flybase.bio.indiana.edu/cgi-bin/gbrowse/dmel/?name=FBgn0032627) | 45866 | 15037 |
| [beat-IIIb](http://flybase.bio.indiana.edu/cgi-bin/fbidq.html?FBgn0053179) | beat-IIIb | [CG33179](http://flybase.bio.indiana.edu/cgi-bin/gbrowse/dmel/?name=FBgn0053179) | 4784 | 2557 |
| [beat-IIIc](http://flybase.bio.indiana.edu/cgi-bin/fbidq.html?FBgn0032629) | beat-IIIc | [CG15138](http://flybase.bio.indiana.edu/cgi-bin/gbrowse/dmel/?name=FBgn0032629) | 27137 | 6812 |
| [beat-IV](http://flybase.bio.indiana.edu/cgi-bin/fbidq.html?FBgn0039089) | beat-IV | [CG10152](http://flybase.bio.indiana.edu/cgi-bin/gbrowse/dmel/?name=FBgn0039089) | 52413 | 16933 |
| [beat-Va](http://flybase.bio.indiana.edu/cgi-bin/fbidq.html?FBgn0038087) | beat-Va | [CG10134](http://flybase.bio.indiana.edu/cgi-bin/gbrowse/dmel/?name=FBgn0038087) | 35715 | 13423 |
| Beat-Vb | Beat-Vb | CG31298 | 17832 | 6774 |
| [beat-Vc](http://flybase.bio.indiana.edu/cgi-bin/fbidq.html?FBgn0038084) | beat-Vc | [CG14390](http://flybase.bio.indiana.edu/cgi-bin/gbrowse/dmel/?name=FBgn0038084) | 22736 | 12847 |
| [beat-VI](http://flybase.bio.indiana.edu/cgi-bin/fbidq.html?FBgn0039584) | beat-VI | [CG14064](http://flybase.bio.indiana.edu/cgi-bin/gbrowse/dmel/?name=FBgn0039584) | 27205 | 14468 |
| [boi](http://flybase.bio.indiana.edu/cgi-bin/fbidq.html?FBgn0040388) | brother of iHog | [CG32796](http://flybase.bio.indiana.edu/cgi-bin/gbrowse/dmel/?name=FBgn0040388) | 869 | 60 |
| [bt](http://flybase.bio.indiana.edu/cgi-bin/fbidq.html?FBgn0005666) | bent | [CG32019](http://flybase.bio.indiana.edu/cgi-bin/gbrowse/dmel/?dontadjust=1&name=FBgn0005666) | 46252 | 14482 |
| btl | breathless | CG32134 | 27106 | 14439 |
| [CG11320](http://flybase.bio.indiana.edu/cgi-bin/fbidq.html?FBgn0031837) | CG11320 | [CG11320](http://flybase.bio.indiana.edu/cgi-bin/gbrowse/dmel/?name=FBgn0031837) | 18054 | 7268 |
| [CG12484](http://flybase.bio.indiana.edu/cgi-bin/fbidq.html?FBgn0086604) | CG12484 | [CG12484](http://flybase.bio.indiana.edu/cgi-bin/gbrowse/dmel/?name=FBgn0086604) | 25576 | 10005 |
| [CG12950](http://flybase.bio.indiana.edu/cgi-bin/fbidq.html?FBgn0037736) | CG12950 | [CG12950](http://flybase.bio.indiana.edu/cgi-bin/gbrowse/dmel/?name=FBgn0037736) | 10011 | 2880 |
| [CG14141](http://flybase.bio.indiana.edu/cgi-bin/fbidq.html?FBgn0036146) | CG14141 | [CG14141](http://flybase.bio.indiana.edu/cgi-bin/gbrowse/dmel/?name=FBgn0036146) | 43017 | 8239 |
| [CG14372](http://flybase.bio.indiana.edu/cgi-bin/fbidq.html?FBgn0038156) | CG14372 | [CG14372](http://flybase.bio.indiana.edu/cgi-bin/gbrowse/dmel/?name=FBgn0038156) | 16636 | 5643 |
| [CG14521](http://flybase.bio.indiana.edu/cgi-bin/fbidq.html?FBgn0039617) | CG14521 | [CG14521](http://flybase.bio.indiana.edu/cgi-bin/gbrowse/dmel/?name=FBgn0039617) | 104056 | 112589 |
| [CG14964](http://flybase.bio.indiana.edu/cgi-bin/fbidq.html?FBgn0035410) | CG14964 | [CG14964](http://flybase.bio.indiana.edu/cgi-bin/gbrowse/dmel/?name=FBgn0035410) | 43603 | 8498 |
| [CG16857](http://flybase.bio.indiana.edu/cgi-bin/fbidq.html?FBgn0028482) | CG16857 | [CG16857](http://flybase.bio.indiana.edu/cgi-bin/gbrowse/dmel/?name=FBgn0028482) | 24479 | 101 |
| [CG17839](http://flybase.bio.indiana.edu/cgi-bin/fbidq.html?FBgn0036454) | CG17839 | [CG17839](http://flybase.bio.indiana.edu/cgi-bin/gbrowse/dmel/?name=FBgn0036454) | 36314 | 14436 |
| [CG31190](http://flybase.bio.indiana.edu/cgi-bin/fbidq.html?FBgn0051190) | CG31190 | [CG31190](http://flybase.bio.indiana.edu/cgi-bin/gbrowse/dmel/?name=FBgn0051190) | 6685 | 74 |
| [CG31431](http://flybase.bio.indiana.edu/cgi-bin/fbidq.html?FBgn0051431) | CG31431 | [CG31431](http://flybase.bio.indiana.edu/cgi-bin/gbrowse/dmel/?name=FBgn0051431) | 1128 | 93 |
| [CG31646](http://flybase.bio.indiana.edu/cgi-bin/fbidq.html?FBgn0051646) | CG31646 | [CG31646](http://flybase.bio.indiana.edu/cgi-bin/gbrowse/dmel/?name=FBgn0051646) | 100781 | 108592 |
| [CG31708](http://flybase.bio.indiana.edu/cgi-bin/fbidq.html?FBgn0051708) | CG31708 | [CG31708](http://flybase.bio.indiana.edu/cgi-bin/gbrowse/dmel/?name=FBgn0051708) | 38261 | 6597 |
| [CG31714](http://flybase.bio.indiana.edu/cgi-bin/fbidq.html?FBgn0032180) | CG31714 | [CG31714](http://flybase.bio.indiana.edu/cgi-bin/gbrowse/dmel/?name=FBgn0032180) | 7654 | 1052 |
| CG32387 |  |  | 1100 | 111 |
| [CG33515](http://flybase.bio.indiana.edu/cgi-bin/fbidq.html?FBgn0053515) | CG33515 | [CG33515](http://flybase.bio.indiana.edu/cgi-bin/gbrowse/dmel/?name=FBgn0053515) | 30093 | 14652 |
| [CG33543](http://flybase.bio.indiana.edu/cgi-bin/fbidq.html?FBgn0053543) | CG33543 | [CG33543](http://flybase.bio.indiana.edu/cgi-bin/gbrowse/dmel/?name=FBgn0053543) | 17859 | 67 |
| [CG34371](http://flybase.bio.indiana.edu/cgi-bin/fbidq.html?FBgn0085400) | - | [CG34371](http://flybase.bio.indiana.edu/cgi-bin/gbrowse/dmel/?name=FBgn0085400) | 44997 | 2175 |
| [CG3624](http://flybase.bio.indiana.edu/cgi-bin/fbidq.html?FBgn0034724) | CG3624 | [CG3624](http://flybase.bio.indiana.edu/cgi-bin/gbrowse/dmel/?name=FBgn0034724) | 956 | 90 |
| [CG3624](http://flybase.bio.indiana.edu/cgi-bin/fbidq.html?FBgn0034724) | CG3624 | [CG3624](http://flybase.bio.indiana.edu/cgi-bin/gbrowse/dmel/?name=FBgn0034724) | 36304 | 14416 |
| CG4814 |  |  | 42353 | 15074 |
| [CG6490](http://flybase.bio.indiana.edu/cgi-bin/fbidq.html?FBgn0039431) | CG6490 | [CG6490](http://flybase.bio.indiana.edu/cgi-bin/gbrowse/dmel/?name=FBgn0039431) | 6683 | 73 |
| [CG6490](http://flybase.bio.indiana.edu/cgi-bin/fbidq.html?FBgn0039431) | CG6490 | [CG6490](http://flybase.bio.indiana.edu/cgi-bin/gbrowse/dmel/?name=FBgn0039431) | 24477 | 73 |
| [CG7607](http://flybase.bio.indiana.edu/cgi-bin/fbidq.html?FBgn0036145) | CG7607 | [CG7607](http://flybase.bio.indiana.edu/cgi-bin/gbrowse/dmel/?name=FBgn0036145) | 9208 | 3870 |
| [CG8964](http://flybase.bio.indiana.edu/cgi-bin/fbidq.html?FBgn0033674) | CG8964 | [CG8964](http://flybase.bio.indiana.edu/cgi-bin/gbrowse/dmel/?name=FBgn0033674) | 29908 | 14400 |
| cont | contactin | CG1084 | 28294/40613 | 12610 |
| [dpr](http://flybase.bio.indiana.edu/cgi-bin/fbidq.html?FBgn0040726) | defective proboscis extension response | [CG13439](http://flybase.bio.indiana.edu/cgi-bin/gbrowse/dmel/?name=FBgn0040726) | 33816 | 15383 |
| [dpr10](http://flybase.bio.indiana.edu/cgi-bin/fbidq.html?FBgn0052057) | dpr10 | [CG32057](http://flybase.bio.indiana.edu/cgi-bin/gbrowse/dmel/?name=FBgn0052057) | 18919 | 6130 |
| [dpr11](http://flybase.bio.indiana.edu/cgi-bin/fbidq.html?FBgn0053202) | dpr11 | [CG33202](http://flybase.bio.indiana.edu/cgi-bin/gbrowse/dmel/?name=FBgn0053202) | 23243 | 13301 |
| [dpr12](http://flybase.bio.indiana.edu/cgi-bin/fbidq.html?FBgn0085414) | dpr12 | [CG34385](http://flybase.bio.indiana.edu/cgi-bin/gbrowse/dmel/?name=FBgn0085414) | 44740 | 15133 |
| [dpr13](http://flybase.bio.indiana.edu/cgi-bin/fbidq.html?FBgn0034286) | dpr13 | [CG33996](http://flybase.bio.indiana.edu/cgi-bin/gbrowse/dmel/?name=FBgn0034286) | 17667 | 8347 |
| [dpr14](http://flybase.bio.indiana.edu/cgi-bin/fbidq.html?FBgn0029974) | dpr14 | [CG10946](http://flybase.bio.indiana.edu/cgi-bin/gbrowse/dmel/?name=FBgn0029974) | 8005 | 2594 |
| [dpr15](http://flybase.bio.indiana.edu/cgi-bin/fbidq.html?FBgn0037993) | dpr15 | [CG10095](http://flybase.bio.indiana.edu/cgi-bin/gbrowse/dmel/?name=FBgn0037993) | 46244 | 16391 |
| [dpr16](http://flybase.bio.indiana.edu/cgi-bin/fbidq.html?FBgn0037295) | dpr16 | [CG12591](http://flybase.bio.indiana.edu/cgi-bin/gbrowse/dmel/?name=FBgn0037295) | 31986 | 7769 |
| [dpr17](http://flybase.bio.indiana.edu/cgi-bin/fbidq.html?FBgn0051361) | dpr17 | [CG31361](http://flybase.bio.indiana.edu/cgi-bin/gbrowse/dmel/?name=FBgn0051361) | 8481 | 2937 |
| [dpr18](http://flybase.bio.indiana.edu/cgi-bin/fbidq.html?FBgn0030723) | dpr18 | [CG14948](http://flybase.bio.indiana.edu/cgi-bin/gbrowse/dmel/?name=FBgn0030723) | 983 | 96 |
| [dpr19](http://flybase.bio.indiana.edu/cgi-bin/fbidq.html?FBgn0032233) | dpr19 | [CG13140](http://flybase.bio.indiana.edu/cgi-bin/gbrowse/dmel/?name=FBgn0032233) | 42789 | 1065 |
| [dpr2](http://flybase.bio.indiana.edu/cgi-bin/fbidq.html?FBgn0053507) | dpr2 | [CG33507](http://flybase.bio.indiana.edu/cgi-bin/gbrowse/dmel/?name=FBgn0053507) | 29741 | 15154 |
| [dpr20](http://flybase.bio.indiana.edu/cgi-bin/fbidq.html?FBgn0035170) | dpr20 | [CG12191](http://flybase.bio.indiana.edu/cgi-bin/gbrowse/dmel/?name=FBgn0035170) | 15254 | 5971 |
| [dpr3](http://flybase.bio.indiana.edu/cgi-bin/fbidq.html?FBgn0053516) | dpr3 | [CG33516](http://flybase.bio.indiana.edu/cgi-bin/gbrowse/dmel/?name=FBgn0053516) | 25110 | 9000 |
| [dpr4](http://flybase.bio.indiana.edu/cgi-bin/fbidq.html?FBgn0053512) | dpr4 | [CG33512](http://flybase.bio.indiana.edu/cgi-bin/gbrowse/dmel/?name=FBgn0053512) | 28518 | 13088 |
| [dpr6](http://flybase.bio.indiana.edu/cgi-bin/fbidq.html?FBgn0040823) | dpr6 | [CG14162](http://flybase.bio.indiana.edu/cgi-bin/gbrowse/dmel/?name=FBgn0040823) | 41161 | 4836 |
| [dpr7](http://flybase.bio.indiana.edu/cgi-bin/fbidq.html?FBgn0053481) | dpr7 | [CG33481](http://flybase.bio.indiana.edu/cgi-bin/gbrowse/dmel/?name=FBgn0053481) | 46216 | 16254 |
| [dpr8](http://flybase.bio.indiana.edu/cgi-bin/fbidq.html?FBgn0052600) | dpr8 | [CG32600](http://flybase.bio.indiana.edu/cgi-bin/gbrowse/dmel/?name=FBgn0052600) | 39203 | 14664 |
| [dpr9](http://flybase.bio.indiana.edu/cgi-bin/fbidq.html?FBgn0038282) | dpr9 | [CG33485](http://flybase.bio.indiana.edu/cgi-bin/gbrowse/dmel/?name=FBgn0038282) | 38690 | 7773 |
| [Dscam](http://flybase.bio.indiana.edu/cgi-bin/fbidq.html?FBgn0033159) | Down syndrome cell adhesion molecule | [CG17800](http://flybase.bio.indiana.edu/cgi-bin/gbrowse/dmel/?name=FBgn0033159) | 3115 | 2596 |
| [ed](http://flybase.bio.indiana.edu/cgi-bin/fbidq.html?FBgn0000547) | echinoid | [CG12676](http://flybase.bio.indiana.edu/cgi-bin/gbrowse/dmel/?name=FBgn0000547) | 938 | 79 |
| [elav](http://flybase.bio.indiana.edu/cgi-bin/fbidq.html?FBgn0000570) | embryonic lethal, abnormal vision | [CG4262](http://flybase.bio.indiana.edu/cgi-bin/gbrowse/dmel/?name=FBgn0000570) | 37915 | 5206 |
| [Fas1](http://flybase.bio.indiana.edu/cgi-bin/fbidq.html?FBgn0000634) | Fasciclin 1 | [CG6588](http://flybase.bio.indiana.edu/cgi-bin/gbrowse/dmel/?name=FBgn0000634) | 23015 | 12817 |
| [Fas2](http://flybase.bio.indiana.edu/cgi-bin/fbidq.html?FBgn0000635) | Fasciclin 2 | [CG3665](http://flybase.bio.indiana.edu/cgi-bin/gbrowse/dmel/?name=FBgn0000635) | 8392 | 2579 |
| [Fas2](http://flybase.bio.indiana.edu/cgi-bin/fbidq.html?FBgn0000635) | Fasciclin 2 | [CG3665](http://flybase.bio.indiana.edu/cgi-bin/gbrowse/dmel/?name=FBgn0000635) | 36350 | 14486 |
| [Fas3](http://flybase.bio.indiana.edu/cgi-bin/fbidq.html?FBgn0000636) | Fasciclin 3 | [CG5803](http://flybase.bio.indiana.edu/cgi-bin/gbrowse/dmel/?name=FBgn0000636) | 42231 | 14367 |
| [fra](http://flybase.bio.indiana.edu/cgi-bin/fbidq.html?FBgn0011592) | frazzled | [CG8581](http://flybase.bio.indiana.edu/cgi-bin/gbrowse/dmel/?name=FBgn0011592) | 6557 | 68 |
| [fred](http://flybase.bio.indiana.edu/cgi-bin/fbidq.html?FBgn0051774) | friend of echinoid | [CG31774](http://flybase.bio.indiana.edu/cgi-bin/gbrowse/dmel/?name=FBgn0051774) | 33298 | 2574 |
| [hbs](http://flybase.bio.indiana.edu/cgi-bin/fbidq.html?FBgn0029082) | hibris | [CG7449](http://flybase.bio.indiana.edu/cgi-bin/gbrowse/dmel/?name=FBgn0029082) | 9471 | 70 |
| hig | hikaru genki | CG2040 | 13266 | 5279 |
| [htl](http://flybase.bio.indiana.edu/cgi-bin/fbidq.html?FBgn0010389) | heartless | [CG7223](http://flybase.bio.indiana.edu/cgi-bin/gbrowse/dmel/?name=FBgn0010389) | 27180 | 14457 |
| [iHog](http://flybase.bio.indiana.edu/cgi-bin/fbidq.html?FBgn0031872) | interference Hedgehog | [CG9211](http://flybase.bio.indiana.edu/cgi-bin/gbrowse/dmel/?name=FBgn0031872) | 29897 | 14317 |
| ImpL2 | Ecdysone-inducible gene L2 | CG15009 | 30930 | 6004 |
| [kek1](http://flybase.bio.indiana.edu/cgi-bin/fbidq.html?FBgn0015399) | kekkon-1 | [CG12283](http://flybase.bio.indiana.edu/cgi-bin/gbrowse/dmel/?name=FBgn0015399) | 36252 | 14381 |
| [kek2](http://flybase.bio.indiana.edu/cgi-bin/fbidq.html?FBgn0015400) | kekkon-2 | [CG4977](http://flybase.bio.indiana.edu/cgi-bin/gbrowse/dmel/?name=FBgn0015400) | 42449 | 9 |
| [kek5](http://flybase.bio.indiana.edu/cgi-bin/fbidq.html?FBgn0031016) | kekkon5 | [CG12199](http://flybase.bio.indiana.edu/cgi-bin/gbrowse/dmel/?name=FBgn0031016) | 1401 | 36 |
| [kek6](http://flybase.bio.indiana.edu/cgi-bin/fbidq.html?FBgn0039862) | kek6 | [CG1804](http://flybase.bio.indiana.edu/cgi-bin/gbrowse/dmel/?name=FBgn0039862) | 19184 | 8810 |
| [kirre](http://flybase.bio.indiana.edu/cgi-bin/fbidq.html?FBgn0028369) | kin of irre | [CG3653](http://flybase.bio.indiana.edu/cgi-bin/gbrowse/dmel/?name=FBgn0028369) | 27227 | 14476 |
| klg | klingon | CG6669 | 36162 | 14314 |
| [Lac](http://flybase.bio.indiana.edu/cgi-bin/fbidq.html?FBgn0010238) | Lachesin | [CG12369](http://flybase.bio.indiana.edu/cgi-bin/gbrowse/dmel/?name=FBgn0010238) | 35524 | 12649 |
| [lea](http://flybase.bio.indiana.edu/cgi-bin/fbidq.html?FBgn0002543) | leak/Robo2 | [CG5481](http://flybase.bio.indiana.edu/cgi-bin/gbrowse/dmel/?name=FBgn0002543) | 11823 | 109 |
| [Nrg](http://flybase.bio.indiana.edu/cgi-bin/fbidq.html?FBgn0002968) | Neuroglian | [CG1634](http://flybase.bio.indiana.edu/cgi-bin/gbrowse/dmel/?name=FBgn0002968) | 6688 | 82 |
| [Nrg](http://flybase.bio.indiana.edu/cgi-bin/fbidq.html?FBgn0002968) | Neuroglian | [CG1634](http://flybase.bio.indiana.edu/cgi-bin/gbrowse/dmel/?name=FBgn0002968) | 107991 | 100482 |
| [nrm](http://flybase.bio.indiana.edu/cgi-bin/fbidq.html?FBgn0005629) | neuromusculin | [CG8779](http://flybase.bio.indiana.edu/cgi-bin/gbrowse/dmel/?name=FBgn0005629) | 979 | 94 |
| [Nrx-IV](http://flybase.bio.indiana.edu/cgi-bin/fbidq.html?FBgn0013997) | Neurexin IV | [CG6827](http://flybase.bio.indiana.edu/cgi-bin/gbrowse/dmel/?name=FBgn0013997) | 8353 | 2436 |
| [Ppn](http://flybase.bio.indiana.edu/cgi-bin/fbidq.html?FBgn0003137) | Papilin | [CG33103](http://flybase.bio.indiana.edu/cgi-bin/gbrowse/dmel/?dontadjust=1&name=FBgn0003137) | 16523 | 6325 |
| [PQBP-1](http://flybase.bio.indiana.edu/cgi-bin/fbidq.html?FBgn0250829) |  | [CG31369](http://flybase.bio.indiana.edu/cgi-bin/gbrowse/dmel/?name=FBgn0250829) | 28752 | 13399 |
| Pxn | Peroxidasin | CG12002 | 15276 | 5987 |
| [robo](http://flybase.bio.indiana.edu/cgi-bin/fbidq.html?FBgn0005631) | roundabout | [CG13521](http://flybase.bio.indiana.edu/cgi-bin/gbrowse/dmel/?name=FBgn0005631) | 47921 | 14414 |
| [robo3](http://flybase.bio.indiana.edu/cgi-bin/fbidq.html?FBgn0041097) | robo3 | [CG5423](http://flybase.bio.indiana.edu/cgi-bin/gbrowse/dmel/?name=FBgn0041097) | 44702 | 14351 |
| [Ror](http://flybase.bio.indiana.edu/cgi-bin/fbidq.html?FBgn0010407) | Ror | [CG4926](http://flybase.bio.indiana.edu/cgi-bin/gbrowse/dmel/?name=FBgn0010407) | 935 | 40 |
| [rst](http://flybase.bio.indiana.edu/cgi-bin/fbidq.html?FBgn0003285) | roughest | [CG4125](http://flybase.bio.indiana.edu/cgi-bin/gbrowse/dmel/?name=FBgn0003285) | 951 | 86 |
| sdk | sidekick | CG5227 | 9437 | 2553 |
| [sev](http://flybase.bio.indiana.edu/cgi-bin/fbidq.html?FBgn0003366) | sevenless | [CG18085](http://flybase.bio.indiana.edu/cgi-bin/gbrowse/dmel/?name=FBgn0003366) | 49924 | 3307 |
| side | sidestep | CG31062 | 1283 | 321 |
| sls | sallimus | CG1915 | 47301 | 9680 |
| [sns](http://flybase.bio.indiana.edu/cgi-bin/fbidq.html?FBgn0024189) | sticks and stones | [CG33141](http://flybase.bio.indiana.edu/cgi-bin/gbrowse/dmel/?name=FBgn0024189) | 877 | 65 |
| [tutl](http://flybase.bio.indiana.edu/cgi-bin/fbidq.html?FBgn0010473) | turtle | [CG15427](http://flybase.bio.indiana.edu/cgi-bin/gbrowse/dmel/?name=FBgn0010473) | 3064 | 2558 |
| [unc-5](http://flybase.bio.indiana.edu/cgi-bin/fbidq.html?FBgn0034013) | unc-5 | [CG8166](http://flybase.bio.indiana.edu/cgi-bin/gbrowse/dmel/?name=FBgn0034013) | 8138 | 3510 |
| [vn](http://flybase.bio.indiana.edu/cgi-bin/fbidq.html?FBgn0003984) | vein | [CG10491](http://flybase.bio.indiana.edu/cgi-bin/gbrowse/dmel/?dontadjust=1&name=FBgn0003984) | 50358 | 2321 |
| [wrapper](http://flybase.bio.indiana.edu/cgi-bin/fbidq.html?FBgn0025878) | wrapper | [CG10382](http://flybase.bio.indiana.edu/cgi-bin/gbrowse/dmel/?name=FBgn0025878) | 101567 | 103858 |
|  |  | CG13992 | 2642 | 932 |
|  |  | CG15744 | 4800 | 2593 |
|  |  | CG13532 | 12848 | 4809 |
|  |  | CG5597 | 12875 | 4822 |
|  |  | CG13672 | 23488 | 13551 |
|  |  | CG33274 | 25365 | 9622 |
|  |  | CG30171 | 29412 | 14839 |
|  |  | CG13134 | 29729 | 15151 |
|  |  | CG34353 | 29845 | 15298 |
|  |  | CG31619 | 33102 | 491 |
|  |  | CG5699 | 34517 | 10855 |
|  |  | CG15630 | 37842 | 5102 |
|  |  | CG15630 | 37843 | 5102 |
|  |  | CG34114 | 38809 | 8414 |
|  |  | CG15354 | 40821 | 9881 |
|  |  | CG16974 | 42226 | 14360 |
|  |  | CG34391 | 42353 | 15074 |
|  | lambik | CG8434 | 43898 | 14407 |
|  | tartan/caps -like | CG11136 | 44991 | 14419 |
|  |  | CG14583 | 49553 | 16410 |
|  |  | CG15312 | 101286 | 105655 |
|  |  | CG31369 | 102322 | 110045 |
|  |  | CG13020 | 104044 | 112547 |

**LRR-domain containing proteins (73)**

| ***Symbol*** | ***Name*** | ***Annotation ID*** | ***VDRC Transformant ID*** | ***VDRC Construct ID*** |
| --- | --- | --- | --- | --- |
| [caps](http://flybase.bio.indiana.edu/cgi-bin/fbidq.html?FBgn0023095) | capricious | [CG11282](http://flybase.bio.indiana.edu/cgi-bin/gbrowse/dmel/?name=FBgn0023095) | 3046 | 2530 |
|  | CG10148 |  | 44841 | 519 |
|  | CG10255 |  | 18600 | 6916 |
|  | CG10307 |  | 27150 | 6935 |
|  | CG11099 |  | 9274 | 1585 |
|  | tartan/caps -like | CG11136 | 44991 | 14419 |
|  | tartan | CG11280 | 5242 | 2450 |
|  | CG11807 |  | 38564 | 7390 |
|  | CG11910 |  | 14737 | 6264 |
|  | CG13125 |  | 17123 | 7927 |
|  | CG13487 |  | 44532 | 2136 |
|  | CG13708 |  | 17690 | 8099 |
|  | CG14185 |  | 32097 | 8250 |
|  | tartan/caps -like | CG14351 | 36220 | 14353 |
|  | CG14662 |  | 8472 | 2804 |
|  | CG14762 |  | 31014 | 5488 |
|  | CG1484 |  | 39528 | 1446 |
|  | CG14995 |  | 12725 | 4711 |
|  | CG1504 |  | 18513 | 8526 |
|  | rdo | CG15151 | 107213 | 103949 |
|  | CG15658 |  | 3040 | 2509 |
|  | CG15744 | [CG15744](http://flybase.bio.indiana.edu/cgi-bin/gbrowse/dmel/?name=FBgn0030466) | 1096 | 97 |
|  | CG17319 |  | 8384 | 2518 |
|  | CG1644 |  | 32691 | 9182 |
|  | CG17667 |  | 36144 | 14305 |
|  | CG18024 |  | 16588 | 5619 |
|  | CG18095 |  | 886 | 1 |
|  | CG18249 |  | 3816 | 2519 |
|  | CG18480 |  | 1071 | 26 |
|  | CG3040 |  | 19219 | 8858 |
|  | CG3095 |  | 14524 | 6373 |
|  | CG31076 |  | 28776 | 13469 |
|  | CG31635 |  | 33979 | 10313 |
|  | CG32085 |  | 34053 | 10452 |
|  | CG32372 |  | 18977 | 5859 |
|  | CG32687 |  | 20819 | 9736 |
|  | CG3408 |  | 36306 | 14429 |
|  | CG3413 |  | 37209 | 2152 |
|  | CG3494 |  | 24760 | 10986 |
|  | CG3980 |  | 34773 | 11179 |
|  | CG4054 |  | 1702 | 431 |
|  | CG4781 |  | 7605 | 2215 |
|  | CG4950 |  | 9931 | 1664 |
|  | CG5096 |  | 27060 | 14378 |
|  | CG5195 |  | 31044 | 5536 |
|  | CG5407 |  | 27410 | 11639 |
|  | toll precursor | CG5490 | 100078 | 103505 |
|  | Toll-9 | CG5528 | 36308 | 14431 |
|  | CG5784 |  | 49386 | 11765 |
|  | CG5810 |  | 44988 | 4955 |
|  | CG5819 |  | 27076 | 14412 |
|  | Gp150 | CG5820 | 900 | 10 |
|  | CG5851 |  | 42051 | 11788 |
|  | CG5888 |  | 12413 | 5404 |
|  | CG6098 |  | 27567 | 11817 |
|  | CG6590/32055 |  | 6335 | 1714 |
|  | CG6860 |  | 7306 | 1753 |
|  | Toll, Tak1 | CG6890 | 9431 | 2510 |
|  | Tehoa, Toll-5 | CG7121 | 17903 | 2534 |
|  | Toll-6 | CG7250 | 27102 | 14438 |
|  | CG7457 |  | 26740 | 12245 |
|  | CG7503 |  | 17898 | 2312 |
|  | CG7509 |  | 51584 | 6018 |
|  | connectin like | CG7702 | 1059 | 21 |
|  | CG7800 |  | 6673 | 2520 |
|  | tartan/caps -like | CG7896 | 36343 | 14469 |
|  | CG8272 |  | 24262 | 13869 |
|  | CG8561 |  | 44361 | 13967 |
|  | Toll-7 | CG8595 | 24473 | 16 |
|  | CG8930 |  | 904 | 15 |
|  | CG9031 |  | 42188 | 14099 |
|  | CG9044 |  | 42193 | 14103 |
| [lbk](http://flybase.bio.indiana.edu/cgi-bin/fbidq.html?FBgn0034083) | lambik | [CG8434](http://flybase.bio.indiana.edu/cgi-bin/gbrowse/dmel/?name=FBgn0034083) | 4319 | 71 |

**Cadherins (19)**

| ***Symbol*** | ***Name*** | ***Annotation ID*** | ***VDRC Transformant ID*** | ***VDRC Construct ID*** |
| --- | --- | --- | --- | --- |
|  |  | CG1744 | 108053 | 106906 |
| [stan](http://flybase.bio.indiana.edu/cgi-bin/fbidq.html?FBgn0024836) | starry night | [CG11895](http://flybase.bio.indiana.edu/cgi-bin/gbrowse/dmel/?name=FBgn0024836) | 1665 | 607 |
| [shg](http://flybase.bio.indiana.edu/cgi-bin/fbidq.html?FBgn0003391) | shotgun | [CG3722](http://flybase.bio.indiana.edu/cgi-bin/gbrowse/dmel/?name=FBgn0003391) | 27081 | 14421 |
| [Ret](http://flybase.bio.indiana.edu/cgi-bin/fbidq.html?FBgn0011829) | Ret oncogene | [CG14396](http://flybase.bio.indiana.edu/cgi-bin/gbrowse/dmel/?name=FBgn0011829) | 843 | 45 |
| [lbm](http://flybase.bio.indiana.edu/cgi-bin/fbidq.html?FBgn0016032) | late bloomer | [CG2374](http://flybase.bio.indiana.edu/cgi-bin/gbrowse/dmel/?name=FBgn0016032) | 7937 | 1843 |
| [ft](http://flybase.bio.indiana.edu/cgi-bin/fbidq.html?FBgn0001075) | fat | [CG3352](http://flybase.bio.indiana.edu/cgi-bin/gbrowse/dmel/?name=FBgn0001075) | 9396 | 881 |
| fat2 | fat2 | CG7794 | 35275 | 12286 |
| [ds](http://flybase.bio.indiana.edu/cgi-bin/fbidq.html?FBgn0000497) | dachsous | [CG17941](http://flybase.bio.indiana.edu/cgi-bin/gbrowse/dmel/?name=FBgn0000497) | 4312 | 2646 |
| CG4655 | CG4655 | CG4655 | 26587 | 11388 |
| Cad74A | Cad74A | CG6445 | 36320 | 14440 |
| [Cad86C](http://flybase.bio.indiana.edu/cgi-bin/fbidq.html?FBgn0037840) | Cad86C | [CG4509](http://flybase.bio.indiana.edu/cgi-bin/gbrowse/dmel/?name=FBgn0037840) | 3744 | 158 |
| [Cad87A](http://flybase.bio.indiana.edu/cgi-bin/fbidq.html?FBgn0037963) | Cad87A | [CG6977](http://flybase.bio.indiana.edu/cgi-bin/gbrowse/dmel/?name=FBgn0037963) | 8578 | 3637 |
|  |  | CG32708 | 49325 | 17452 |
| [Cad88C](http://flybase.bio.indiana.edu/cgi-bin/fbidq.html?FBgn0038247) | Cad88C | [CG3389](http://flybase.bio.indiana.edu/cgi-bin/gbrowse/dmel/?name=FBgn0038247) | 36164 | 14315 |
| [Cad89D](http://flybase.bio.indiana.edu/cgi-bin/fbidq.html?FBgn0038439) | Cad89D | [CG14900](http://flybase.bio.indiana.edu/cgi-bin/gbrowse/dmel/?name=FBgn0038439) | 36331 | 14455 |
| [Cad99C](http://flybase.bio.indiana.edu/cgi-bin/fbidq.html?FBgn0039709) | Cad99C | [CG31009](http://flybase.bio.indiana.edu/cgi-bin/gbrowse/dmel/?name=FBgn0039709) | 3733 | 151 |
| [CadN](http://flybase.bio.indiana.edu/cgi-bin/fbidq.html?FBgn0015609) | Cadherin-N | [CG7100](http://flybase.bio.indiana.edu/cgi-bin/gbrowse/dmel/?name=FBgn0015609) | 1092 | 161 |
| CadN2 | CadN2 | CG7527 | 36166 | 14316 |
| [cals](http://flybase.bio.indiana.edu/cgi-bin/fbidq.html?FBgn0039928) | calsyntenin-1 | [CG11059](http://flybase.bio.indiana.edu/cgi-bin/gbrowse/dmel/?name=FBgn0039928) | 36348 | 14484 |

**Integrins (8)**

| ***Symbol*** | ***Name*** | ***Annotation ID*** | ***VDRC Transformant ID*** | ***VDRC Construct ID*** |
| --- | --- | --- | --- | --- |
| [if](http://flybase.bio.indiana.edu/cgi-bin/fbidq.html?FBgn0001250) | inflated/aPS2 | [CG9623](http://flybase.bio.indiana.edu/cgi-bin/gbrowse/dmel/?name=FBgn0001250) | 44885 | 1175 |
| [mew](http://flybase.bio.indiana.edu/cgi-bin/fbidq.html?FBgn0004456) |  | [CG1771](http://flybase.bio.indiana.edu/cgi-bin/gbrowse/dmel/?name=FBgn0004456) | 44890 | 1230 |
| mew/aPS1 |  | CG1771 | 5671 | 1230 |
| [mys](http://flybase.bio.indiana.edu/cgi-bin/fbidq.html?FBgn0004657) | myospheroid | [CG1560](http://flybase.bio.indiana.edu/cgi-bin/gbrowse/dmel/?name=FBgn0004657) | 29619 | 15002 |
| [scb](http://flybase.bio.indiana.edu/cgi-bin/fbidq.html?FBgn0003328) | scab | [CG8095](http://flybase.bio.indiana.edu/cgi-bin/gbrowse/dmel/?name=FBgn0003328) | 4891 | 2006 |
| [αPS4](http://flybase.bio.indiana.edu/cgi-bin/fbidq.html?FBgn0034005) | αPS4 | [CG16827](http://flybase.bio.indiana.edu/cgi-bin/gbrowse/dmel/?name=FBgn0034005) | 37172 | 2007 |
| [αPS5](http://flybase.bio.indiana.edu/cgi-bin/fbidq.html?FBgn0034880) | αPS5 | [CG5372](http://flybase.bio.indiana.edu/cgi-bin/gbrowse/dmel/?name=FBgn0034880) | 6646 | 2181 |
| [βInt-ν](http://flybase.bio.indiana.edu/cgi-bin/fbidq.html?FBgn0010395) | β^ν^ integrin | [CG1762](http://flybase.bio.indiana.edu/cgi-bin/gbrowse/dmel/?name=FBgn0010395) | 893 | 7 |

**Laminins (9)**

| ***Symbol*** | ***Name*** | ***Annotation ID*** | ***VDRC Transformant ID*** | ***VDRC Construct ID*** |
| --- | --- | --- | --- | --- |
| [crb](http://flybase.bio.indiana.edu/cgi-bin/fbidq.html?FBgn0086905) | crumbs | [CG6383](http://flybase.bio.indiana.edu/cgi-bin/gbrowse/dmel/?name=FBgn0086905) | 39177 | 14463 |
| [drpr](http://flybase.bio.indiana.edu/cgi-bin/fbidq.html?FBgn0027594) | draper | [CG2086](http://flybase.bio.indiana.edu/cgi-bin/gbrowse/dmel/?name=FBgn0027594) | 27086 | 14423 |
| [LanA](http://flybase.bio.indiana.edu/cgi-bin/fbidq.html?FBgn0002526) | Laminin A | [CG10236](http://flybase.bio.indiana.edu/cgi-bin/gbrowse/dmel/?name=FBgn0002526) | 18873 | 6022 |
| [LanB1](http://flybase.bio.indiana.edu/cgi-bin/fbidq.html?FBgn0002527) | Laminin B1 | [CG7123](http://flybase.bio.indiana.edu/cgi-bin/gbrowse/dmel/?name=FBgn0002527) | 23119 | 13179 |
| [LanB2](http://flybase.bio.indiana.edu/cgi-bin/fbidq.html?FBgn0002528) | Laminin B2 | [CG3322](http://flybase.bio.indiana.edu/cgi-bin/gbrowse/dmel/?name=FBgn0002528) | 42559 | 2394 |
| [NetB](http://flybase.bio.indiana.edu/cgi-bin/fbidq.html?FBgn0015774) | Netrin-B | [CG10521](http://flybase.bio.indiana.edu/cgi-bin/gbrowse/dmel/?name=FBgn0015774) | 100840 | 103672 |
| Nrx-1 | Neurexin-1 | CG7050 | 36328 | 14451 |
| [trol](http://flybase.bio.indiana.edu/cgi-bin/fbidq.html?FBgn0001402) |  | [CG33950](http://flybase.bio.indiana.edu/cgi-bin/gbrowse/dmel/?name=FBgn0001402) | 22642 | 12341 |
| [wb](http://flybase.bio.indiana.edu/cgi-bin/fbidq.html?FBgn0004002) | wing blister | [CG15288](http://flybase.bio.indiana.edu/cgi-bin/gbrowse/dmel/?name=FBgn0004002) | 3141 | 1560 |

**Semaphorins (10)**

| ***Symbol*** | ***Name*** | ***Annotation ID*** | ***VDRC Transformant ID*** | ***VDRC Construct ID*** |
| --- | --- | --- | --- | --- |
| [Sema-1a](http://flybase.bio.indiana.edu/cgi-bin/fbidq.html?FBgn0011259) | Sema-1a | [CG18405](http://flybase.bio.indiana.edu/cgi-bin/gbrowse/dmel/?name=FBgn0011259) | 36148 | 14307 |
| [Sema-1b](http://flybase.bio.indiana.edu/cgi-bin/fbidq.html?FBgn0016059) | Sema-1b | [CG6446](http://flybase.bio.indiana.edu/cgi-bin/gbrowse/dmel/?name=FBgn0016059) | 107233 | 104666 |
| [Sema-2a](http://flybase.bio.indiana.edu/cgi-bin/fbidq.html?FBgn0011260) | Sema-2a | [CG4700](http://flybase.bio.indiana.edu/cgi-bin/gbrowse/dmel/?name=FBgn0011260) | 15810 | 5476 |
| [Sema-5c](http://flybase.bio.indiana.edu/cgi-bin/fbidq.html?FBgn0250876) | Semaphorin-5c | [CG5661](http://flybase.bio.indiana.edu/cgi-bin/gbrowse/dmel/?name=FBgn0250876) | 9429 | 2501 |
| [plexA](http://flybase.bio.indiana.edu/cgi-bin/fbidq.html?FBgn0025741) | plexin A | [CG11081](http://flybase.bio.indiana.edu/cgi-bin/gbrowse/dmel/?name=FBgn0025741) | 4740 | 2499 |
| [plexB](http://flybase.bio.indiana.edu/cgi-bin/fbidq.html?FBgn0025740) | plexin B | [CG17245](http://flybase.bio.indiana.edu/cgi-bin/gbrowse/dmel/?name=FBgn0025740) | 8382 | 2500 |
| [dsd](http://flybase.bio.indiana.edu/cgi-bin/fbidq.html?FBgn0039528) | distracted | [CG5634](http://flybase.bio.indiana.edu/cgi-bin/gbrowse/dmel/?name=FBgn0039528) | 1106 | 135 |
|  | CG7166 | [CG7166](http://flybase.bio.indiana.edu/cgi-bin/gbrowse/dmel/?name=FBgn0037107) | 27116 | 14443 |
|  | CG7466 | [CG7466](http://flybase.bio.indiana.edu/cgi-bin/gbrowse/dmel/?name=FBgn0031981) | 42462 | 138 |
|  | CG33960 | [CG33960](http://flybase.bio.indiana.edu/cgi-bin/gbrowse/dmel/?name=FBgn0053960) | 48056 | 16812 |

**Other (41)**

| ***Symbol*** | ***Name*** | ***Annotation ID*** | ***VDRC Transformant ID*** | ***VDRC Construct ID*** |
| --- | --- | --- | --- | --- |
| 18w | 18 wheeler | CG8869 | 963 | 17 |
| arm | armadillo | CG11579 | 7767 | 1372 |
| baz | bazooka | CG5055 | 2914 | 1384 |
| [boss](http://flybase.bio.indiana.edu/cgi-bin/fbidq.html?FBgn0000206) | bride of sevenless | [CG8285](http://flybase.bio.indiana.edu/cgi-bin/gbrowse/dmel/?name=FBgn0000206) | 4365 | 292 |
| cno | canoe | CG2534 | 7769 | 1395 |
|  | CG18146 | [CG18146](http://flybase.bio.indiana.edu/cgi-bin/gbrowse/dmel/?name=FBgn0028939) | 3120 | 2622 |
|  | CG32810 | [CG32810](http://flybase.bio.indiana.edu/cgi-bin/gbrowse/dmel/?name=FBgn0025394) | 18225 | 7621 |
|  | CG7422 | [CG7422](http://flybase.bio.indiana.edu/cgi-bin/gbrowse/dmel/?name=FBgn0035815) | 27997 | 12232 |
|  | CG8942 | [CG8942](http://flybase.bio.indiana.edu/cgi-bin/gbrowse/dmel/?name=FBgn0028545) | 40747 | 14386 |
| [cora](http://flybase.bio.indiana.edu/cgi-bin/fbidq.html?FBgn0010434) | coracle | [CG11949](http://flybase.bio.indiana.edu/cgi-bin/gbrowse/dmel/?name=FBgn0010434) | 9788 | 1405 |
| [crq](http://flybase.bio.indiana.edu/cgi-bin/fbidq.html?FBgn0015924) | croquemort | [CG4280](http://flybase.bio.indiana.edu/cgi-bin/gbrowse/dmel/?name=FBgn0015924) | 45883 | 784 |
| [Csp](http://flybase.bio.indiana.edu/cgi-bin/fbidq.html?FBgn0004179) | Cysteine string protein | [CG6395](http://flybase.bio.indiana.edu/cgi-bin/gbrowse/dmel/?name=FBgn0004179) | 34167 | 10571 |
| [Dl](http://flybase.bio.indiana.edu/cgi-bin/fbidq.html?FBgn0000463) | Delta | [CG3619](http://flybase.bio.indiana.edu/cgi-bin/gbrowse/dmel/?name=FBgn0000463) | 3720 | 146 |
| [dlg1](http://flybase.bio.indiana.edu/cgi-bin/fbidq.html?FBgn0001624) | discs large 1 | [CG1725](http://flybase.bio.indiana.edu/cgi-bin/gbrowse/dmel/?name=FBgn0001624) | 2612 | 855 |
| dsh | dishevelled | CG18361 | 101525 | 108967 |
| [Dg](http://flybase.bio.indiana.edu/cgi-bin/fbidq.html?FBgn0034072) | Dystroglycan | [CG18250](http://flybase.bio.indiana.edu/cgi-bin/gbrowse/dmel/?name=FBgn0034072) | 107029 | 100828 |
| [eag](http://flybase.bio.indiana.edu/cgi-bin/fbidq.html?FBgn0000535) | ether a go-go | [CG10952](http://flybase.bio.indiana.edu/cgi-bin/gbrowse/dmel/?name=FBgn0000535) | 9127 | 3363 |
| [fas](http://flybase.bio.indiana.edu/cgi-bin/fbidq.html?FBgn0000633) | faint sausage | [CG17716](http://flybase.bio.indiana.edu/cgi-bin/gbrowse/dmel/?name=FBgn0000633) | 42236 | 14405 |
| fz | frizzled | CG17697 | 43075 | 4614 |
| [fz2](http://flybase.bio.indiana.edu/cgi-bin/fbidq.html?FBgn0016797) | frizzled 2 | [CG9739](http://flybase.bio.indiana.edu/cgi-bin/gbrowse/dmel/?name=FBgn0016797) | 26928 | 13804 |
| [fz3](http://flybase.bio.indiana.edu/cgi-bin/fbidq.html?FBgn0027343) | frizzled 3 | [CG16785](http://flybase.bio.indiana.edu/cgi-bin/gbrowse/dmel/?name=FBgn0027343) | 30214 | 3174 |
| [fz4](http://flybase.bio.indiana.edu/cgi-bin/fbidq.html?FBgn0027342) | frizzled 4 | [CG4626](http://flybase.bio.indiana.edu/cgi-bin/gbrowse/dmel/?name=FBgn0027342) | 5451 | 3269 |
| [Gli](http://flybase.bio.indiana.edu/cgi-bin/fbidq.html?FBgn0001987) | Gliotactin | [CG3903](http://flybase.bio.indiana.edu/cgi-bin/gbrowse/dmel/?name=FBgn0001987) | 37116 | 1735 |
| [Glt](http://flybase.bio.indiana.edu/cgi-bin/fbidq.html?FBgn0001114) | Glutactin | [CG9280](http://flybase.bio.indiana.edu/cgi-bin/gbrowse/dmel/?name=FBgn0001114) | 15429 | 5017 |
| [Glu-RI](http://flybase.bio.indiana.edu/cgi-bin/fbidq.html?FBgn0004619) | Glutamate receptor I | [CG8442](http://flybase.bio.indiana.edu/cgi-bin/gbrowse/dmel/?name=FBgn0004619) | 44438 | 3582 |
| grk | gurken | CG17610 | 3121 | 2624 |
| [in](http://flybase.bio.indiana.edu/cgi-bin/fbidq.html?FBgn0001259) | inturned | [CG16993](http://flybase.bio.indiana.edu/cgi-bin/gbrowse/dmel/?name=FBgn0001259) | 26133 | 10892 |
| [Nrt](http://flybase.bio.indiana.edu/cgi-bin/fbidq.html?FBgn0004108) | Neurotactin | [CG9704](http://flybase.bio.indiana.edu/cgi-bin/gbrowse/dmel/?name=FBgn0004108) | 8495 | 3597 |
| [N](http://flybase.bio.indiana.edu/cgi-bin/fbidq.html?FBgn0004647) | Notch | [CG3936](http://flybase.bio.indiana.edu/cgi-bin/gbrowse/dmel/?name=FBgn0004647) | 27229 | 14477 |
| [ppk12](http://flybase.bio.indiana.edu/cgi-bin/fbidq.html?FBgn0034730) | pickpocket 12 | [CG10972](http://flybase.bio.indiana.edu/cgi-bin/gbrowse/dmel/?name=FBgn0034730) | 105131 | 101805 |
| [Psn](http://flybase.bio.indiana.edu/cgi-bin/fbidq.html?FBgn0019947) | Presenilin | [CG18803](http://flybase.bio.indiana.edu/cgi-bin/gbrowse/dmel/?name=FBgn0019947) | 43083 | 4624 |
| [santa-maria](http://flybase.bio.indiana.edu/cgi-bin/fbidq.html?FBgn0025697) |  | [CG12789](http://flybase.bio.indiana.edu/cgi-bin/gbrowse/dmel/?name=FBgn0025697) | 33153 | 969 |
| scrib | scribbled | CG5462 | 27424 | 11663 |
| [shakB](http://flybase.bio.indiana.edu/cgi-bin/fbidq.html?FBgn0085387) | shaking B | [CG34358](http://flybase.bio.indiana.edu/cgi-bin/gbrowse/dmel/?name=FBgn0085387) | 24578 | 7794 |
| [shi](http://flybase.bio.indiana.edu/cgi-bin/fbidq.html?FBgn0003392) | shibire | [CG18102](http://flybase.bio.indiana.edu/cgi-bin/gbrowse/dmel/?name=FBgn0003392) | 3799 | 1529 |
| [sli](http://flybase.bio.indiana.edu/cgi-bin/fbidq.html?FBgn0003425) | slit | [CG8355](http://flybase.bio.indiana.edu/cgi-bin/gbrowse/dmel/?name=FBgn0003425) | 38233 | 5822 |
| [syt](http://flybase.bio.indiana.edu/cgi-bin/fbidq.html?FBgn0004242) | synaptotagmin | [CG3139](http://flybase.bio.indiana.edu/cgi-bin/gbrowse/dmel/?name=FBgn0004242) | 8875 | 842 |
| sdc | syndecan | CG10497 | 13322 | 4545 |
| [Ten-a](http://flybase.bio.indiana.edu/cgi-bin/fbidq.html?FBgn0004446) | Tenascin accessory | [CG32659](http://flybase.bio.indiana.edu/cgi-bin/gbrowse/dmel/?name=FBgn0004446) | 8322 | 3330 |
| [Ten-m](http://flybase.bio.indiana.edu/cgi-bin/fbidq.html?FBgn0004449) | Tenascin major | [CG5723](http://flybase.bio.indiana.edu/cgi-bin/gbrowse/dmel/?name=FBgn0004449) | 51173 | 3620 |
| [trp](http://flybase.bio.indiana.edu/cgi-bin/fbidq.html?FBgn0003861) | transient receptor potential | [CG7875](http://flybase.bio.indiana.edu/cgi-bin/gbrowse/dmel/?name=FBgn0003861) | 1365 | 372 |
