# Supplementary material for: Transsynaptic Coordination of Synaptic Growth, Function, and Stability by the L1-Type CAM Neuroglian
Source: PLoS Biol. 2013 Apr 16;11(4):e1001537. doi: 10.1371/journal.pbio.1001537 (PMC3627646; doi:10.1371/journal.pbio.1001537)
Supplement: Table S2 — Original data displayed in Figures 1, 2, 4, and 5 and Figures S6 and S8. (DOCX) [file pbio.1001537.s010.docx]

**Table S2**

Data for Figure 1 and Figure S1

| **RNAi** Retraction frequency [%] | | | | | | | | |
| --- | --- | --- | --- | --- | --- | --- | --- | --- |
| UAS | Gal4 | mu4 | p | mu6/7 | p | mu12 | mu13 | n |
| ctrl | neu^2^ | 0.8 ± 0.8 |  | 0.8 ± 0.8 |  | 0.0 ± 0.0 | 0.0 ± 0.0 | 12 |
| RNAi^1^ | neu^1^ | 30.0 ± 6.8 | ≤ 0.0001 | 23.3 ± 7.1 | 0.0004 | 8.3 ± 4.0 | 20.0 ± 6.3 | 6 |
|  | neu^2^ | 33.4 ± 4.3 | ≤ 0.0001 | 18.3 ± 2.9 | 0.0002 | 27.5 ± 3.7 | 30.4 ± 4.5 | 24 |
|  | neu^3^ | 63.9 ± 3.3 | ≤ 0.0001 | 44.5 ± 4.8 | ≤ 0.0001 | 27.0 ± 3.4 | 35.7 ± 3.9 | 20 |
|  | mus^1^ | 1.7 ± 0.8 | ns | 0.4 ± 0.4 | ns | 0.0 ± 0.0 | 0.0 ± 0.0 | 24 |
|  |  |  |  |  |  |  |  |  |
| RNAi^2^ | neu^2^ | 31.8 ± 5.6 | ≤ 0.0001 | 23.3 ± 5.0 | ≤ 0.0001 | 15.5 ± 2.7 | 22.7 ± 3.6 | 13 |
|  | neu^3^ | 38.6 ± 4.7 | ≤ 0.0001 | 21.4 ± 4.4 | ≤ 0.0001 | 18.4 ± 4.0 | 31.2 ± 4.0 | 12 |
|  | neu^4^ | 51.0 ± 4.5 | ≤ 0.0001 | 41.7 ± 7.5 | ≤ 0.0001 | 33.0 ± 7.0 | 48.5 ± 7.0 | 13 |
|  | mus^1^ | 3.8 ± 2.4 | ns | 6.1 ± 2.1 | ns | 5.3 ± 2.4 | 4.6 ± 2.4 | 13 |
| **RNAi rescue** Retraction frequency [%] | | | | | | | | |
| RNAi^1^;Nrg180 | neu^2^ | 9.3 ± 2.0 | 0.0002 | 5.0 ± 2.9 | 0.0014 | 0.7 ± 0.7 | 1.4 ± 1-0 | 14 |
| RNAi^1^;CD8-GFP | neu^2^ | 56.7 ± 4.6 | 0.0008 | 28.3 ± 4.9 | 0.074 | 35.6 ± 6.3 | 21.1 ± 4.0 | 18 |
| RNAi^1^;FasII | neu^2^ | 37.5 ± 4.3 | ns | 15.0 ± 3.8 | ns | 25.0 ± 6.2 | 20.0 ±7.2 | 12 |
| UAS-FasII | neu^2^ | 4.4 ± 1.6 | ≤ 0.0001 | 0.9 ± 0.9 | ≤ 0.0001 | 0.9 ± 0.9 | 0.0 ± 0.0 | 11 |

**Legend**: ctrl = w^1118^ x *elav^C155^*-Gal4; *ok371*-Gal4, p-values^RNAi^ are in comparison to ctrl, p-values^RNAi rescue^ are in comparison to neu^2^RNAi^1^ (all unpaired student`s t-test). Gal4 drivers: neu^1^ = *elav^C155^*-Gal4, neu^2^ = *elav^C155^*-Gal4; *ok371*-Gal4, neu^3^ = *elav^C155^*-Gal4; UAS-*dcr2*, neu^4^ = *elav^C155^*-Gal4; *sca*-Gal4UAS-*dcr2*, mus^1^ = UAS-*dcr2*; *mef2*-Gal4, n = number of animals (segments A2 to A6 were scored in each animal). Errors represent SEM.

Data for Figure 2

| Genotype | # NMJs | # affected  axons | total # | % affected  axons | p | n |
| --- | --- | --- | --- | --- | --- | --- |
| FRT19A | 290 | 3 | 293 | 1.5 ± 1.0 |  | 23 |
| nrg^14^, FRT19A | 152 | 162 | 314 | 53.5 ± 3.7 | ≤ 0.0001 | 26 |
| nrg^14^, FRT19A; P[nrg_wt] | 167 | 5 | 172 | 2.1 ± 1.0 | 0.65 | 18 |
| nrg^14^, FRT19A; P[nrg167ΔFIQGY] | 141 | 3 | 144 | 1.5 ± 0.7 | 0.34 | 11 |
| nrg^14^, FRT19A; P[nrg180ΔFIQGY] | 209 | 46 | 255 | 18.4 ± 3.3 | 0.0005 | 22 |
| nrg^14^, FRT19A; P[nrgΔIg3/4] | 78 | 69 | 147 | 49.5 ± 4.5 | ≤ 0.0001 | 21 |

**Legend**: p-values are in comparison to FRT19A (all unpaired student`s t-test), n = number of animals. Errors represent SEM.

Description for Figure 2/4

| Pacman mutations | amino acid sequence |
| --- | --- |
| P[nrg_wt] | QFTEDGS**FIGQY**VPGKLQPPVSPQ-/46/-AAGAVA**TYV**. |
| P[nrg180Y-F] | QFTEDGSFIGQ**F**VPGKLQPPVSPQ-/46/-AAGAVATYV. |
| P[nrg180Y-D] | QFTEDGSFIGQ**D**VPGKLQPPVSPQ-/46/-AAGAVATYV. |
| P[nrg180Y-A] | QFTEDGSFIGQ**A**VPGKLQPPVSPQ-/46/-AAGAVATYV. |
| P[nrg180ΔFIQGY] | QFTEDGS**-----**VPGKLQPPVSPQ-/46/-AAGAVATYV. |
| P[nrg180ΔC] | QFTEDGS(****)73. |
| P[nrg167ΔFIQGY] | GMNEDGS**-----**GRKGL. |
| P[nrg180ΔPDZ] | QFTEDGSFIGQYVPGKLQPPVSPQ-/46/-AAGAVA**---.** |

**Legend**: The amino acid sequence of the C-terminus of Nrg180 and Nrg167 are shown with single amino acid changes (bold) and deletions (strikes) highlighted. A period indicates the end of the protein sequence.

Data for Figure 4

| Retraction frequency [%] | | | | | | |
| --- | --- | --- | --- | --- | --- | --- |
| Genotype | mu4 | p | mu6/7 | mu12 | mu13 | n |
| w^1118^ | 1.3 ± 0.6 |  | 2.4 ± 0.8 | 0.4 ± 0.4 | 0.4 ± 0.4 | 25 |
| nrg^14^/y; P[nrg_wt] | 1.3 ± 0.9 | 0.9 * | 0.7 ± 0.7 | 0.0 ± 0.0 | 0.0 ± 0.0 | 15 |
| nrg^14^/y; P[nrg180Y-F] | 3.7 ± 1.8 | 0.3 | 11.0 ± 3.7 | 0.5 ± 0.5 | 2.0 ± 0.9 | 20 |
| nrg^14^/y; P[nrg180Y-D] | 7.5 ± 1.6 | 0.0044 | 8.6 ± 1.8 | 5.5 ± 1.4 | 3.0 ± 1.1 | 20 |
| nrg^14^/y; P[nrg180Y-A] | 8.3 ± 2.7 | 0.013 | 6.7 ± 1.9 | 1.7 ± 1.1 | 2.5 ± 1.3 | 12 |
| nrg^14^/y; P[nrg180ΔFIQGY] | 8.1 ± 1.8 | 0.012 | 11.7 ± 2.2 | 7.4 ± 1.6 | 6.3 ± 1.2 | 28 |
| nrg^14^/y; P[nrg180ΔC] | 11.3 ± 2.3 | 0.00149 | 11.0 ± 1.9 | 4.8 ± 1.5 | 1.4 ± 1.0 | 21 |
| nrg^14^/y; P[nrg167ΔFIQGY] | 0.0 ± 0.0 | 0.0 | 3.2 ± 1.2 | 0.9 ± 0.6 | 0.9 ± 0.6 | 22 |
| nrg^14^/y; P[nrg180ΔPDZ] | 1.6 ± 0.9 | 0.85 | 1.6 ± 0.9 | 0.0 ± 0.0 | 2.6 ± 1.3 | 19 |

**Legend**: p-values are in comparison to nrg^14^/y; P[nrg_wt], * = p-value in comparison to w^1118^ (all unpaired student`s t-test), n = number of analyzed animals (segments A2-A6 were scored in each animal). Errors represent SEM.

Data for Figure 5

| NMJ growth defects | | | | | | | |
| --- | --- | --- | --- | --- | --- | --- | --- |
| Genotype | NMJ length | p | Bouton area | p | Bouton no. | p | n |
| w^1118^ | 106.9 ± 4.4 |  | 6.9 ± 0.3 |  | 16.6 ± 0.3 |  | 123 |
| nrg^14^/y; P[nrg_wt] | 103.5 ± 6.2 |  | 5.7 ± 0.3 |  | 15.7 ± 0.5 |  | 98 |
| nrg^14^/y; P[nrg180Y-F] | 110.7 ± 6.3 | 0.42 | 6.7 ± 0.3 | 0.037 | 18.5 ± 0.5 | 0.0003 | 109 |
| nrg^14^/y; P[nrg180Y-D] | 128.9 ± 6.5 | 0.048 | 5.0 ± 0.2 | 0.56 | 22.0 ± 0.7 | ≤ 0.0001 | 142 |
| nrg^14^/y; P[nrg180Y-A] | 119.9 ± 5.0 | 0.076 | 5.4 ± 0.3 | 0.1 | 20.9 ± 0.8 | ≤ 0.0001 | 69 |
| nrg^14^/y; P[nrg180ΔFIQGY] | 156.6 ± 9.1 | ≤ 0.0001 | 4.3 ± 0.2 | 0.0002 | 29.3 ± 0.9 | ≤ 0.0001 | 112 |
| nrg^14^/y; P[nrg180ΔC] | 155.1 ± 9.4 | ≤ 0.0001 | 4.4 ± 0.2 | 0.0013 | 28.2 ± 0.8 | ≤ 0.0001 | 137 |
| nrg^14^/y; P[nrg167ΔFIQGY] | 100.8 ± 3.9 | 0.71 | 6.2 ± 0.3 | 0.2 | 17.4 ± 0.3 | 0.0048 | 176 |
| nrg^14^/y; P[nrg180ΔPDZ] | 98.1 ± 5.2 | 0.5 | 5.9 ± 0.3 | 0.65 | 17.3 ± 0.4 | 0.016 | 115 |

**Legend**: p-values are in comparison to nrg^14^/y; P[nrg_wt] (all unpaired student`s t-test), n^NMJ length^ = 20 NMJs (segments A3 and A4,), n^bouton area^ = 10 NMJs (segment A3), n^bouton no.^ = number of NMJs as indicated (segments A2-A6 were scored in each animal). Errors represent SEM.

Data for Figure S6

| Genotype | mu4 | p | mu6/7 | mu12 | n |
| --- | --- | --- | --- | --- | --- |
| nrg^14^/y; P[Nrgwt] | 1.33 ± 0.91 |  | 0.67 ± 0.67 | 0.0 ± 0.00 | 15 |
| nrg^14^/y; P[Nrg180ΔFIQGY] | 8.12 ± 1.81 | 0.012 | 11.70 ± 2.20 | 7.41 ± 1.60 | 28 |
| nrg^14^/y; P[Nrgwt] mus^3^ RNAi^2^ | 0.0 ± 0.0 | 0.37 | 8.33 ± 3.07 | 0.0 ± 0.00 | 6 |
| nrg^14^/y; P[Nrg180ΔFIQGY] mus^3^ RNAi^2^ | 27.78 ± 4.65 | 0.0003 | 24.44 ± 5.56 | 36.67 ± 5.27 | 9 |

**Legend:** Retraction frequencies on different muscles are indicated. mus^3^= BG57-Gal4, RNAi^2^ = VDRC107991, p-values are in comparison to controls. n = number of analyzed animals. Errors represent SEM.

Data for Figure S8

| UAS-line | Gal4-line | Bouton number | p | n |
| --- | --- | --- | --- | --- |
| ctrl | ok371 | 16.6 ± 0.38 |  | 123 |
| 10xUASnrg180wt_EGFP | ok371 | 17.06 ± 0.38 | 0.061 | 118 |
| 10xUASnrg180ΔFIGQY_EGFP | ok371 | 30.89 ± 0.69 | ≤ 0.0001 | 114 |

**Legend:** Quantification of bouton number. ctrl = w^1118^ x ok371-Gal4, p-values are in comparison to ctrl (all unpaired student`s t-test). n = number of NMJs analyzes (segment A2-A6 were scored for each animal). Errors represent SEM.
