# Supplementary material for: Transsynaptic Coordination of Synaptic Growth, Function, and Stability by the L1-Type CAM Neuroglian
Source: PLoS Biol. 2013 Apr 16;11(4):e1001537. doi: 10.1371/journal.pbio.1001537 (PMC3627646; doi:10.1371/journal.pbio.1001537)
Supplement: Table S3 — List of primers. (DOCX) [file pbio.1001537.s011.docx]

**Table S3. List of primers**

| *Primer* | *DNA-sequence* | |
| --- | --- | --- |
| Nrg180-ENTR-N-term | 5`CACCATGTGGCGGCAGTCAACG | |
| Nrg180-ENTR-C-term | 5`TTAGACGTAGGTGGCCACG | |
| Nrg180-ENTR-C-term-tagged (HA, EGFP) | 5`GACGTAGGTGGCCACGGCTC | |
| Nrg180Y-F | 5`GGCTCCTTCATTGGCCAATTTGTTCCTGGAAAGCTCC | |
| Nrg180Y-D | 5`GGCTCCTTCATTGGCCAAGACGTTCCTGGAAAGCTCC | |
| Nrg180Y-A | 5`GGCTCCTTCATTGGCCAAGCTGTTCCTGGAAAGCTCC | |
| Nrg180ΔFIGQY | 5`AATTTACCGAGGATGGCTCCGTTCCTGGAAAGCTCCAACC | |
| P[nrg180Y-F] | 5`TCTTTCTAATCCCAGGACAATTTACCGAGGATGGCTCCTTCATTGGCCAATTCGTTCCTGGAAAGCTCCAACCGCCGGTTAGCCCACAGCCACTGAACAATTC | |
| P[nrg180Y-D] | 5`TCTTTCTAATCCCAGGACAATTTACCGAGGATGGCTCCTTCATTGGCCAAGACGTTCCTGGAAAGCTCCAACCGCCGGTTAGCCCACAGCCACTGAACAATTC | |
| P[nrg180Y-A] | 5`TCTTTCTAATCCCAGGACAATTTACCGAGGATGGCTCCTTCATTGGCCAAGCCGTTCCTGGAAAGCTCCAACCGCCGGTTAGCCCACAGCCACTGAACAATTC | |
| P[nrg180ΔFIQGY] | 5`ATATTGTATATATCTTTCTAATCCCAGGACAATTTACCGAGGATGGCTCCGTTCCTGGAAAGCTCCAACCGCCGGTTAGCCCACAGCCACTGAACAATTC | |
| P[nrg180ΔC] | 5`ATATTGTATATATCTTTCTAATCCCAGGACAATTTACCGAGGATGGCTCCTAAGAGGCGTGGCTGGGATTCACTTGCCCCATTGTTCTCCTGATTTTCTA | |
| P[nrg180ΔPDZ] | 5`CCGGAGGAGCAGCTGCCAGCAATGGAGGAGCTGCAGCCGGAGCCGTGGCCCCTGTTGACAATTAATCATCGGCA | |
| P[nrg167ΔFIQGY] | 5`ACAATCACAATCAATATTAAATCGACAACGACAACCAATATCCAGGCATGAATGAAGATGGATCCGGACGCAAAGGACTTTGATTTAATTAGTAAGCAGCGCACCGCAACAGCAA | |
| P[nrgΔIg3/4] | 5`CCTCGGTGTTTCGCAGTGAATACAAGATTGGCAACAAGGTGCTCCTCGATGCTGAGCCGCCAACGATTTCCGAAGCTCCAGCAGCTGTATCCACTGTCGA | |
| *Check and seq primer* | *Forward primer* | *Reverse primer* |
| pENTR_nrg180wt, Y-F, Y-A, Y-D, ΔFIGQY (check mutations in FIGQY motif) | 5`AATCGGGGCGGAAAGTACG | 5`CAGGAAACAGCTATGAC |
| pENTR_nrg180wt (start and stop) | 5`TGTAAAACGACGGCCAGT | 5`CAGGAAACAGCTATGAC |
| pENTR/pUAST nrgwt, Y-F, Y-A, Y-D, ΔFIGQY (sequencing of complete ORF, primer 1-8) | 5`TGCTCTTCAAAGTGGCGC  5`GTTAGTGCCTCGCAGAAC  5`TAACTACGGTTGCAACGC  5`GATTCGTGAAGACCAATG  5`CCGAAATCGAGCACAATG  5`ACAATGGACGCTTCAATG  5`TGGATACGCGAGAATGAG  5`CGATACTGATTCGATGGC | 5`CCCGATCCTCCGGCAGTT  5`CAATGAACCATCCGGCAT  5`CCATCTTCATGCGTGTGA  5`CATTGTGAAGTTGGTGGG  5`GGCGTGAACGATGTATTG  5`CACCGTTAGCTTGGACAT  5`CACGTCGCAGGTGTATGT  5`GTGGCCGTTCCGAATTCA |
| 10xpUAST_Nrg_HA (check) | 5`AATCGGGGCGGAAAGTACG | 5`GGCATTCCACCACTGCTCCC |
| 10xpUAST_Nrg_EGFP (check) | 5`TATAAATAGAGGCGCTTCGT | 5`CAAGTCCGCCATGCCCGAAG |
| P[acman] check primer (Nrg180FIGQY mutations) | 5`AACTGACGCATTTGCCAGG | 5`GCAGACACTTAAAGCAGTT |
| P[acman] seq primer (Nrg180FIGQY mutations) | 5`CATATCATTTTGCACCGGC | 5`ACGATGCTCCACCCGATGCT |
| P[acman] check (ΔC) | 5`AACTGACGCATTTGCCAGG | 5`GCTTTAAATTCATGCGAG |
| P[acman] seq (ΔC) | 5`CATATCATTTTGCACCGGC | 5`GCAGACACTTAAAGCAGTT |
| P[acman] check (ΔPDZ) | 5`GAGGATGGCTCCTTCATTG | 5`GCTTTAAATTCATGCGAG |
| P[acman] seq (ΔPDZ) | 5`CACTGAACAATTCCGCTGC | 5`GCAGACACTTAAAGCAGTT |
| P[acman] check (Nrg167ΔFIGQY) | 5`AGCCACTTGCCGTTATAAG | 5`GGCAGTATTGATTTGCAT |
| P[acman] seq (Nrg167ΔFIGQY) | 5`GTGTTCCTTGTTATGTGT | 5`AGTCGTGGTGTTTGCACTT |
| P[acman] check (NrgΔIg3/4) | 5`CGAGCTGAATGCCTTCAAG | 5`GGAGTTAACATTCAGAATGATGG |
| P[acman] seq (NrgΔIg3/4) | 5`GATCCTGAGGGTAATCTCTG | 5`GTTATTCGATCGCTCCACTG |
